# Thiodiketopiperazines and Alkane Derivatives Produced by the Mangrove Sediment–Derived Fungus *Penicillium ludwigii* SCSIO 41408

**DOI:** 10.3389/fmicb.2022.857041

**Published:** 2022-03-28

**Authors:** Jian Cai, Xueni Wang, Zaizhun Yang, Yanhui Tan, Bo Peng, Yonghong Liu, Xuefeng Zhou

**Affiliations:** ^1^CAS Key Laboratory of Tropical Marine Bio-Resources and Ecology, Guangdong Key Laboratory of Marine Materia Medica, South China Sea Institute of Oceanology, Chinese Academy of Sciences, Guangzhou, China; ^2^College of Earth and Planetary Sciences, University of Chinese Academy of Sciences, Beijing, China; ^3^Guangxi Zhuang Yao Medicine Center of Engineering and Technology, Guangxi University of Chinese Medicine, Nanning, China; ^4^State Key Laboratory for Chemistry and Molecular Engineering of Medicinal Resources, School of Chemistry and Pharmaceutical Sciences, Guangxi Normal University, Guilin, China; ^5^Guangdong Eco-Engineering Polytechnic, Guangzhou, China; ^6^Guangdong Ocean Association, Guangzhou, China; ^7^Wuya College of Innovation, Shenyang Pharmaceutical University, Shenyang, China

**Keywords:** mangrove-sediment-derived fungus, *penicillium ludwigii*, thiodiketopiperazines, PC-3, NF-κB, osteoclast differentiation

## Abstract

A new trithiodiketopiperazine derivative, adametizine C (**1**), and five new alkane derivatives (**7–11**), were isolated from the mangrove sediment–derived fungus *Penicillium ludwigii* SCSIO 41408, together with five known dithiodiketopiperazine derivatives (**2–6**). Their structures were elucidated on the basis of spectroscopic analysis, and the absolute configuration of **1** was determined by X-ray crystallographic analysis. In a variety of bioactivity screening, **1**–**5** exhibited some selective antifungal or antibacterial activities. Compounds **1**–**3** showed cytotoxicity against prostate cancer cell line 22Rv1 with half maximal inhibitory concentration (IC_50_) values of 13.0–13.9 μM; moreover, **3** showed obvious activity against another prostate cancer PC-3 cells with an IC_50_ value of 5.1 μM. Further experiments revealed that **3** could significantly reduce PC-3 cells colony formation and induce apoptosis in a dose-dependent manner. Several compounds also exhibited obvious inhibitory activities of lipopolysaccharide–induced nuclear factor-κB with IC_50_ values range from 8.2 to 21.5 μM, and **1**, **5**, and **9** were further evaluated for their effects on receptor activator of NF-κB ligand (RANKL)-induced osteoclastogenesis. Adametizine C (**1**), with the strongest inhibitory activity against RANKL-induced osteoclast differentiation in bone marrow macrophage cells with 10 μM, was suggested to be the promising lead compound for the treatment of osteoclast-related diseases.

## Introduction

**FIGURE 1:**
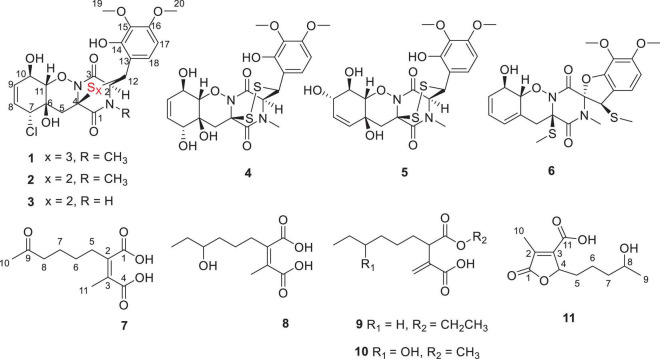
Chemical structures of compounds **1**-**11**.

Mangroves, a special ecosystem characterized by high salinity, muddy or sandy soil, and low pH, as well as partly anoxic and periodically soaked by the tides, play an important role in tropical and subtropical coastal ecosystems. Mangroves nourish various microorganisms due to their complex ecosystem. The variety and complexity of the mangrove soil environment leads to the diversity of soil microorganisms (He et al., 2019). Mangrove soil or sediment-derived microbes play an essential role in maintaining the biosphere balance and are also a prolific source of structurally unique and novel bioactive secondary metabolites (Li et al., 2022). Mainly derived from marine fungi, thiodiketopiperazines have been recently reported to have a broad range of significant biological activities, such as brine shrimp lethality (Liu et al., 2015a), antibacterial (Liu et al., 2015a), antifungal (Liu et al., 2015b), cytotoxic (Yurchenko et al., 2016), and C-terminal inhibitor (Dai et al., 2019) activities. During our ongoing search for novel bioactive secondary metabolites from mangrove fungi (Luo et al., 2018; Luo et al., 2019; Chen et al., 2021a,b; Cai et al., 2021), a new trithiodiketopiperazine, five new alkane derivatives (**7–11**), and five dithiodiketopiperazine derivatives (**2–6**) (Figure 1) were isolated from the mangrove sediment–derived fungus *Penicillium ludwigii* SCSIO 41408. We performed screening for antibacterial and antifungal activities, cytotoxicity against anti-prostate cancer cells, and inhibitory activities of lipopolysaccharide (LPS)–induced nuclear factor-κB (NF-κB) activation. NF-κB exhibited an important role in receptor activator of NF-κB ligand (RANKL)-induced osteoclast differentiation (Hong et al., 2020; Tan et al., 2020; Zhou et al., 2020). Our further research found that Adametizine C (**1**) was suggested to be the promising lead compound for the treatment of osteoclast-related diseases.

## Materials and Methods

### General Experimental Procedures

Optical rotations were measured on a PerkinElmer MPC 500 (Waltham) polarimeter. The UV, IR, and CD spectra were recorded on a Shimadzu UV-2600 PC spectrometer (Shimadzu), an IR Affinity-1 spectrometer (Shimadzu), and a Chirascan circular dichroism spectrometer (Applied Photophysics), respectively. NMR spectra were recorded on a Bruker Avance spectrometer (Bruker) operating at 500 and 700 MHz for ^1^H NMR and 125 and 175 MHz for ^13^C NMR that used tetramethylsilane as an internal standard. High resolution electrospray ionization mass spectroscopy (HRESIMS) spectra were acquired on a Bruker miXis TOF-QII mass spectrometer (Bruker). Column chromatography was performed over silica gel (200–300 mesh) (Qingdao Marine Chemical Factory). Spots were detected on TLC (Qingdao Marine Chemical Factory) under 254-nm UV light. All solvents employed were analytical grade (Tianjin Fuyu Chemical and Industry Factory). Semipreparative HPLC was performed using an octadecylsilyl (ODS) column (YMC-pack ODS-A, YMC Co., Ltd., 10 mm × 250 mm, 5 μm). Artificial sea salt was a commercial product (Guangzhou Haili Aquarium Technology Company).

### Fungal Material

The fungal strain *Penicillium ludwigii* SCSIO 41408 was isolated from a mangrove sediment sample, collected in the Hongsha River estuary to South China Sea, in Sanya city, Hainan Island. The fungus was identified according to the internally transcribed spacer (ITS) region sequence data of the rDNA, and the sequence was deposited in GenBank with the accession number OL823084. The strain was stored on Muller Hinton broth agar (malt extract, 15 g; sea salt, 10 g; agar, 15 g; H_2_O, 1 L; pH 7.4–7.8) at 4°C and deposited in the CAS Key Laboratory of Tropical Marine Bioresources and Ecology, South China Sea Institute of Oceanology, Chinese Academy of Sciences, Guangzhou, China.

### Fermentation, Extraction, and Isolation

The strain *Penicillium ludwigii* SCSIO 41408 was cultured in the seed medium (malt extract, 15 g; sea salt, 10 g; H_2_O, 1 L; pH 7.4–7.8) for 4 days at 28°C on a rotating shaker (180 rpm). A large scale of fermentation was incubated statically at 26°C for 60 days in 1 L × 65 conical flasks with a rice medium (each flack contains 180 g of rice, 3 g of sea salt, 200 ml of H_2_O). The fermented cultures were overlaid and extracted with EtOAc three times to afford a brown extract (346.6 g).

The EtOAc crude extract was chromatographed over a silica gel column eluted with PE/CH_2_Cl_2_ (0–100%, v/v) and CH_2_Cl_2_/MeOH (0–100%, v/v) in a gradient to yield fourteen fractions (Frs. 1–14). Fr. 2 (0.9 g) was separated by semipreparative HPLC (65% MeCN/H_2_O, 2 ml/min) to afford **9** (3.4 mg, *t*_R_ = 17.0 min) and Fr. 2-1. Fr. 2-1 was separated again by semipreparative HPLC (40% MeCN/H_2_O, 2.7 ml/min) to afford **7** (6.7 mg, *t*_R_ = 16.1 min) and **8** (7.5 mg, 14.5 min). Fr. 4 (6.4 g) was separated by semipreparative HPLC (48% MeCN/H_2_O, 3 ml/min) to afford **6** (2.1 mg, *t*_R_ = 17.3 min). Fr. 8 (8.5 g) was purified by semipreparative HPLC (65% MeOH/H_2_O, 2 ml/min) to afford **2** (5.2 mg, *t*_*R*_ = 10.1 min). Fr.10 was divided into four subfractions by ODS silica gel chromatography eluting with MeOH/H_2_O (10–100%). Fr. 10 was divided into four subfractions by Sephadex LH-20. Fr.10-1 was purified by semipreparative HPLC (35% MeCN/H_2_O, 2 ml/min) to afford **11** (10.2 mg, *t*_*R*_ = 8.1 min) and **1** (6.0 mg, *t*_*R*_ = 27.1 min). Fr. 10-4 was purified by semipreparative HPLC (55% MeOH/H_2_O, 3 ml/min) to afford **3** (6.0 mg, *t*_*R*_ = 7.5 min). Fr. 13 was divided into four subfractions by ODS silica gel chromatography eluting with MeOH/H_2_O (10–100%). Fr. 13-1 was further purified by semipreparative HPLC (45% MeOH/H_2_O, 2.7 ml/min) to afford **4** (26.0 mg, *t*_*R*_ = 12.4 min), **5** (5.7 mg, *t*_*R*_ = 14.1 min), and **10** (16.4 mg, *t*_*R*_ = 21.9 min).

*Adametizine C (****1****)*: colorless needles; [α]25 D, −128.5 (c 0.1, MeOH); UV (MeOH) λ_max_ (log ε), 205 (3.52) nm; ECD (0.36 mM, MeOH) λ_max_ (Δε), 203 (−10.98), 242 (−9.06), and 293(+1.33); IRν_max_, 3,352, 2,945, 2,835, 1,670, 1,506, 1,431, 1,250, 1,204, 1,096, 1,018, 831, 795, 677, 601, and 557 cm^–1^; ^1^H and ^13^C NMR, data see Table 1; HRESIMS at *m*/*z* 563.0381 [M + H]^+^ (calculated for C_21_H_24_ClN_2_O_8_S_3_, 563.0378).

**TABLE 1:** ^1^H (700 MHz) and ^13^C (175 MHz) NMR data for compound 1 in DMSO-*d*_6_.

Position	δ_c_, type	δ_H_ (*J* in Hz)	HMBC	COSY
1	162.7, C			
2	66.9, CH	4.63, d (1.1)	3, 12, 13, 21	
3	161.9, C			
4	76.4, C			
5	37.5, CH_2_	α 2.54, dd (14.8, 2.0) β 2.09, d (14.8)	4, 6, 11 1, 4	5b 5a, 6-OH
6	71.4, C			
7	66.8, CH	4.88, m	5, 6, 8, 9	
8	131.5, CH	5.63, dt (10.3, 2.5)	7, 11	
9	127.0, CH	5.58, dt (10.3, 2.0)	6, 10	
10	65.0, CH	4.43, m	9, 8, 11	10-OH, 11
11	87.0, CH	4.10, dd (7.3, 1.9)	5, 6, 10	11
12	56.2, CH	5.39(s)	2, 3, 13, 14, 18	
13	117.8, C			
14	147.5, C			
15	136.1, C			
16	153.4, C			
17	103.4, CH	6.53, d (8.8)	13, 15, 16	18
18	125.5, CH	7.08, d (8.8)	14, 16	17
19	60.8, CH_3_	3.69, s	15	
20	56.1, CH_3_	3.78, s	16, 17	
21 N-CH_3_	32.9, CH_3_	3.12, s	1, 2	
6-OH		6.11, d (2.3)	5, 6, 11	
10-OH		5.17, d (6.6)	8, 10, 11	
14-OH		9.64, s	13, 14, 15	

*2-methyl-3-(5-oxohexyl) maleic acid (****7****)*: colorless oil; UV (MeOH) λ_max_ (log ε), 250 (3.95) and 207 (3.69) nm; IRν_max_, 2,938, 2,866, 1,759, 1,712, 1,362, 1,273, 916, and 735 cm^–1^; ^1^H and ^13^C NMR data, see Tables 2, 3; HRESIMS at *m*/*z* 227.0927 [M-H]^–^ (calculated for C_11_H_15_O_5_, 227.0925).

**TABLE 2:** ^13^C NMR data for compounds 7–11 (δ in ppm) in DMSO-*d*_6_.

Position	7^a^	8^a^	9^b^	10^a^	11*^b^*
1	166.7, C	166.8, C	173.1, C	173.7, C	173.6, C
2	143.7, C	141.4, C	46.5, CH	46.8, CH	133.9, C
3	141.5, C	144.1, C	141.7, C	139.5, C	151.5, C
4	166.9, C	167.0, C	168.1, C	167.7, C	81.7, CH
5	24.0, CH_2_	24.2, CH_2_	31.1, CH_2_	31.0, CH_2_	32.5, CH_2_
6	26.9, CH_2_	23.9, CH_2_	27.0, CH_2_	23.8, CH_2_	21.1, CH_2_
7	23.3, CH_2_	36.5, CH_2_	28.5, CH_2_	36.8, CH_2_	39.0, CH_2_
8	42.7, CH_2_	71.1, CH	22.0, CH_2_	71.3, CH	66.0, CH
9	208.8, C	30.4, CH_2_	30.8, CH_2_	30.4, CH_2_	24.2, CH_3_
10	30.2, CH_3_	10.5, CH_3_	13.9, CH_3_	10.5, CH_3_	10.7, CH_3_
11	9.8, CH_3_	9.8, CH_3_	123.3, CH_2_	126.8, CH_2_	164.2, C
12			59.8, CH_2_	52.1, CH_3_	
13			14.1, CH_3_		

*^a^Data were recorded at 500 MHz.*

*^b^Data were recorded at 700 MHz.*

**TABLE 3:** ^1^H NMR data for compounds 7–11 (δ in ppm) in DMSO-*d*_6_.

Position	7^a^	8^a^	9^b^	10^a^	11^b^
2			3.41, t (3.7)	3.43, t (7.3)	
4					5.13, m
5	2.40, t (6.8)	2.40, m	a 1.71, m b 1.56, m	a 1.78, m b 1.60, m	a 1.97, m b 1.54, m
6	1.48, m	a 1.62, m b 1.50, m	1.23, m	1.23, m	1.36, m
7	1.46, m	1.33, m	1.23, m	1.30, m	1.29, m
8	2.44, t (6.6)	3.30, m	1.23, m	3.28, m	3.55, m
9		1.30, m	1.23, m	1.35, m	1.01, dd (6.1, 1.8)
10	2.07, s	0.83, t (7.4)	0.85, t (7.1)	0.84, t (7.4)	2.02, s
11	2.00, s	1.99, s	a 6.07, s, b 5.50, s,	a 6.23, s b 5.73, s	
12			4.02, q (7.0)	3.59, s	
13			1.13, t (7.1)		
8-OH		4.32, d (5.4)			

*^a^Data were recorded at 500 MHz.*

*^b^Data were recorded at 700 MHz.*

*2-(4-hydroxyhexyl)-3-methylmaleic acid (****8****)*: colorless oil; [α]25 D, −0.7 (c 0.1, MeOH); ECD (2.46 mM, MeOH) λ_max_ (Δε), 203 (−2.49) and 213 (+2.27); UV (MeOH) λ_max_ (log ε), 250 (3.25), 206 (3.65) nm; IRν_max_, 3,390, 2,943, 1,763, 1,682, 1,435, 1,283, 1,202, 1,136, 1,026, 800, and 721 cm^–1^; ^1^H and ^13^C NMR data, see Tables 2, 3; HRESIMS at *m*/*z* 229.1082 [M-H]^–^ (calculated for C_11_H_17_O_5_, 229.1081).

*3-(ethoxycarbonyl)-2-methylenenonanoic acid (****9****)*: brown oil; [α]25 D, +5.9 (c 0.1, MeOH); ECD (3.83 mM, MeOH) λ_max_ (Δε) 200 (−10.62), 214 (+0.68), and 227 (−2.64); UV (MeOH) λ_max_ (log ε), 204 (3.86) nm; IRν_max_, 2,955, 2,928, 2,857, 1,732, 1,715, 1,202, 1,153, 1,113, 1,036, 953, and 835 cm^–1^; ^1^H and ^13^C NMR data, see Tables 2, 3; HRESIMS at *m*/*z* 243.1594 [M + H]^+^ (calculated for C_13_H_23_O_4_, 243.1591).

*7-hydroxy-3-(methoxycarbonyl)-2-methylenenonanoic acid (****10****)*: colorless oil; [α]${}_{D}^{25}$, +1.9 (c 0.1, MeOH); ECD (2.05 mM, MeOH) λ_max_ (Δε), 200 (−3.34), 201 (+4.57), 205 (+2.35), and 211 (−0.98); UV (MeOH) λ_max_ (log ε), 205 (2.93) nm; IRν_max_, 3,447, 2,936, 2,866, 1,717, 1,628, 1,456, 1,435, 1,204, 1,155, 1,024, 9,54.8, and 824 cm^–1^; ^1^H and ^13^C NMR data, see Tables 2, 3; HRESIMS at *m*/*z* 245.1388 [M + H]^+^ (calculated for C_12_H_21_O_5_, 245.1384).

*2-(4-hydroxypentyl)-4-methyl-5-oxo-2,5-dihydrofuran-3-carbo xylic acid (****11****)*: brown oil; [α]${}_{D}^{25}$, +2.6 (c 0.1, MeOH); ECD (4.39 mM, MeOH) λ_max_ (Δε), 200 (−10.62), 214 (+0.68), and 227 (−2.64); UV (MeOH) λ_max_ (log ε), 225 (3.85) nm; IRν_max_, 3,414, 2,932, 1,744, 1,715, 1,337, 1,231, 1,117, 1,024, 947, 764, and 721 cm^–1^; HRESIMS at *m*/*z* 229.1076 [M + H]^+^ (calculated for C_11_H_17_O_5_, 229.1072).

### X-Ray Crystal Structure Analysis

The crystallographic data of compound **1** obtained in MeOH were collected with a Rigaku XtaLAB PRO single-crystal diffractometer using Cu Kα radiation (λ = 1.54184). Briefly, their X-ray crystal structure was solved using SHELXS97, expanded by difference Fourier techniques, and refined by full-matrix least-squares calculation finally. The non-hydrogen atoms were refined anisotropically, and hydrogen atoms were fixed at calculated positions. The crystallographic data of compound **1** have been deposited in the Cambridge Crystallographic Data Centre.

*Crystal Data for adametizine C (****1****)*: C_21_H_31_ClN_2_O_12_S_3_, *M*_*r*_ = 635.11, crystal size 0.1 mm × 0.08 mm × 0.06 mm, orthorhombic, *a* = 9.16490 (10) Å, *b* = 11.25800 (10) Å, *c* = 27.3339 (2) Å, α = β = γ = 90°, *V* = 2407.9 (4) Å^3^, *Z* = 4, *T* = 100.00 (10) K, Space group P2_1_2_1_2_1_, μ = 3.837 mm^–1^, ρ_calc_ = 1.496 g/cm^3^, 14,343 reflections measured (6.468° ≤ 2Θ ≤ 148.482°), 5,546 unique (*R*_int_ = 0.0275, *R*_sigma_ = 0.0325). The final *R*_1_ values were 0.0287 [*I* > 2σ(*I*)]. The final *wR* (*F*^2^) values were 0.0758 [*I* > 2σ(*I*)]. The final *R*_1_ values were 0.0299 (all data). The final *wR* (*F*^2^) values were 0.0764 (all data). The goodness of fit on *F*^2^ was 1.049. The Flack parameter is 0.002 (6) (CDCC 2130918).

### Antibacterial Activity Assay

The antimicrobial activities against five bacteria (*Erysipelothrix rhusiopathiae* WH13013, *Streptococcus suis* SC19, *Escherichia coli* ATCC 25922, *Pseudomonas aeruginosa* ATCC 27853, and *Staphylococcus aureus* ATCC 25923) and four fungi (*Botrytis cinerea*, *Septoria nodorum* Berk., *Fusarium graminearum* Schw., and *Rhizoctonia solani* Kühn) were evaluated using the methods described previously (Wan et al., 2014). Cephalosporin and cycloheximide were used as positive controls against bacteria and fungi, respectively.

### NF-κB Bioassay

The inhibitory activities of LPS-induced NF-κB activation in RAW264.7 cells were evaluated as detected by luciferase reporter gene assay as described previously (Tan et al., 2020). In brief, the RAW264.7 cells stably transfected with a luciferase reporter gene were plated in 96-well plates and then pretreated with tested compounds (20 μM) and BAY11-7082 (NF-κB inhibitor as positive control, 5 μM, Sigma-Aldrich) for 30 min, followed by LPS stimulation (5 μg/ml) for 8 h. Cells were harvested, and luciferase activities of the triplicate tests were measured by the luciferase assay system (Promega, Madison, WI, United States). For further study of compounds **1**, **5**, and **9** on osteoclastogenesis, bone marrow macrophage cells (BMMCs) were added with macrophage-stimulating factor (50 ng/ml) and RANKL (100 ng/ml) stimulation at 5 μM concentrations for 3 days. Then, the cells were fixed and stained for TRAP activity, and the images were photographed by using an inverted microscope (Nikon, Japan). Data are expressed as the mean ± SD and analyzed using GraphPad Prism 7.0 software (San Diego, CA, United States). Statistical differences among groups were performed using one-way analysis of variance with Bonferroni *post hoc* test. A *p*-value of < 0.05 was considered statistically significant.

### Cytotoxicity Bioassay

Cell viability was analyzed by 3-(4,5)-dimethylthiahiazo (-z-y1)-3,5-di-phenytetrazoliumromide (MTT) assay as previous described (Wang et al., 2021). In brief, cells were seeded in 96-well plate at a density of 5 × 10^3^ per well overnight and treated with compounds for demand time. OD_570_ values were detected using a Hybrid Multi-Mode Reader (Synergy H1, BioTek). The experiment was repeated three times independently.

### Plate Clone Formation Assay

PC-3 cells were seeded in six-well plate at a density of 1,000 cell per well overnight, and then, cells were treated with dimethyl sulfoxide (DMSO) (0.1%, v/v), docetaxel (1 μM), and compound **3** (1.25, 2.5, 5, and 10 μM), respectively, for demand time. The cell clone colonies were formed after treating for 2 weeks, and cells were fixed with 4% formaldehyde for 30 min, washed with phosphate buffer saline (PBS) buffer, and then stained with crystal violet stain solution for 30 min. The dye solution was removed, and the cells were washed with PBS buffer again. Cell colonies were recorded and analyzed by the colony count analysis system (GelCount, Oxford Optronix). The experiment was repeated three times independently.

### Apoptosis Assay

PC-3 cells were seeded in six-well plate at a density of 2.0 × 10^5^ cell per well overnight and treated with DMSO (0.1%, v/v), docetaxel (1 μM), and compound **3** (1.25, 2.5, 5, and 10 μM), respectively, for 48 h. Then, cells were collected and stained with annexin V–fluoresceine isothiocyanate (FITC) and propidium iodide (PI) solution following the manufacturer’s manual (BMS500FI-300, Thermo Fisher Scientific). The apoptotic rate of PC-3 cells was examined and analyzed by flow cytometer (NovoCyte, Agilent). Each experiment was repeated three times independently.

## Results and Discussion

### Structural Elucidation

**FIGURE 2:**
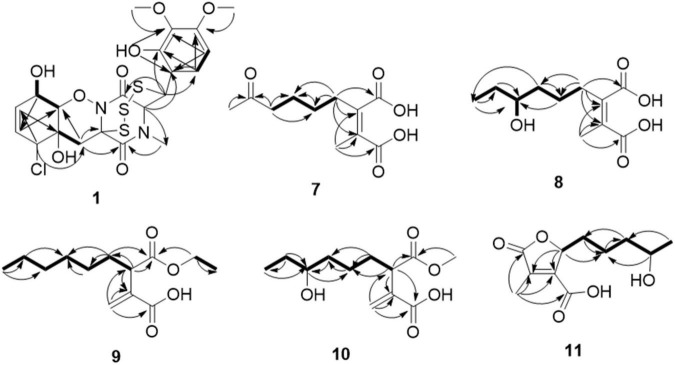
Key HMBC (arrows) and COSY (bold lines) correlations of compounds **1** and **7**-**11**.

**FIGURE 3:**
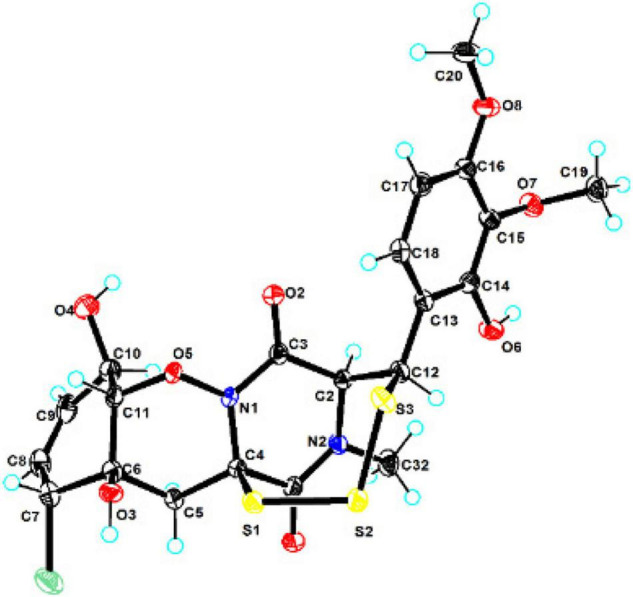
X-ray single-crystal structure of compound **1**.

Compound **1** was obtained as colorless needles and had the molecular formula C_21_H_23_ClN_2_O_8_S_3_ with 11 degrees of unsaturation as established by HRESIMS. The ^1^H-NMR (Table 1) and HSQC experiment of **1** showed the typical pattern of an thiodiketopiperazines skeleton with three exchangeable protons, assigned to 14-OH (δ_H_ 9.64, s), 10-OH (δ_H_ 5.17, d, *J* = 6.6 Hz), and 6-OH (δ_H_ 6.11, d, *J* = 2.3 Hz), two aromatic methine protons [H-17 (δ_H_ 6.53, d, *J* = 8.8 Hz) and H-18 (δ_H_ 7.08, d, *J* = 8.8 Hz)], two olefinic protons [H-8 (δ_H_ 5.63, dt, *J* = 10.3, 2.5 Hz) and H-9 (δ_H_ 5.58, dt, *J* = 10.3, 2.0 Hz)], five methines [H-2 (δ_H_ 4.63, d, *J* = 1.1 Hz), H-7 (δ_H_ 4.88, m), H-10 (δ_H_ 4.43, m), H-11 (δ_H_ 4.10, dd, *J* = 7.3, 1.9 Hz), and H-12 (δ_H_ 5.39, s)], one methylene [H_2_-5α (δ_H_ 2.54, dd, *J* = 14.8, 2.0 Hz) and H_2_-5β (δ_H_ 2.09, d, *J* = 14.8 Hz)], one *N*-methyl [H_3_-21 (δ_H_ 3.12, s)], and two *O*-methyl [H_3_-19 (δ_H_ 3.69, s) and H_3_-20 (δ_H_ 3.78, s)]. Besides the above 13 corresponding hydrogen-bearing carbons, eight non-protonated (with six sp^2^ and two sp^3^) carbon atoms remained in the ^13^C NMR spectrum. Detailed analysis of the above NMR data and 2D NMR correlations (Figure 2 and Table 1) resulted in the elucidation of the planar structure of **1**. Upon slow evaporation of the solvent MeOH, which was achieved by storing the sample in a refrigerator for 4 weeks, single crystals of adequate quality of **1** were obtained, making an X-ray diffraction study possible that could unequivocally confirm the chemical structure of **1**. The absolute configuration was determined on the basis of measuring the anomalous dispersion effects by collecting Friedel pair reflections in the X-ray diffraction experiment (Figure 3).

Compound **7** was isolated as colorless oil, and its molecular formula was determined as C_11_H_16_O_5_ with four degrees of unsaturation, as a deprotonated ion peak at *m*/*z* 227.0927 [M-H]^–^ in the HRESIMS spectrum. The NMR data of **7** (Tables 2, 3) indicated the presence of a ketone group (δ_C_ 208.8), two carboxylic groups (δ_C_ 166.7, 166.9), one double bond (δ_C_ 143.7, 141.5), two methyl group (δ_C_ 30.2, 9.8), and four aliphatic methylene groups. Through inspection of the ^1^H-^1^H COSY spectrum, we easily established a long spin system that started from H_2_-5 (δ_H_ 2.40, t) and terminated at H_2_-8 (δ_H_ 2.44, t) (Figure 2). In the HMBC spectrum, the H_2_-5 showed correlations to the double bond (C-2/C-3), as well as to carboxylic group (C-1, δ_C_ 166.7), indicating that C-5 and C-1 were attached to the allylic carbon (C-2). The HMBC correlations from H_3_-11 to double bond (C-3/C-2), as well as to C-4, indicating that C-11 and C-4 were attached to the allylic carbon (C-2). C-9 connected to C-10 and C-8 was substantiated by the HMBC correlations from H_2_-8 to C-9 and from H_3_-10 to C-9 and C-8. Therefore, **7** was established as 2-methyl-3-(5-oxohexyl) maleic acid.

Compound **8** exhibited UV maximum absorption at 206 and 250 nm similar to that of **7**, indicating that they shared a similar chromophore. The HRESIMS data of **8** determined the molecular formula C_11_H_18_O_5_, with two hydrogen atoms more than **7**. Compared to the NMR data, **8** contained one less carbonyl group at C-9, but one more hydroxyl group at C-8 than **7**. Thus, **8** was determined as 2-(4-hydroxyhexyl)-3-methylmaleic acid.

Compound **9** showed a prominent peak at *m*/*z* 243.1594 [M + H]^+^ in the HRESIMS spectrum, corresponding to the molecular formula C_13_H_23_O_4_. Analysis of the NMR data (Tables 2, 3) of **9** revealed that it was also structurally related to **7**. Compound **9** established a long chain from C-5 to C-10 through COSY spectrum (Figure 2). The position of the double bond is at C-3/C-11 and an ethyl group attached to the ester group (C1). Thus, compound **9** was determined as 3-(ethoxycarbonyl)-2-methylenenonanoic acid.

Compound **10**, a colorless oil, was found to have the molecular formula C_12_H_21_O_5_ on the basis of HRESIMS data. In addition, **10** exhibited a similar UV maximum absorption as **9** at 206 nm, indicating that they have similar chromophores. The NMR spectrum of **10** (Tables 2, 3) is similar to that of **9**, with one more oxygen atom and one methylene group. The ^1^H-^1^H COSY correlations (Figure 2) of H_2_-7/H-8 and H-8/H_2_-9, as well as HMBC correlations from H-8 to C-6 and from C-8 to H_3_-10, H_2_-9, and H_2_-7 indicated that the position of the oxymethine (δ_H_ 3.28; δ_C_ 71.3). Thus, **10** was elucidated as 7-hydroxy-3-(methoxycarbonyl)-2-methylenenonanoic acid.

Compound **11** was obtained as brown oil. HREIMS ion peak at *m*/*z* 229.1076 [M + H]^+^ gave the molecular formula C_11_H_17_O_5_, suggesting four degrees of unsaturation. The ^13^C NMR spectrum (Table 2) revealed two carbonyl carbons (δ_C_ 173.6 and 164.2), together with a fully substituted double bond (δ_C_ 133.9 and 151.5). The ^1^H–^1^H COSY spectrum (Figure 2) revealed a spin system consistent with an *n*-pentyl chain (from C-5 to C-9). An oxymethine of C-8 (δ_H_ 3.55; δ_C_ 66.0) had HMBC correlations extending to C-6 (δ_C_ 21.1). The HMBC correlations from δ_H_ 5.13 (1H, m, H-4) to C-2 (δ_C_ 133.9) and C-3 (δ_C_ 151.5) constructed an α, β-unsaturated five-member lactone ring. In addition, a methyl group substituted at C-2 was supported by the HMBC correlations of C-10 (δ_H_ 2.02, δ_C_ 10.7) to C-1, C-2, and C-3, and a carboxyl substituted at C-3 was supported by the correlations of H-10 to C-11. Finally, compound **11** was identified and named 2-(4-hydroxypentyl)-4-methyl-5-oxo-2,5-dihydrofuran-3-carboxylic acid.

The optical rotations of compounds **8**–**11** were close to zero, and these compounds showed little cotton effect in CD spectroscopy, suggesting them to be racemic mixtures (Supplementary Material). The α-hydro-carbon and C-8 with hydroxyl groups led to chiral centers. It had been reported in the literature that the alkane derivatives were found as enantiomers (Akone et al., 2014).

In addition, the known thiodiketopiperazine derivatives were elucidated as adametizine A (**2**) (Liu et al., 2015a), DC1149B (**3**) (Yamazaki et al., 2015), outovirin B (**4**) (Kajula et al., 2016), pretrichodermamide E (**5**) (Yurchenko et al., 2016), and peniciadametizine A (**6**) (Liu et al., 2015b), respectively, by comparing their physicochemical properties and spectroscopic data with the reported literature values.

### Bioassays of Compounds

All obtained compounds were evaluated for their antibacterial and antifungal activities. Compounds **1–5** exhibited weak antibacterial activities against *Erysipelothrix rhusiopathiae* WH13013 and *Streptococcus suis* SC19, with the minimal inhibitory concentration (MIC) values of 50–100 μg/ml. Compound **2** also exhibited activity against fungi *Botrytis cinerea* and *Septoria nodorum* Berk., with the MIC values of 25 μg/ml. However, all isolated compounds showed no activities against the other three bacteria (*Escherichia coli* ATCC 25922, *Pseudomonas aeruginosa* ATCC 27853, and *Staphylococcus aureus* ATCC 25923) and two fungi (*Fusarium graminearum* Schw. and *Rhizoctonia solani* Kühn). Cephalosporin and cycloheximide were used as the positive controls in the antibacterial (MIC values of 0.78 μg/ml) and antifungal (MIC values of 6.25 μg/ml) tests, respectively (Table 4).

**TABLE 4:** Antibacterial, antifungal, cytotoxic, and anti-inflammatory activities of the obtained compounds.

Comp.	Antibacterial (MIC, μg/mL)		Antifungal (MIC, μg/mL)		Cytotoxic (IC_50_, μM)		Antiinflammatory (IC_50_, μM)
	*E. rhusiopathiae*	*S. suis*	*B. cinerea*	*S. nodorum*	22Rv1	PC-3	NF-κB
**1**	50	100	>100	>100	13.9	44.0	8.2
**2**	100	>100	25	25	13.0	>50	15.1
**3**	50	50	>100	>100	13.6	5.1	>50
**4**	100	100	>100	>100	>50	>50	>50
**5**	50	100	>100	>100	>50	>50	12.6
**6**	>100	>100	>100	>100	>50	>50	>50
**7**	>100	>100	>100	>100	>50	>50	>50
**8**	>100	>100	>100	>100	>50	>50	>50
**9**	>100	>100	>100	>100	>50	>50	10.7
**10**	>100	>100	>100	>100	>50	>50	>50
**11**	>100	>100	>100	>100	>50	>50	21.5
Pos.	0.78	0.78	6.25	6.25	/	/	/

**FIGURE 4:**
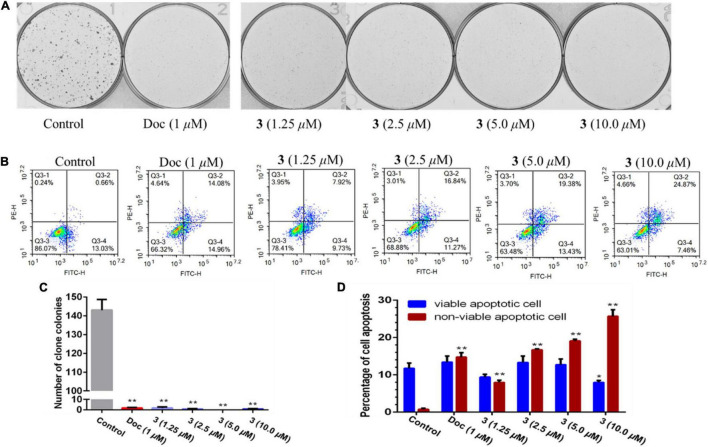
Compound **3** reduced PC-3 cells colony formation **(A,C)** and induced apoptosis **(B,D)**. All results were presented as mean ± SD. Statistical significance was determined with one-way ANOVA. *P < 0.05 and **P < 0.01 were considered statistically significant.

Two human prostate cancer cell lines, PC-3 (androgen receptor negative) and 22Rv1 (androgen receptor positive), were used in the cytotoxicity tests. Compounds **1–3** exhibited cytotoxicity against 22Rv1 cells with half maximal inhibitory concentration (IC_50_) values of 13.9, 13.0, and 13.6 μM, respectively, whereas **1** and **3** showed activities against PC-3 cells with IC_50_ values of 44.0 and 5.1 μM, respectively. Compound **3** was further evaluated for its anti-tumor effect by plate clone formation assay and flow cytometry on PC-3 cells. The results showed that **3** reduced PC-3 cells colony formation (Figures 4A,C) and induced cell apoptosis in a dose-dependent manner (Figures 4B,D).

**FIGURE 5:**
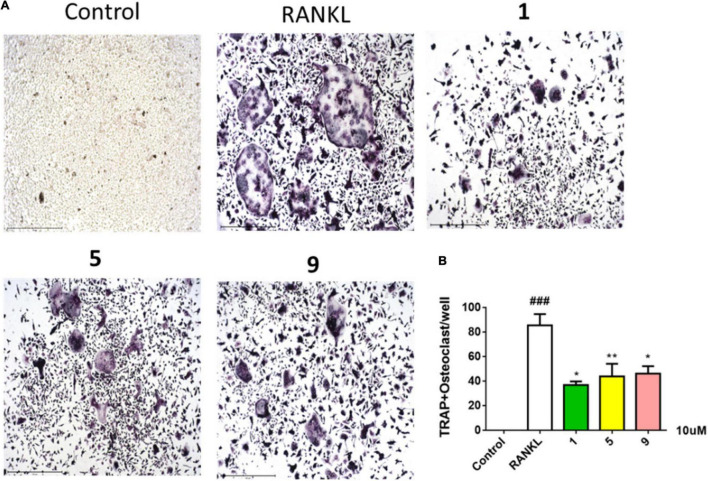
Compounds **1**, **5**, and **9** suppressed RANKL-induced osteoclast differentiation. Representative images of osteoclasts from BMMCs treated with **1**, **5**, and **9** (10 μM) for 3 days, tartrate-resistant acidic phosphatase (TRAP)–positive multinucleated cells were regarded as osteoclasts **(A)** and quantified **(B)**. All experiments were performed at least three times. The data are presented as the mean ± SD of representative experiments. ^###^*p* < 0.001 vs. control group; *p < 0.05 and **p < 0.01 vs. RANKL group.

Compounds **1–11** were screened for their inhibitory activities of LPS-induced NF-κB activation in RAW264.7 cells. Compounds **1**, **2**, **5**, **9**, and **11** exhibited obvious inhibitory activities against LPS-induced NF-κB with IC_50_ values of 8.2, 15.1, 12.6, 10.7, and 21.5 μM, respectively. Moreover, in the further study for evaluation with their effects on RANKL-induced osteoclastogenesis, **1**, **5**, and **9** could suppress the RANKL-induced osteoclast differentiation in BMMCs obviously, with the concentration of 10 μM. The new trithiodiketopiperazine derivative adametizine C (**1**) showed the strongest activity, relatively (Figure 5). Consequently, it is revealed that these compounds could be the promising osteoclast differentiation inhibitors for the treatment of osteoclast-related diseases.

In addition, we also found that the thiodiketopiperazine derivatives exhibited various activities reported in the literatures. For example, pretrichodermide A was active against *Mycobacterium tuberculosis* (Seephonkai et al., 2006); Adametizine A (**2**) was found to be active against a variety of bacteria (Liu et al., 2015a); Outovirin C was active against fungus *Botrytis cinerea* (Kajula et al., 2016). Gliovirin showed inhibitory effects on the expression of cytokines [tumor necrosis factor (TNF)-α and interleukin-2 (IL-2)] and pro-inflammatory enzymes [cyclooxygenase-2 (COX-2) and inducible nitric oxide synthase (INOS)] in T cells and monocytes/macrophages (Rether et al., 2007).

## Conclusion

In summary, chemical investigation of the mangrove sediment–derived fungus *Penicillium ludwigii* SCSIO41408 led to the isolation of a new trithiodiketopiperazine, five new alkane derivatives (**7–11**), and five dithiodiketopiperazine derivatives (**2–6**). In a variety of bioactivity screening, **1**–**5** exhibited some selective antifungal or antibacterial activities; **1**–**3** showed cytotoxicity against prostate cancer cell line 22Rv1or PC-3 cells; moreover, **3** could significantly reduce PC-3 cells’ colony formation and induce apoptosis in a dose-dependent manner. Several compounds also exhibited obvious inhibitory activities of LPS-induced NF-κB, and **1**, **5**, and **9** were suppressed RANKL-induced osteoclast differentiation in BMMCs at 10 μM. Adametizine C (**1**), with the strongest inhibitory activity against RANKL-induced osteoclast differentiation, was suggested to be the promising lead compound for the treatment of osteoclast-related diseases.

## Data Availability Statement

The datasets presented in this study can be found in online repositories. The names of the repository/repositories and accession number(s) can be found in the article/Supplementary Material.

## Author Contributions

JC, BP, YL, and XZ contributed to the conception and design of the study. JC performed the experiments, analyzed data, and wrote the manuscript. XZ reviewed and revised the manuscript. XW and ZY performed the cytotoxicity against 22Rv1 and PC-3 cells. YT did the inhibitory activities of LPS-induced NF-κB activation. All authors contributed to manuscript revision and reviewed and approved the submitted version.

## Conflict of Interest

The authors declare that the research was conducted in the absence of any commercial or financial relationships that could be construed as a potential conflict of interest.

## Publisher’s Note

All claims expressed in this article are solely those of the authors and do not necessarily represent those of their affiliated organizations, or those of the publisher, the editors and the reviewers. Any product that may be evaluated in this article, or claim that may be made by its manufacturer, is not guaranteed or endorsed by the publisher.
