# Supplementary material for: Thiodiketopiperazines and Alkane Derivatives Produced by the Mangrove Sediment–Derived Fungus Penicillium ludwigii SCSIO 41408
Source: Front Microbiol. 2022 Mar 28;13:857041. doi: 10.3389/fmicb.2022.857041 (PMC8996154; doi:10.3389/fmicb.2022.857041)
Supplement: Supplementary file 1 [file Data_Sheet_1.PDF]

Supplementary Material

Thiodiketopiperazines and Alkane Derivatives Produced by the Mangrove Sediment-Derived Fungus *Penicillium ludwigii* SCSIO 41408

Jian Cai [1,2], Xueni Wang [1], Zaizhun Yang [3], Yanhui Tan [4*], Bo Peng [5,6*], Yonghong Liu [1,7], Xuefeng Zhou [1,2*]

[1] CAS Key Laboratory of Tropical Marine Bio-resources and Ecology, Guangdong Key Laboratory of Marine Materia Medica, South China Sea Institute of Oceanology, Chinese Academy of Sciences, Guangzhou, China
[2] University of Chinese Academy of Sciences, Beijing, China
[3] Guangxi Zhuang Yao Medicine Center of Engineering and Technology, Guangxi University of Chinese Medicine, Nanning, China
[4] State Key Laboratory for Chemistry and Molecular Engineering of Medicinal Resources, School of Chemistry and Pharmaceutical Sciences, Guangxi Normal University, Guilin, China
[5] Guangdong Eco-Engineering Polytechnic, Guangzhou, China
[6] Guangdong Ocean Association, Guangzhou 510245, China
[7] Wuya College of Innovation, Shenyang Pharmaceutical University, Shenyang, China

*** Correspondence:**
tyh533@126.com (Yanhui Tan);

pengbo@gig.ac.cn (Bo Peng);

xfzhou@scsio.ac.cn (Xuefeng Zhou), Tel./Fax: +86-20-8902-3174;

Keywords: Mangrove-sediment-derived fungus; *Penicillium ludwigii*; Thiodiketopiperazines; PC-3; NF-*κ*B; osteoclast differentiation

Table of Contents

The physicochemical data of the known compounds 2–6 4
Fig. S1 1H NMR spectra of 1 in DMSO-d_6. 6
Fig. S2 ^{13}C and DEPT NMR spectra of **1** in DMSO-d_6 6
Fig. S3 HSQC spectrum of **1** in DMSO-d_6 7
Fig. S4 HMBC spectrum of **1** in DMSO-d_6. 7
Fig. S5 COSY spectrum of **1** in DMSO-d_6. 8
Fig. S6 HRESIMS spectrum of compound **1**. 8
Fig. S7 The UV spectrum of **1**. 9
Fig. S8 The IR spectrum of **1**. 9
Fig. S9 The CD spectrum of **1** in MeOH 10
Fig. S10 ^{1}H NMR spectrum of **7** in DMSO-d_6 10
Fig. S11 ^{13}C and DEPT NMR spectra of **7** in DMSO-d_6 11
Fig. S12 HSQC spectrum of **7** in DMSO-d_6 11
Fig. S13 HMBC spectrum of **7** in DMSO-d_6 12
Fig. S14 COSY spectrum of **7** in DMSO-d_6 12
Fig. S15 HRESIMS spectrum of compound **7**. 13
Fig. S16 The UV spectrum of **7**. 13
Fig. S17 The IR spectrum of **7**. 14
Fig. S18 ^{1}H NMR spectrum of **8** in DMSO-d_6 14
Fig. S19 ^{13}C and DEPT NMR spectra of **8** in DMSO-d_6 15
Fig. S20 HSQC spectrum of **8** in DMSO-d_6 15
Fig. S21 HMBC spectrum of **8** in DMSO-d_6 16
Fig. S22 COSY spectrum of **8** in DMSO-d_6 16
Fig. S23 HRESIMS spectrum of compound **8**. 17
Fig. S24 The UV spectrum of **8**. 17
Fig. S25 The IR spectrum of **8**. 18
Fig. S26 The CD spectrum of **8** in MeOH. 18
Fig. S27 ^{1}H NMR spectrum of **9** in DMSO-d_6 19
Fig. S28 ^{13}C and DEPT NMR spectra of **9** in DMSO-d_6. 19
Fig. S29 HSQC spectrum of 9 in DMSO-d6 20
Fig. S30 HMBC spectrum of **9** in DMSO-d_6. 20
Fig. S31 COSY spectrum of **9** in DMSO-d_6. 21
Fig. S32 HRESIMS spectrum of compound **9**. 21
Fig. S33 The UV spectrum of **9**. 22
Fig. S34 The IR spectrum of **9**. 22
Fig. S35 The CD spectrum of **9** in MeOH. 23
Fig. S36 ^{1}H NMR spectrum of **10** in DMSO-d_6 23
Fig. S37 ^{13}C and DEPT NMR spectra of **10** in DMSO-d_6 24
Fig. S38 HSQC spectrum of **10** in DMSO-d_6 24
Fig. S39 HMBC spectrum of **10** in DMSO-d_6 25
Fig. S40 COSY spectrum of **10** in DMSO-d_6 25
Fig. S41 HRESIMS spectrum of compound **10**. 26

Fig. S42 The UV spectrum of **10**. 26
Fig. S43 The IR spectrum of **10**. 27
Fig. S44 The CD spectrum of **10** in MeOH. 27
Fig. S45 ^{1}H NMR spectrum of **11** in DMSO-*d*$_6$. 28
Fig. S46 ^{13}C and DEPT NMR spectra of **11** in DMSO-*d*$_6$. 28
Fig. S47 HSQC spectrum of **11** in DMSO-*d*$_6$. 29
Fig. S48 HMBC spectrum of **11** in DMSO-*d*$_6$. 29
Fig. S49 COSY spectrum of **11** in DMSO-*d*$_6$. 30
Fig. S50 HRESIMS spectrum of compound **11**. 30
Fig. S51 The UV spectrum of **11**. 31
Fig. S52 The IR spectrum of **11**. 32
Fig. S53 The CD spectrum of **11** in MeOH. 32

The physicochemical data of the known compounds 2–6

Adametizine A ***(2)***[1]**:** white powder; HRESIMS *m/z* 531.0654 [M + H] $^{+}$ (calcd for $C_{21}H_{24}ClN_2O_8S_2$, 531.0657); $[\alpha]_D^{25}$-165.6 (c 0.1， MeOH); ECD (0.38 mM, MeOH) λ_{max} ($\Delta\varepsilon$) 217(-31.26), 262(-7.09), 301(+0.74); ^{1}H NMR (500 MHz, DMSO-d_6) δ 9.48 (s, 1H, 14-OH), 7.34 (d, *J* = 8.7 Hz, 1H, H-18), 6.55 (d, *J* = 8.9 Hz, 1H, H-17), 5.73 (s, 1H , 6-OH), 5.62 (dt, *J* = 10.4, 2.3 Hz, 1H, H-9), 5.57 (dt, *J* = 10.4, 1.9 Hz, 1H, H-8), 5.37 (d, *J* = 6.9 Hz, 1H, 10-OH), 4.87 (d, *J* = 2.4 Hz, 1H, H-7), 4.62 (s, 1H, H-2), 4.62 (s, 1H, H-12), 4.34 (m, 1H, H-10), 4.04 (dd, *J* = 7.4, 1.8 Hz, 1H, H-11), 3.78 (s, 3H, H-20), 3.68 (s, 3H, H-19), 3.00 (s, 3H, H-21), 2.26 (d, *J* = 15.5 Hz, 1H, H-5β), 2.08 (dd, *J* = 15.6, 1.8 Hz, 1H, H-5α); ^{13}C NMR (125 MHz, DMSO) δ 165.3 (C, C-1), 163.9 (C, C-3), 152.9 (C, C-16), 147.7 (C, C-14) 135.8 (C, C-15), 131.2 (CH, C-9), 126.5 (CH, C-8), 122.9 (CH, C-18), 116.3 (C, C-13), 103.2 (CH, C-17), 85.5 (CH, C-11), 70.1 (C, C-6), 69.1 (C, C-4), 67.1 (CH, C-7), 65.3 (CH, C-2), 64.2 (CH, C-10), 60.3 (CH_3, C-19), 55.7 (CH_3, C-20), 41.2 (CH, C-21), 33.5 (CH_2, C-5), 32.6 (CH_3, C-21).

DC1149B ***(3)***[2]**:** white powder; HRESIMS *m/z* 517.0504 [M + H] $^{+}$ (calcd for $C_{20}H_{22}ClN_2O_8S_2$, 517.0501); $[\alpha]_D^{25}$-166.8 (c 0.1， MeOH); ECD (0.58 mM, MeOH) λ_{max} ($\Delta\varepsilon$) 215(-41.44), 260(-6.34), 293(+1.99); ^{1}H NMR (500 MHz, DMSO-d_6) δ 9.45 (s, 1H, 14-OH), 9.13 (d, *J* = 4.7 Hz, 1H, 2-NH), 7.45 (d, *J* = 8.7 Hz, 1H, H-18), 6.57 (d, *J* = 8.8 Hz, 1H, H-17), 5.68 (s, 1H, 6-OH), 5.65 (dt, *J* = 10.3, 2.4 Hz, 1H, H-8), 5.59 (dt, *J* = 10.4, 2.0 Hz, 1H, H-9), 5.41 (d, *J* = 6.9 Hz, 1H, 10-OH), 4.89 (m, *J* = 2.5 Hz, 1H, H-7), 4.53 (d, *J* = 2.9 Hz, 1H, H-12), 4.46 (dd, *J* = 4.8, 3.2 Hz, 1H, H-2), 4.37 (m, 1H, H-10), 4.07 (dd, *J* = 7.4, 1.7 Hz, 1H, H-11), 3.80 (s, 3H, H-20), 3.69 (s, 3H, H-19), 2.20 (d, *J* = 15.8 Hz, 1H, H-5), 2.08 (dd, *J* = 15.6, 1.7 Hz, 1H, H-5); ^{13}C NMR (125 MHz, DMSO) δ 167.1 (C, C-1), 165.0 (C, C-3), 153.5 (C, C-16), 148.3 (C, C-14), 136.3 (C, C-15), 131.8 (CH, C-9), 127.0 (CH, C-8), 123.5 (CH, C-18), 116.8 (C, C-13), 103.8 (CH, C-17), 86.0 (CH, C-11), 70.6 (C, C-4), 70.6 (C, C-6), 67.7 (CH, C-7), 64.7 (CH, C-10), 60.8 (CH_3, C-19), 59.4 (CH, C-2), 56.2 (CH_3, C-20), 45.4 (CH, C-12), 33.6 (CH_2, C-5).

Outovirin B (4)[3]**:** orange needles; HRESIMS *m/z* 513.0994 $[M+H]^+$ (calcd for $C_{21}H_{25}N_2O_9S_2$, 513.0996); $[\alpha]_D^{25}$-156.2 (c 0.2， MeOH); ECD (0.59 mM, MeOH) λ_{max} ($\Delta\varepsilon$) 216 (-30.29), 263(-8.50), 301(+0.83); ^{1}H NMR (500 MHz, DMSO-d_6) δ 9.46 (s, 1H, 14-OH), 7.37 (d, *J* = 8.4 Hz, 1H, H-18), 6.57 (d, *J* = 8.6 Hz, 1H, H-17), 5.50 (d, *J* = 10.2 Hz, 1H, H-9), 5.44 (d, *J* = 10.3 Hz, 1H, H-8), 5.29 (s, 1H, 7-OH), 5.17 (s, 1H, 6-OH), 5.17(s, 1H, 10-OH), 4.61 (s, 1H, H-2), 4.61 (s, 1H, H-12), 4.24 (s , 1H, H-10), 4.19 (s, 1H, H-7), 3.95 (d, *J* = 7.0 Hz, 1H, H-11), 3.81 (s, 3H, H-20), 3.70 (s, 3H, H-19), 3.01 (s, 3H, H-21), 2.20 (d, *J* = 15.7 Hz, 1H, H-5*a*), 2.00 (d, *J* = 15.8 Hz, 1H, H-5β). ^{13}C NMR (125 MHz, DMSO) δ 166.1 (C, C-1), 164.6 (C, C-3), 153.4 (C, C-16), 148.2 (C, C-14), 136.4 (C, C-15), 130.2 (CH, C-8), 129.2 (CH, C-9), 123.4 (CH, C-18), 117.0 (C, C-13), 103.8 (CH, C-17), 85.9 (CH, C-11), 74.6 (CH, C-7), 71.4 (C, C-6), 70.0 (C, C-4), 66.0 (CH, C-2), 65.1 (CH, C-10), 60.8 (CH_3, C-19), 56.2 (CH_3, C-20), 41.6 (CH, C-12), 33.1 (CH_3, C-21), 32.7 (CH_2, C-5).

Pretrichodermamide E ***(5)***[4]: yellow powder; $[\alpha]_D^{25}$-85.7 (c 0.1， MeOH); ECD (0.39 mM, MeOH) λ_{max} ($\Delta\varepsilon$) 202(+16.00), 217(-15.56), 258(-5.29), 299(+0.45); ^{1}H NMR (500 MHz, DMSO-d_6) δ 9.51 (s, 1H, 14-OH), 7.35 (d, *J* = 8.8 Hz, 1H, H-18), 6.58 (d, *J* = 8.9 Hz, 1H, H-17), 5.57 (dd, *J* = 10.2, 1.9 Hz, 1H, H-7), 5.46 (dd, *J* = 10.2, 1.9 Hz, 1H, H-8), 5.36 (s, 1H, 6-OH), 5.10 (d, *J* = 6.9

Hz, 1H, 9-OH), 4.75 (d, *J* = 5.6 Hz, 1H, H-12), 4.61 (s, 1H, H-2), 3.98 (t, *J* = 6.3 Hz, 1H, H-9), 3.85 (d, *J* = 10.5 Hz, 1H, H-11), 3.81 (s, 3H, H-20), 3.70 (s, 3H, H-19), 3.59 (m, 1H, H-10), 3.00 (s, 3H, H-21), 2.30 (d, *J* = 15.0 Hz, 1H, H- 5α), 2.24 (d, *J* = 18.0 Hz, 1H, H- 5β). ^{13}C NMR (125 MHz, DMSO) δ 166.1 (C, C-1), 165.1 (C, C-3), 153.6 (C, C-16), 148.2 (C, C-14), 136.4 (C, C-15), 132.5 (C, C-7), 130.3 (C, C-8), 123.3 (CH, C-18), 116.9 (C, C-13), 103.8 (CH, C-17), 83.9 (CH, C-11), 72.9 (CH, C-9), 71.5 (CH, C-10), 67.6 (C, C-4), 66.2 (C, C-6), 60.8 (CH_3, C-19), 56.2 (CH_3, C-20), 42.1 (CH, C-12), 39.1(CH_2, C-5), 33.2 (CH_3, C-21).

*Peniciadametizine A (**6**)*[5]: brown solid; HRESIMS *m/z* 507.1231 $[M+H]^+$ (calcd for $C_{23}H_{27}N_2O_7S_2$, 507.1254); $[\alpha]_D^{25}$ -43.4 (c 0.1, MeOH); ECD (0.59 mM, MeOH) λ_{max} ($\Delta\varepsilon$) 207 (+43.11), 222 (-0.30), 237 (+10.00), -270 (-19.19), 320 (+0.15); ^{1}H NMR (500 MHz, DMSO-d_6) δ 6.96 (d, *J* = 8.4 Hz, 1H, H-17), 6.78 (d, *J* = 8.4 Hz, 1H, H-18), 5.84 (d, *J* = 2.3 Hz, 1H, H-7), 5.79 (dd, *J* = 9.9, 2.7 Hz, 1H, H-8), 5.71 (d, *J* = 9.8 Hz, 1H, H-9), 5.31 (d, *J* = 5.3 Hz, 1H, 10-OH), 4.98 (s, 1H, H-12), 4.94 (d, *J* = 13.4 Hz, 1H, H-11), 4.56 (dd, *J* = 15.0, 4.5 Hz, 1H, H-10), 3.83 (s, 3H, H-20), 3.75 (s, 3H, H-19), 3.60 (d, *J* = 13.9 Hz, 1H, H-5α), 3.00 (d, *J* = 14.4 Hz, 1H, H-5β), 2.80 (s, 3H), 2.70 (s, 1H, H-21), 2.38 (s, 3H,), 2.26 (s, 1H, H-22), 1.80 (s, 3H, H-23). ^{13}C NMR (125 MHz, DMSO) δ 162.3 (C, C-1), 159.3 (C, C-3), 153.7 (C,C-16), 150.3 (C, C-14), 132.8 (C, C-15), 130.5 (CH, C-9), 130.3 (C,C-6), 123.0 (CH, C-8), 122.8 (CH, C-7), 119.8 (CH, C-18), 118.9 (C, C-13), 107.6 (CH, C-17), 101.4 (C, C-2), 92.0 (CH, C-11), 70.9 (C,C-4), 70.1 (CH, C-10), 60.8 (CH_3, C-19), 56.8 (CH_3,C-20), 54.0 (CH, C-12), 37.4 (CH_2, C-5), 28.8 (CH_3, C-21), 13.8 (CH_3, C-22), 13.6 (CH_3, C-23).

(1) Liu, Y.; Li, X. M.; Meng, L. H.; Jiang, W. L.; Xu, G. M.; Huang, C. G.; Wang, B. G. Bisthiodiketopiperazines and Acorane Sesquiterpenes Produced by the Marine-Derived Fungus *Penicillium Adametzioides* AS-53 on Different Culture Media. *J. Nat. Prod.* **2015**, *78* (6), 1294–1299.

(2) Yamazaki, H.; Takahashi, O.; Murakami, K.; Namikoshi, M. Induced Production of a New Unprecedented Epitrithiodiketopiperazine, Chlorotrithiobrevamide, by a Culture of the Marine-Derived *Trichoderma* Cf. *Brevicompactum* with Dimethyl Sulfoxide. *Tetrahedron Letters* **2015**, *56* (45), 6262–6265.

(3) Kajula, M.; Ward, J. M.; Turpeinen, A.; Tejesvi, M. V.; Hokkanen, J.; Tolonen, A.; Häkkänen, H.; Picart, P.; Ihalainen, J.; Sahl, H.-G.; Pirttilä, A. M.; Mattila, S. Bridged Epipolythiodiketopiperazines from *Penicillium Raciborskii*, an Endophytic Fungus of *Rhododendron Tomentosum* Harmaja. *J. Nat. Prod.* **2016**, *79* (4), 685–690.

(4) Yurchenko, A. N.; Smetanina, O. F.; Ivanets, E. V.; Kalinovsky, A. I.; Dyshlovoy, S. A. Pretrichodermamides D–F from a Marine Algicolous Fungus *Penicillium* Sp. KMM 4672. *Marine Drugs* **2016**, *14* (7), 122.

(5) Liu, Y.; Mándi, A.; Li, X. M.; Meng, L. H.; Kurtán, T.; Wang, B.-G. Peniciadametizine A, a Dithiodiketopiperazine with a Unique Spiro [Furan-2,7'-Pyrazino[1,2-b][1,2]Oxazine] Skeleton, and a Related Analogue, Peniciadametizine B, from the Marine Sponge-Derived Fungus *Penicillium Adametzioides*. *Marine Drugs* **2015**, *13* (6), 3640–3652.

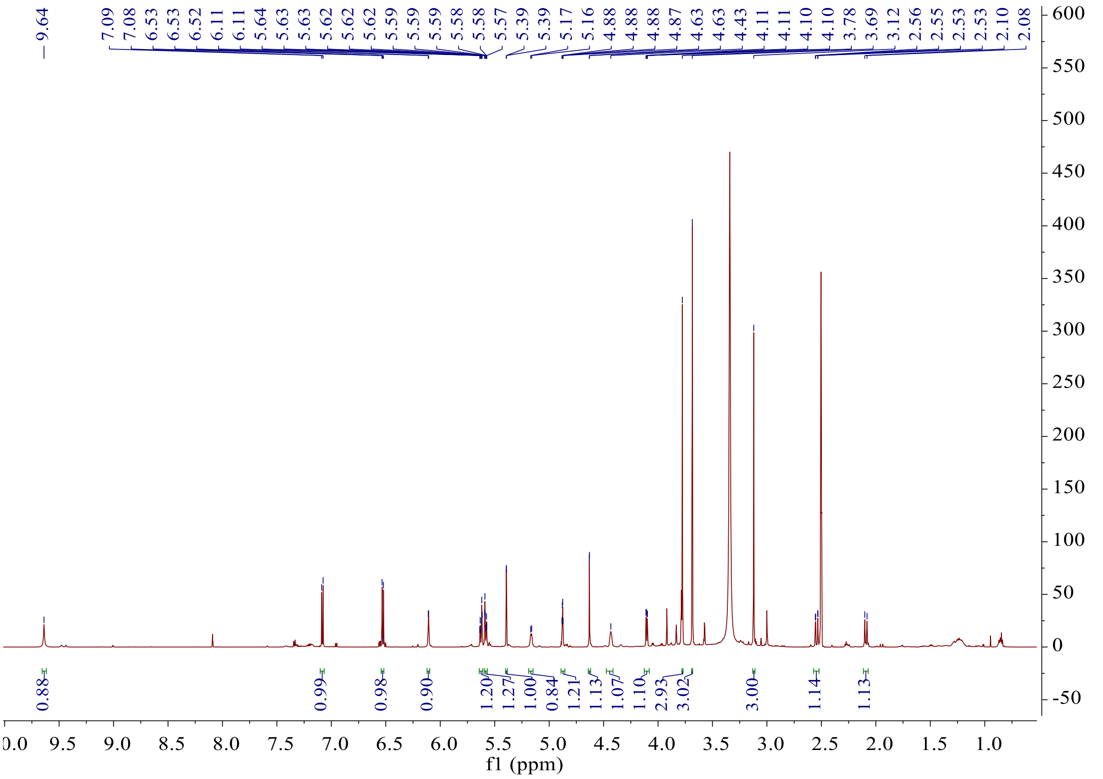

Fig. S1 1H NMR spectra of 1 in DMSO-d_6.

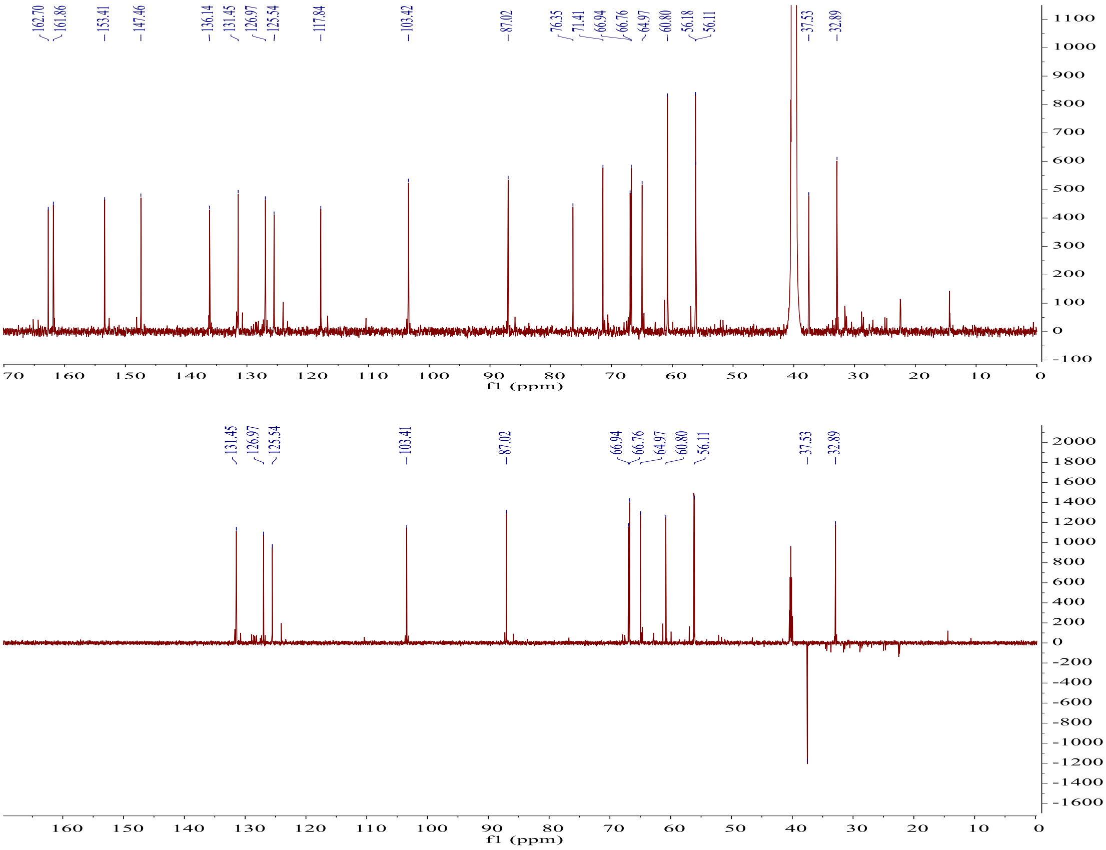

Fig. S2 ^{13}C and DEPT NMR spectra of **1** in DMSO-d_6.

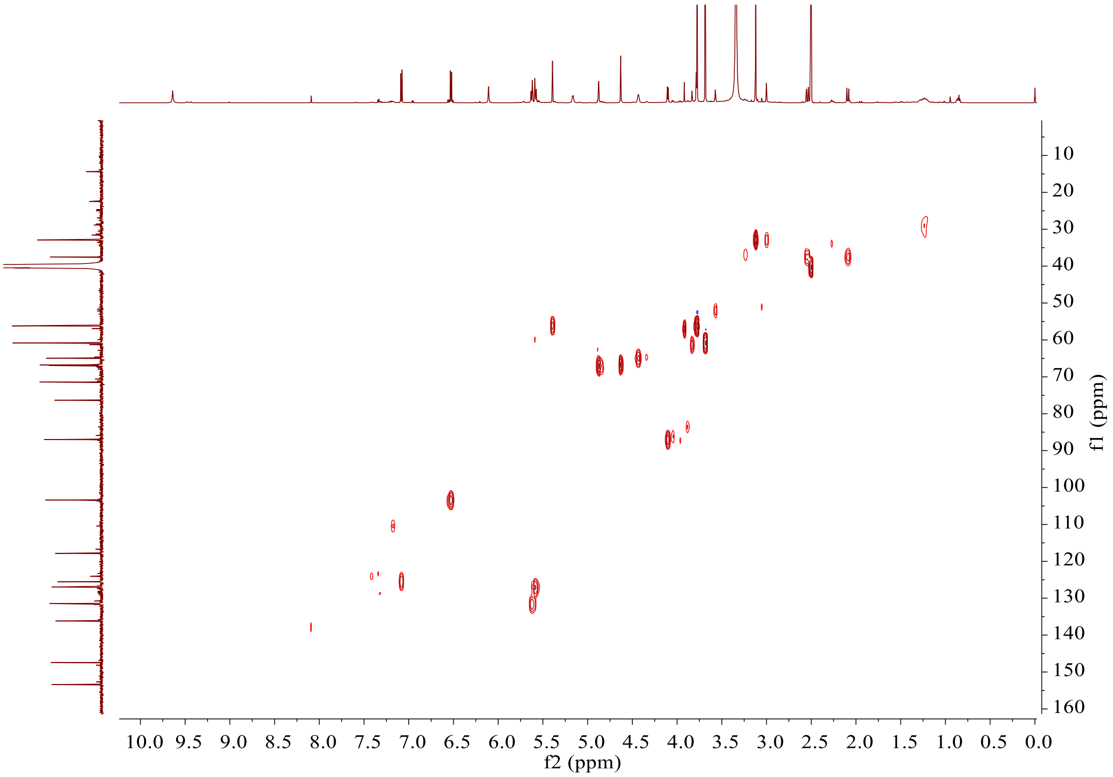

Fig. S3 HSQC spectrum of **1** in DMSO-d_6.

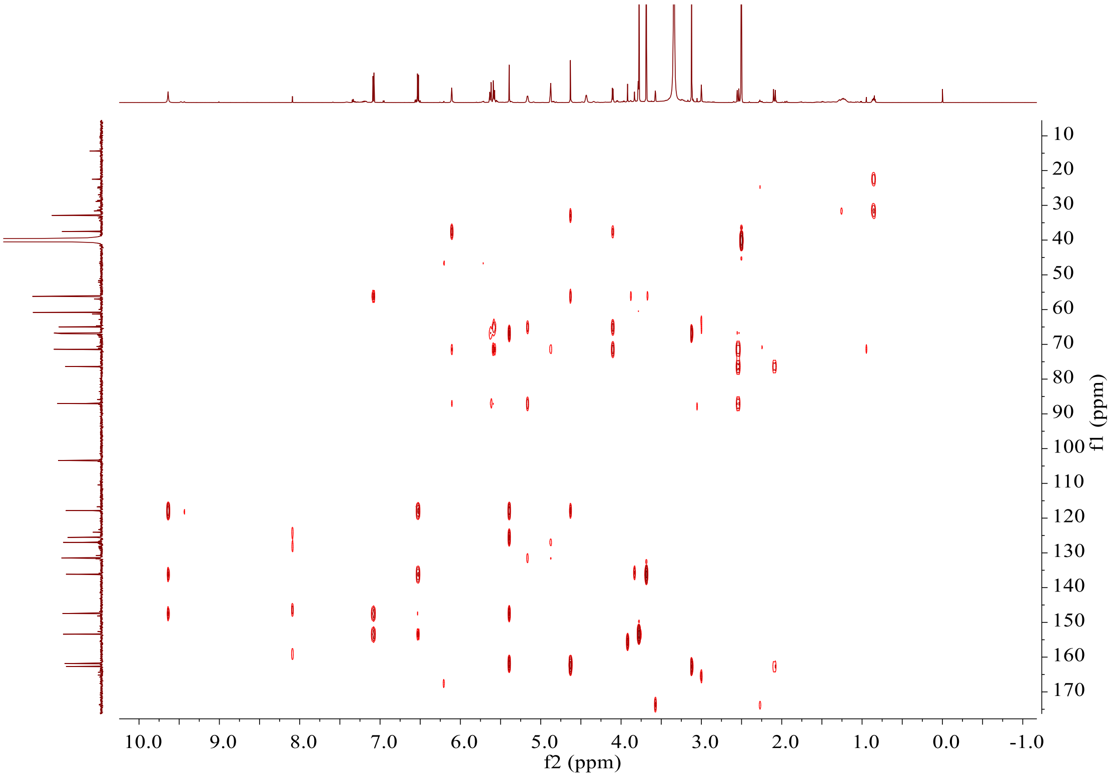

Fig. S4 HMBC spectrum of **1** in DMSO-d_6.

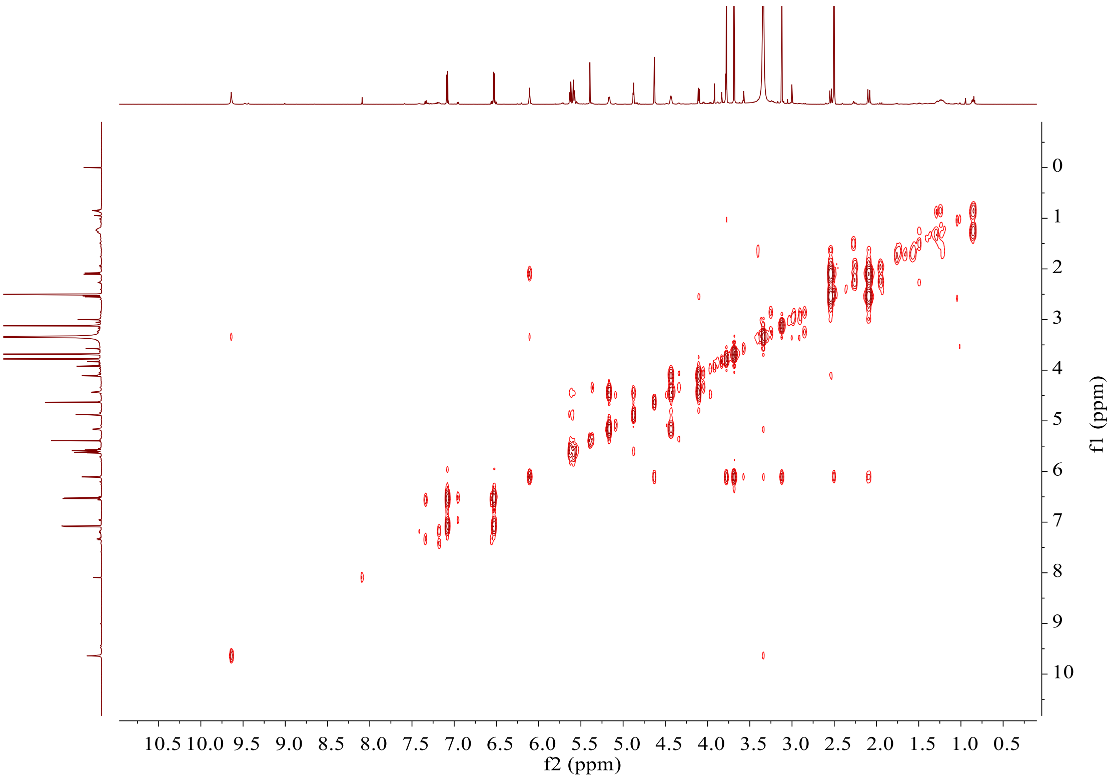

Fig. S5 COSY spectrum of **1** in DMSO-d_6.

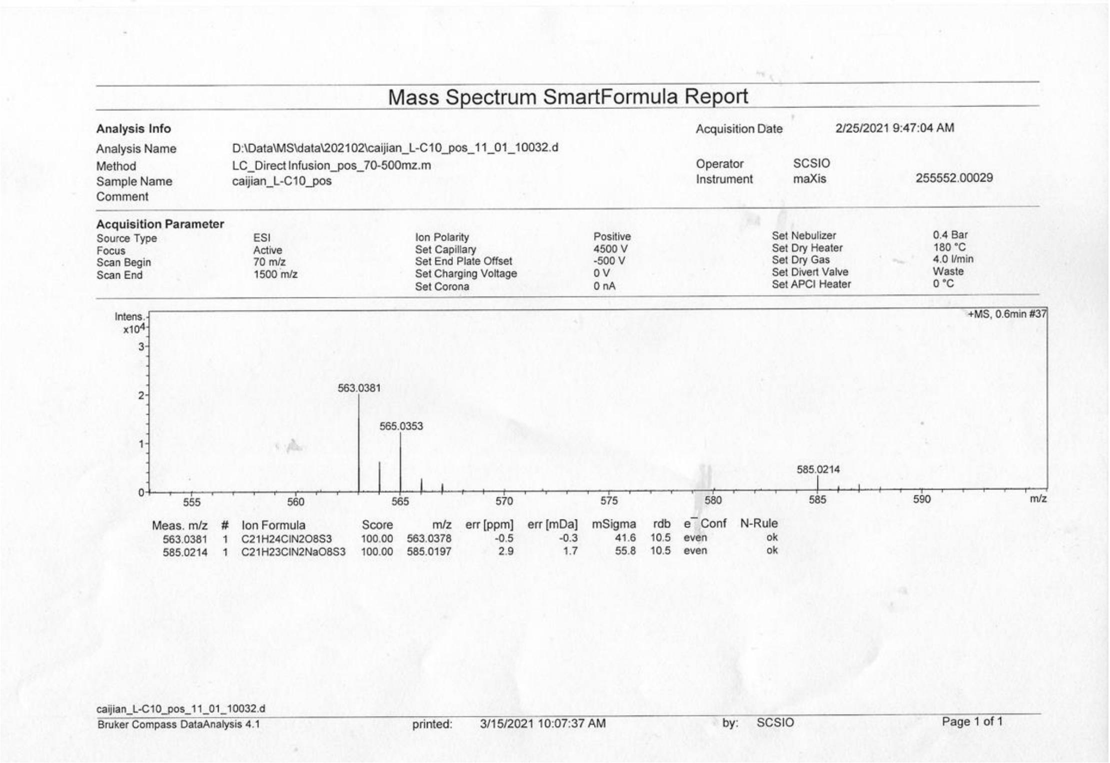

Mass Spectrum SmartFormula Report

**Analysis Info**		Acquisition Date	2/25/2021 9:47:04 AM
Analysis Name	D:\Data\MS\data\202102\caijian_L-C10_pos_11_01_10032.d		
Method	LC_Direct Infusion_pos_70-500mz.m	Operator	SCSIO
Sample Name	caijian_L-C10_pos	Instrument	maXis 255552.00029
Comment			

Acquisition Parameter

Source Type	ESI	Ion Polarity	Positive	Set Nebulizer	0.4 Bar
Focus	Active	Set Capillary	4500 V	Set Dry Heater	180 °C
Scan Begin	70 m/z	Set End Plate Offset	-500 V	Set Dry Gas	4.0 l/min
Scan End	1500 m/z	Set Charging Voltage	0 V	Set Divert Valve	Waste
		Set Corona	0 nA	Set APCI Heater	0 °C

Meas. m/z	#	Ion Formula	Score	m/z	err [ppm]	err [mDa]	mSigma	rdb	e⁻ Conf	N-Rule
563.0381	1	C21H24ClN2O8S3	100.00	563.0378	-0.5	-0.3	41.6	10.5	even	ok
585.0214	1	C21H23ClN2NaO8S3	100.00	585.0197	2.9	1.7	55.8	10.5	even	ok

caijian_L-C10_pos_11_01_10032.d
Bruker Compass DataAnalysis 4.1 printed: 3/15/2021 10:07:37 AM by: SCSIO Page 1 of 1

Fig. S6 HRESIMS spectrum of compound **1**.

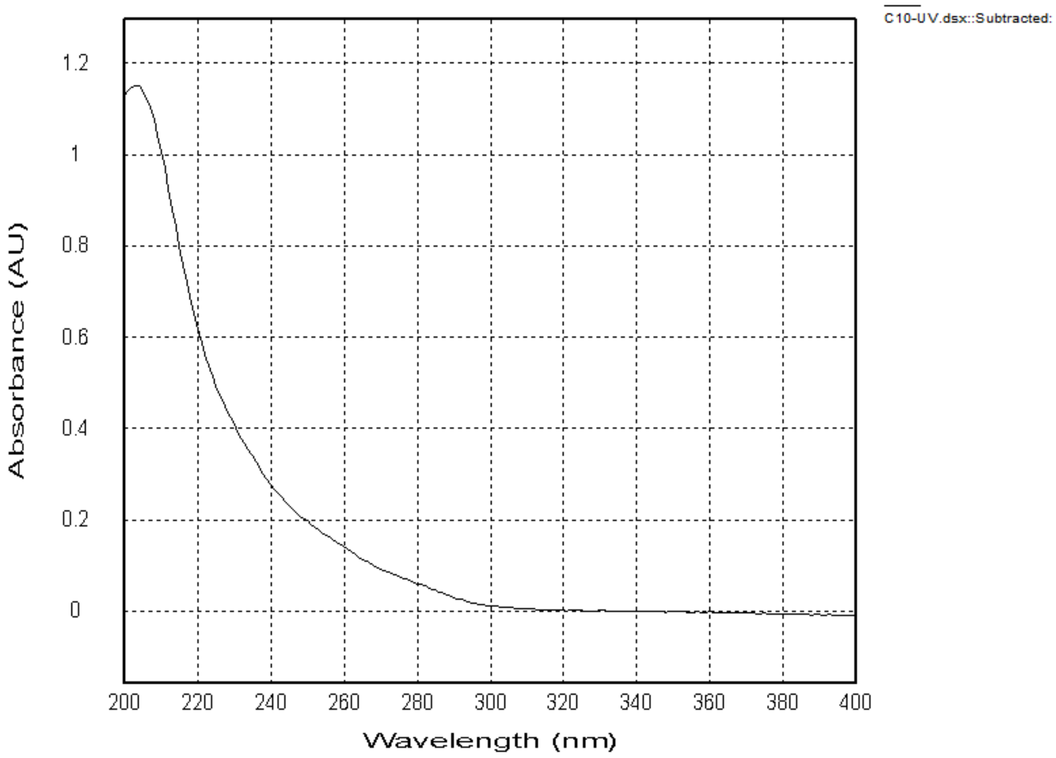

Fig. S7 The UV spectrum of **1**.

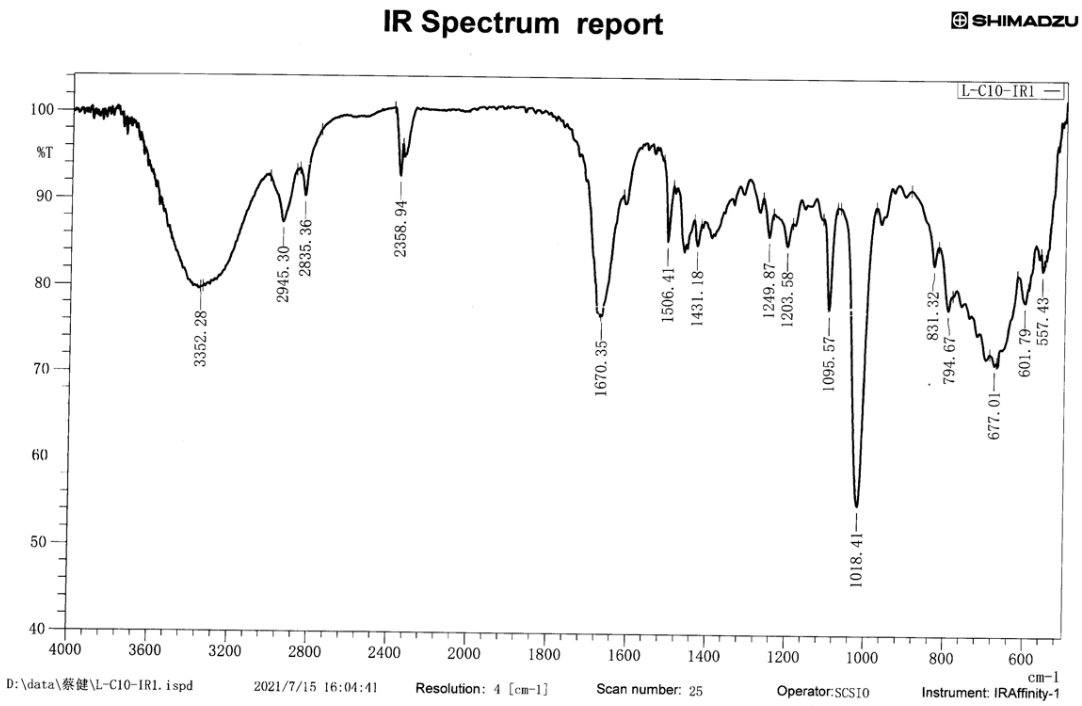

Fig. S8 The IR spectrum of **1**.

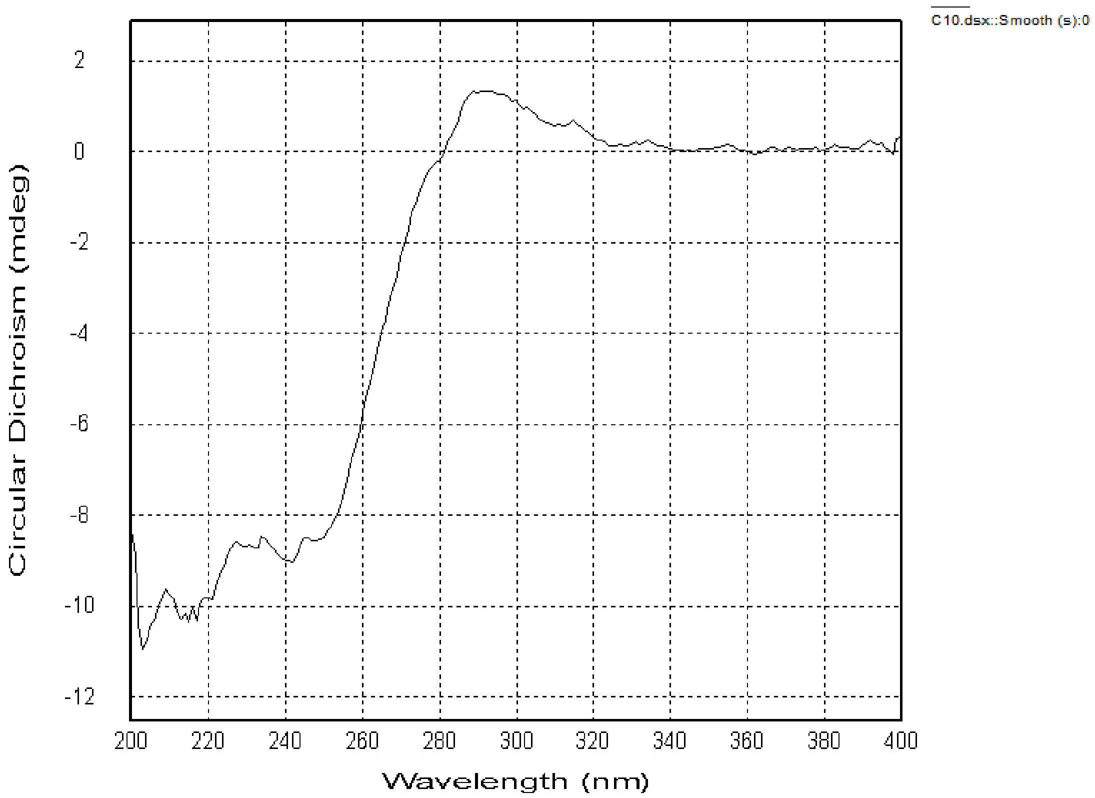

Fig. S9 The CD spectrum of **1** in MeOH.

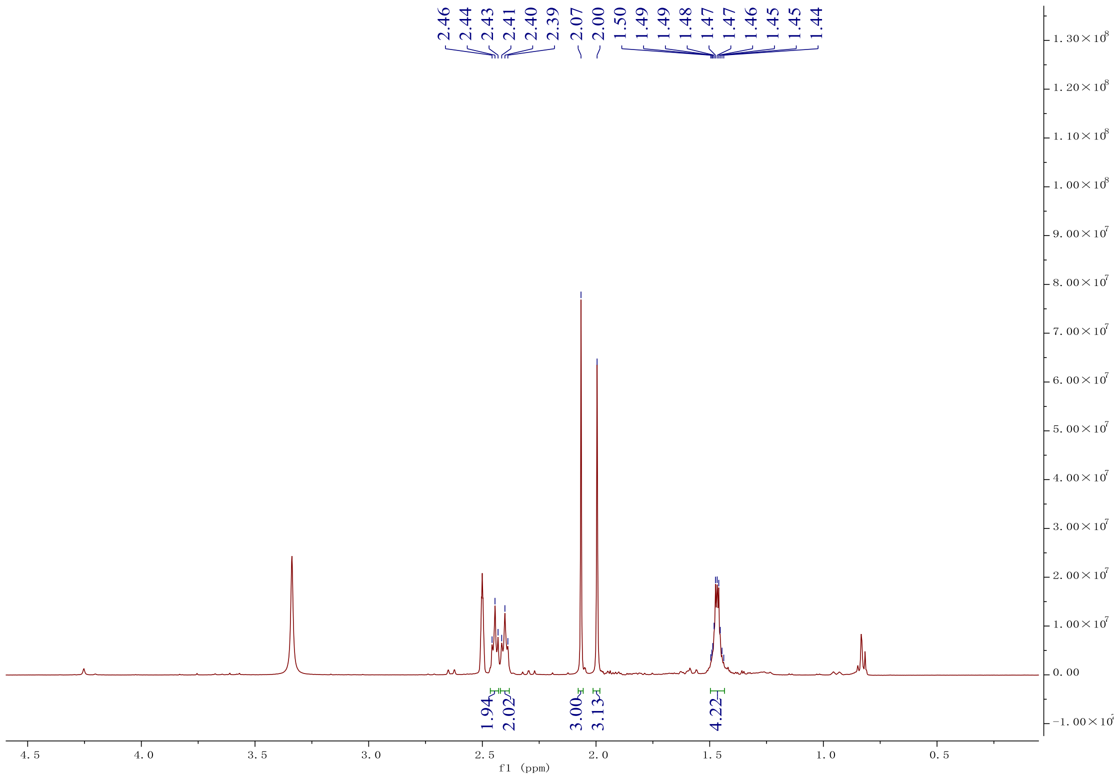

Fig. S10 ^{1}H NMR spectrum of **7** in DMSO-d_6

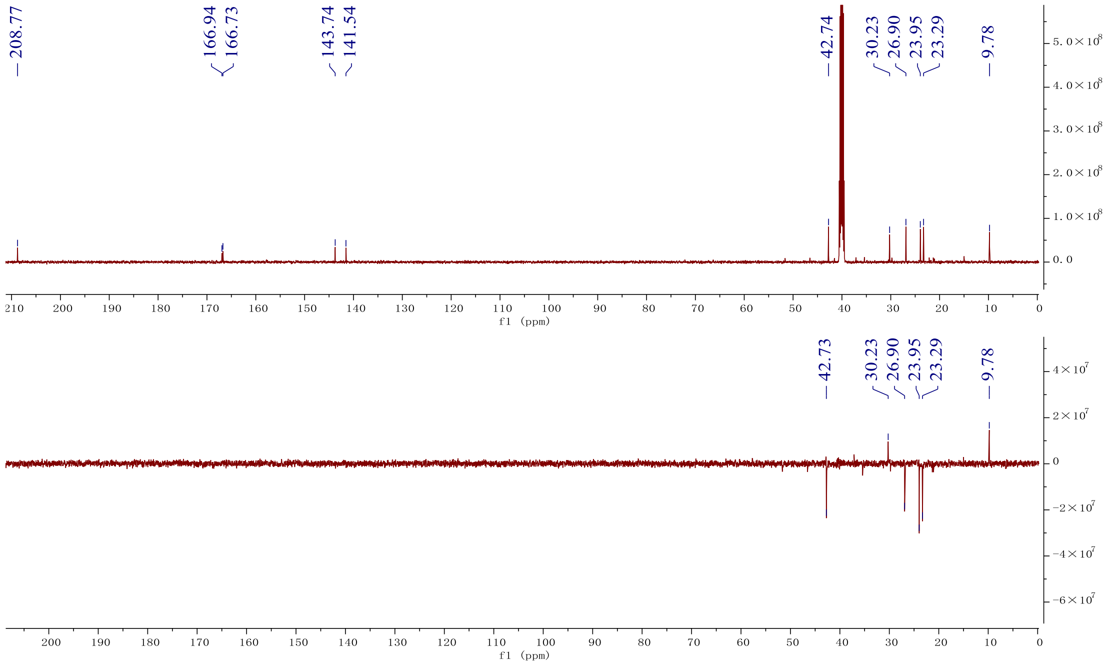

Fig. S11 ^{13}C and DEPT NMR spectra of **7** in DMSO-d_6

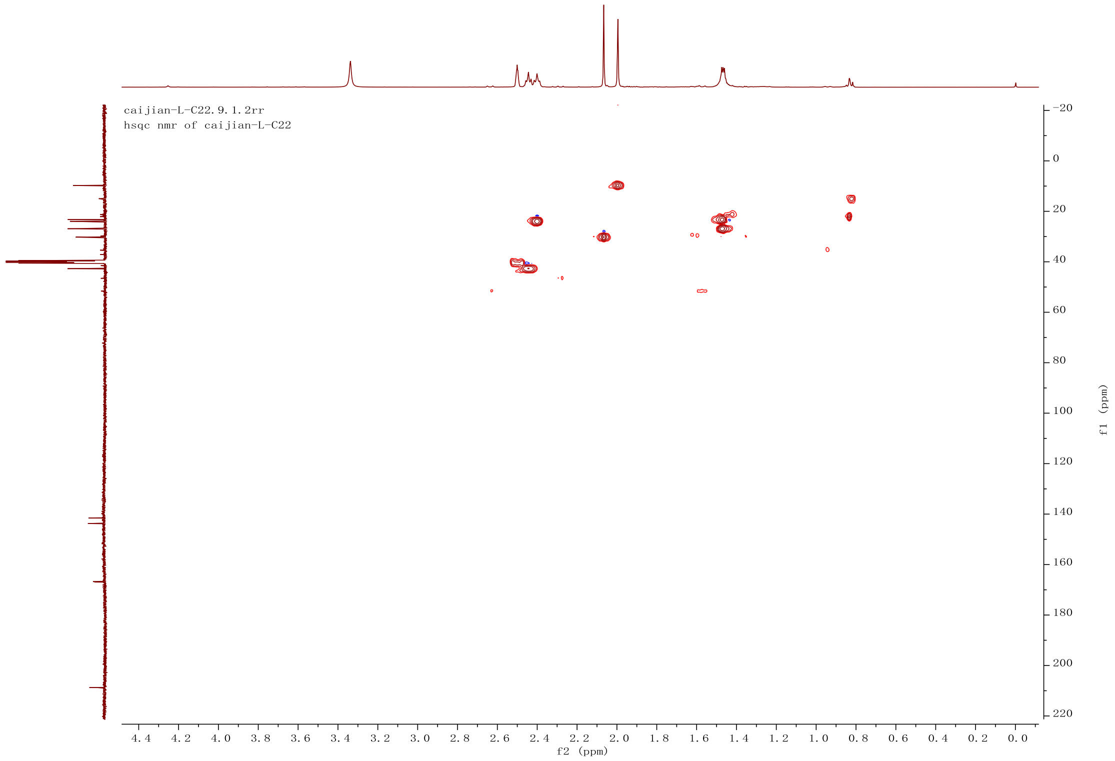

Fig. S12 HSQC spectrum of **7** in DMSO-d_6

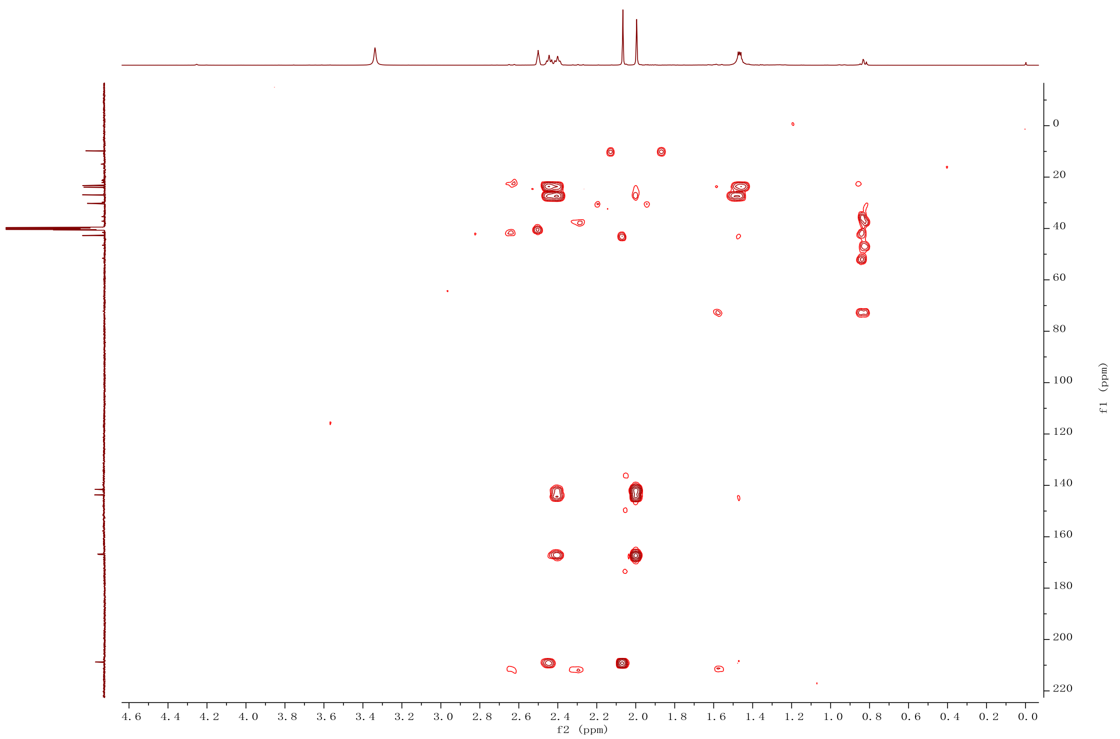

Fig. S13 HMBC spectrum of **7** in DMSO-d_6

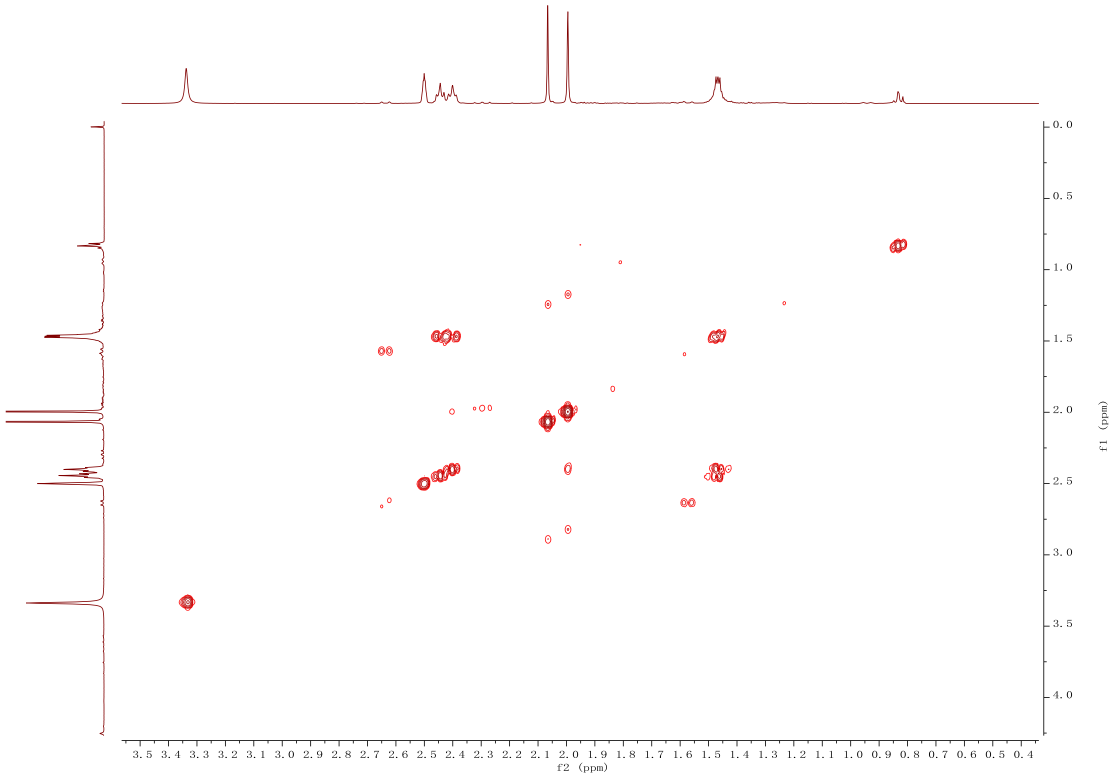

Fig. S14 COSY spectrum of **7** in DMSO-d_6

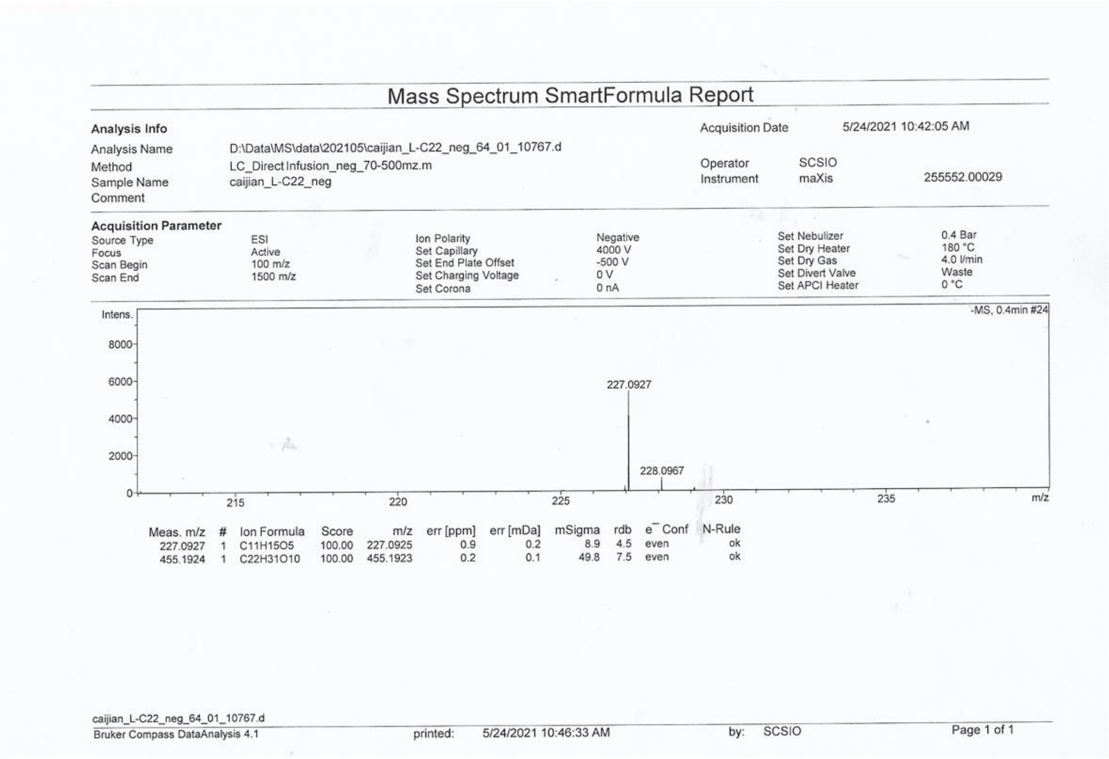
Mass Spectrum SmartFormula Report

Analysis Info		Acquisition Date	5/24/2021 10:42:05 AM
Analysis Name	D:\Data\MS\data\202105\caijian_L-C22_neg_64_01_10767.d		
Method	LC_Direct Infusion_neg_70-500mz.m	Operator	SCSIO
Sample Name	caijian_L-C22_neg	Instrument	maXis 255552.00029
Comment			

Acquisition Parameter

Source Type	ESI	Ion Polarity	Negative	Set Nebulizer	0.4 Bar		
Focus	Active	Set Capillary	4000 V	Set Dry Heater	180 °C		
Scan Begin	100 m/z	Set End Plate Offset	-500 V	Set Dry Gas	4.0 l/min		
Scan End	1500 m/z	Set Charging Voltage	0 V	Set Divert Valve	Waste		
		Set Corona	0 nA	Set APCI Heater	0 °C		

Meas. m/z	#	Ion Formula	Score	m/z	err [ppm]	err [mDa]	mSigma	rdb	e⁻ Conf	N-Rule
227.0927	1	C11H15O5	100.00	227.0925	0.9	0.2	8.9	4.5	even	ok
455.1924	1	C22H31O10	100.00	455.1923	0.2	0.1	49.8	7.5	even	ok

caijian_L-C22_neg_64_01_10767.d
Bruker Compass DataAnalysis 4.1 printed: 5/24/2021 10:46:33 AM by: SCSIO Page 1 of 1

Fig. S15 HRESIMS spectrum of compound **7**.

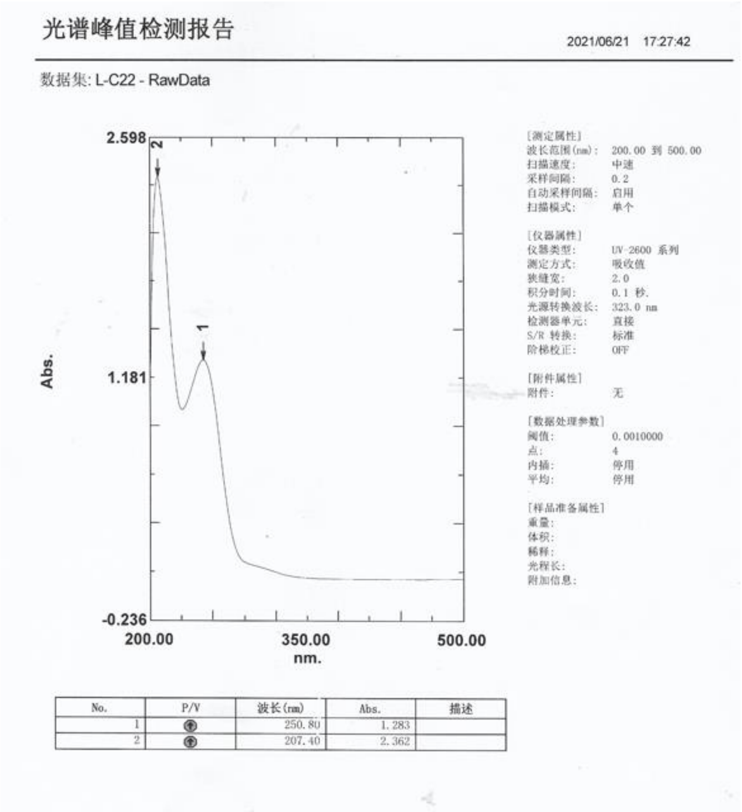
光谱峰值检测报告
2021/06/21 17:27:42
数据集: L-C22 - RawData

[测定属性]
波长范围(nm): 200.00 到 500.00
扫描速度: 中速
采样间隔: 0.2
自动采样间隔: 启用
扫描模式: 单个

[仪器属性]
仪器类型: UV-2600 系列
测定方式: 吸收值
狭缝宽: 2.0
积分时间: 0.1 秒.
光源转换波长: 323.0 nm
检测器单元: 直接
S/R 转换: 标准
阶梯校正: OFF

[附件属性]
附件: 无

[数据处理参数]
阈值: 0.0010000
点: 4
内插: 停用
平均: 停用

[样品准备属性]
重量:
体积:
稀释:
光程长:
附加信息:

No.	P/V	波长(nm)	Abs.	描述
1	⊕	250.80	1.283	
2	⊕	207.40	2.362	

Fig. S16 The UV spectrum of **7**.

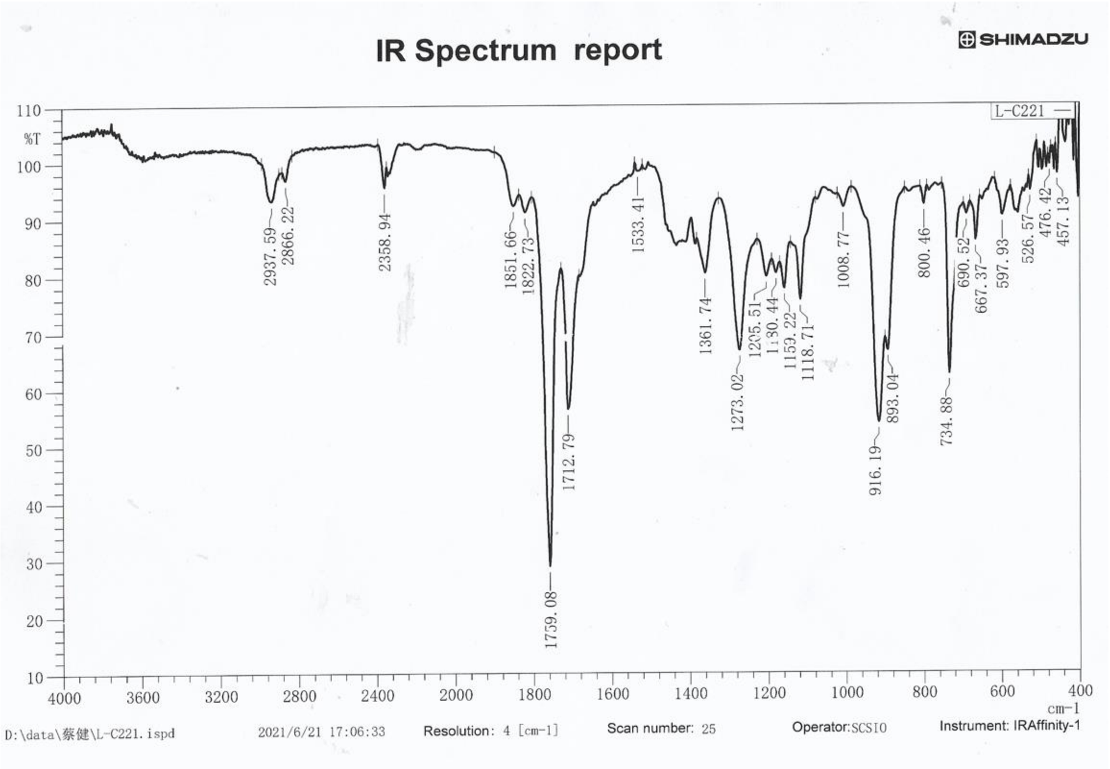

Fig. S17 The IR spectrum of **7**.

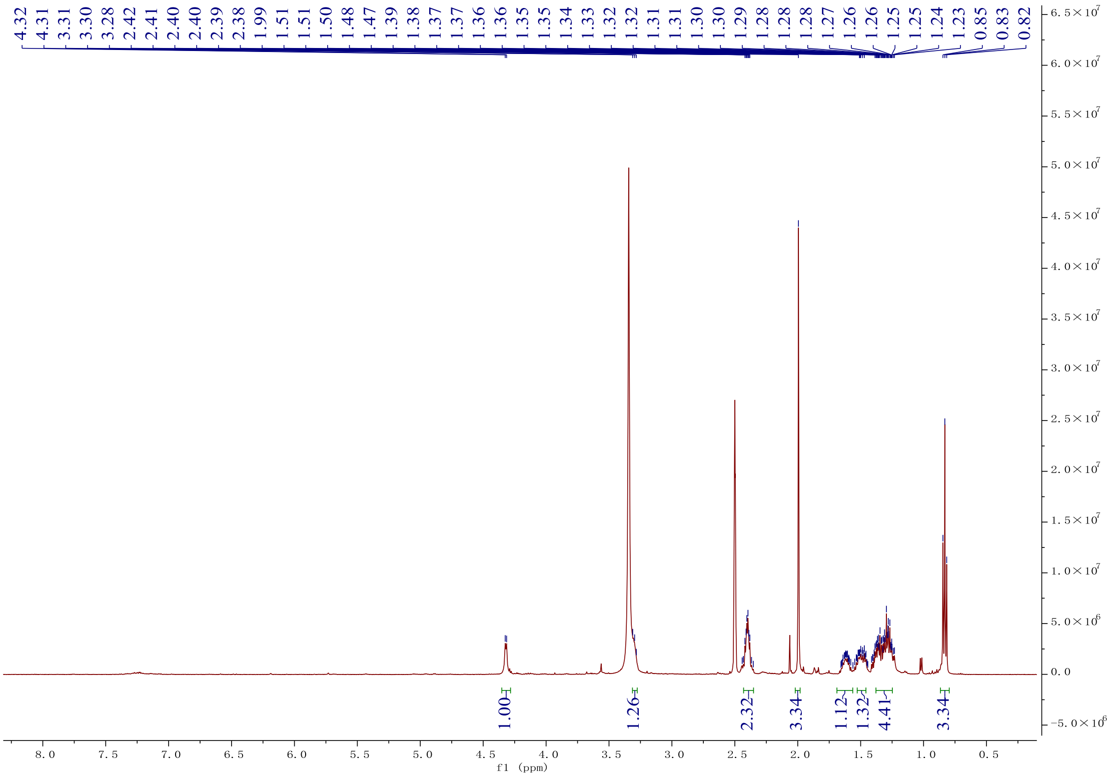

Fig. S18 ^{1}H NMR spectrum of **8** in DMSO-d_6

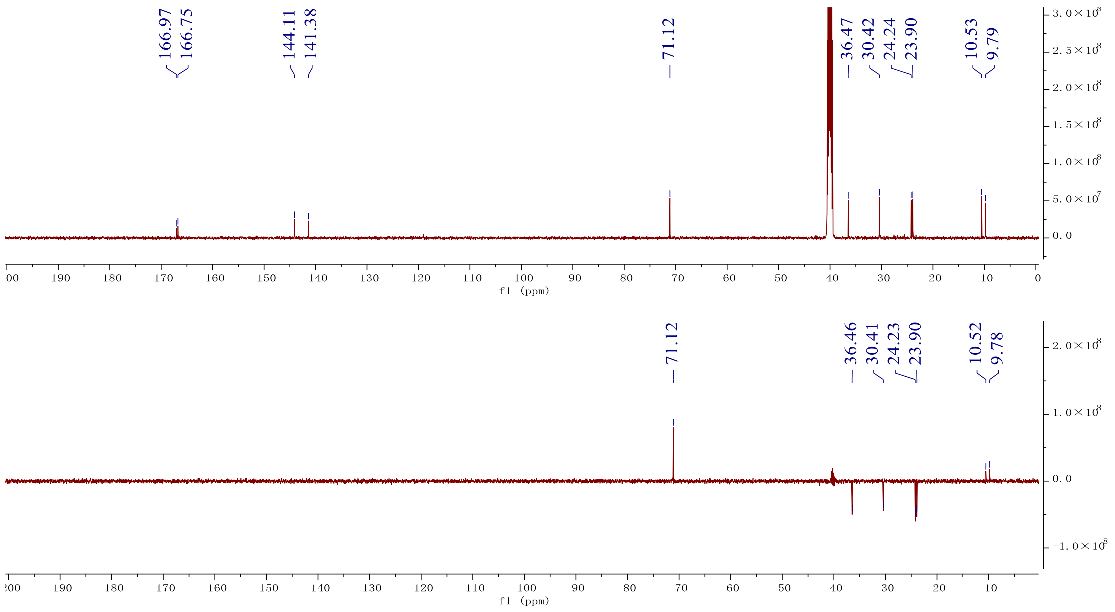

Fig. S19 ^{13}C and DEPT NMR spectra of **8** in DMSO-d_6

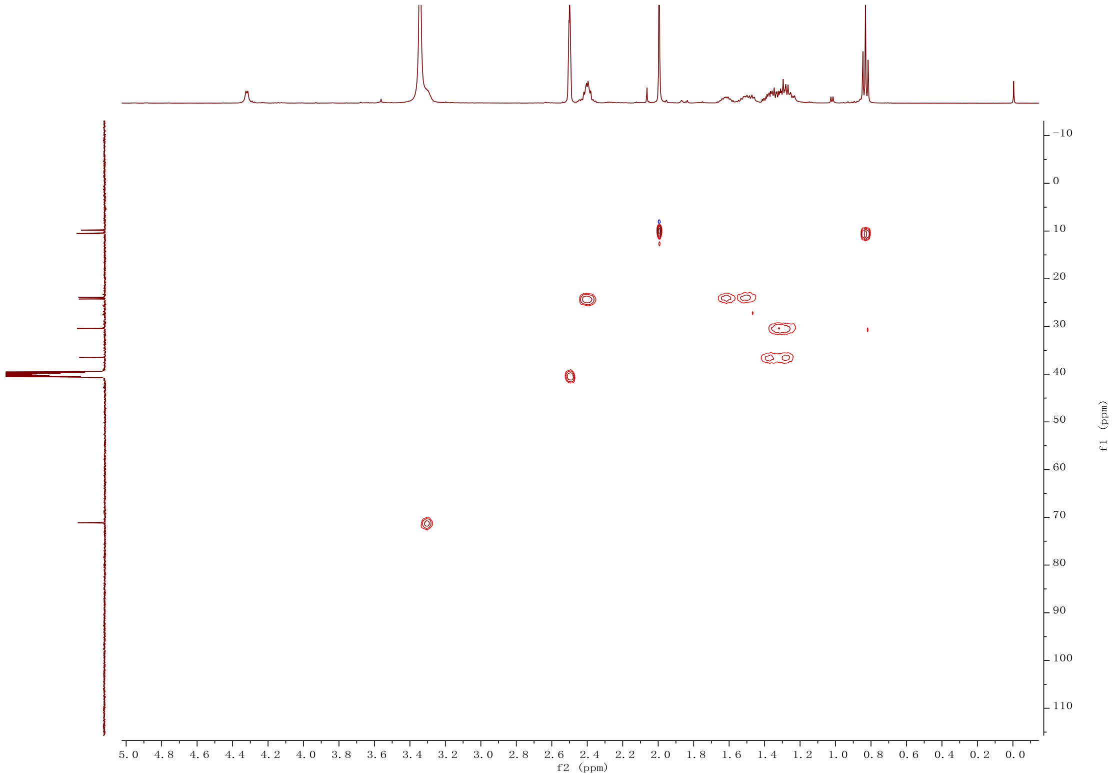

Fig. S20 HSQC spectrum of **8** in DMSO-d_6

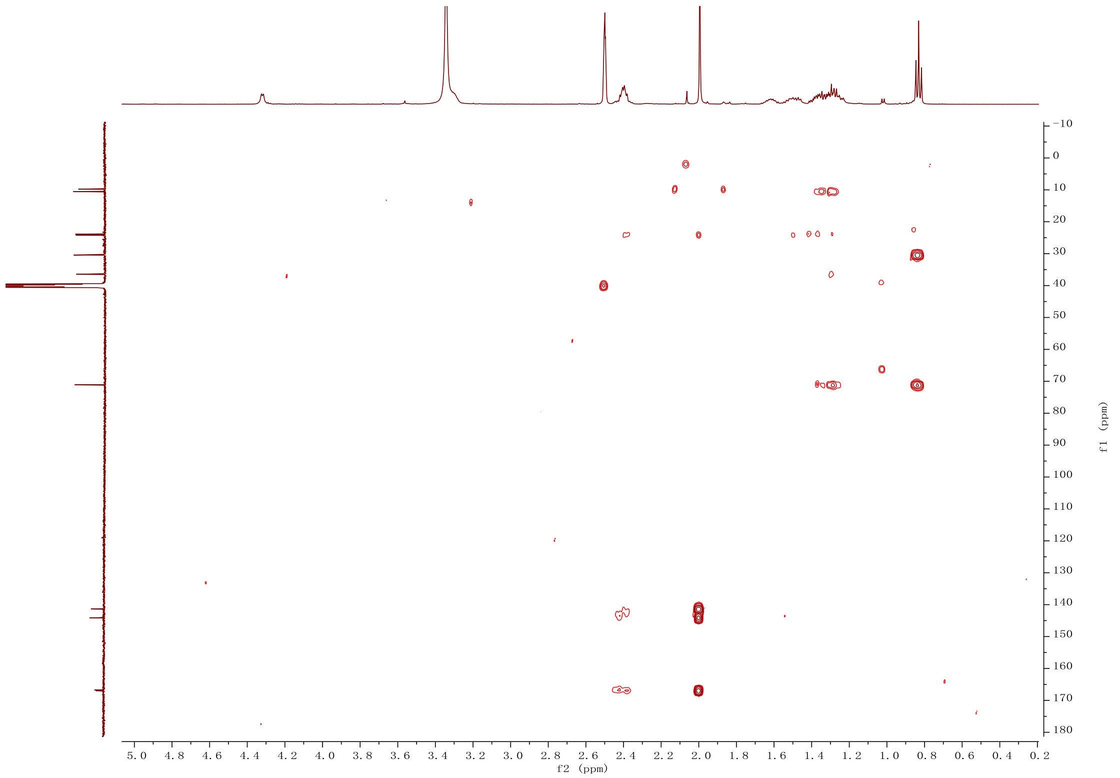

Fig. S21 HMBC spectrum of **8** in DMSO-d_6

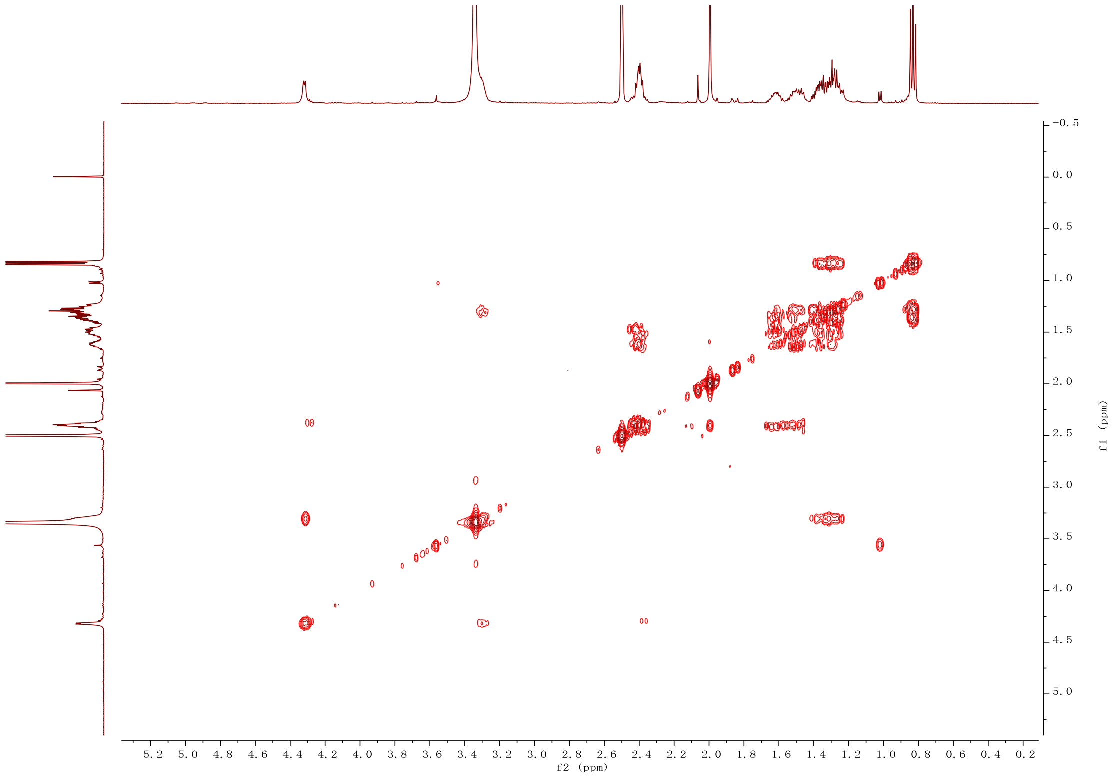

Fig. S22 COSY spectrum of **8** in DMSO-d_6

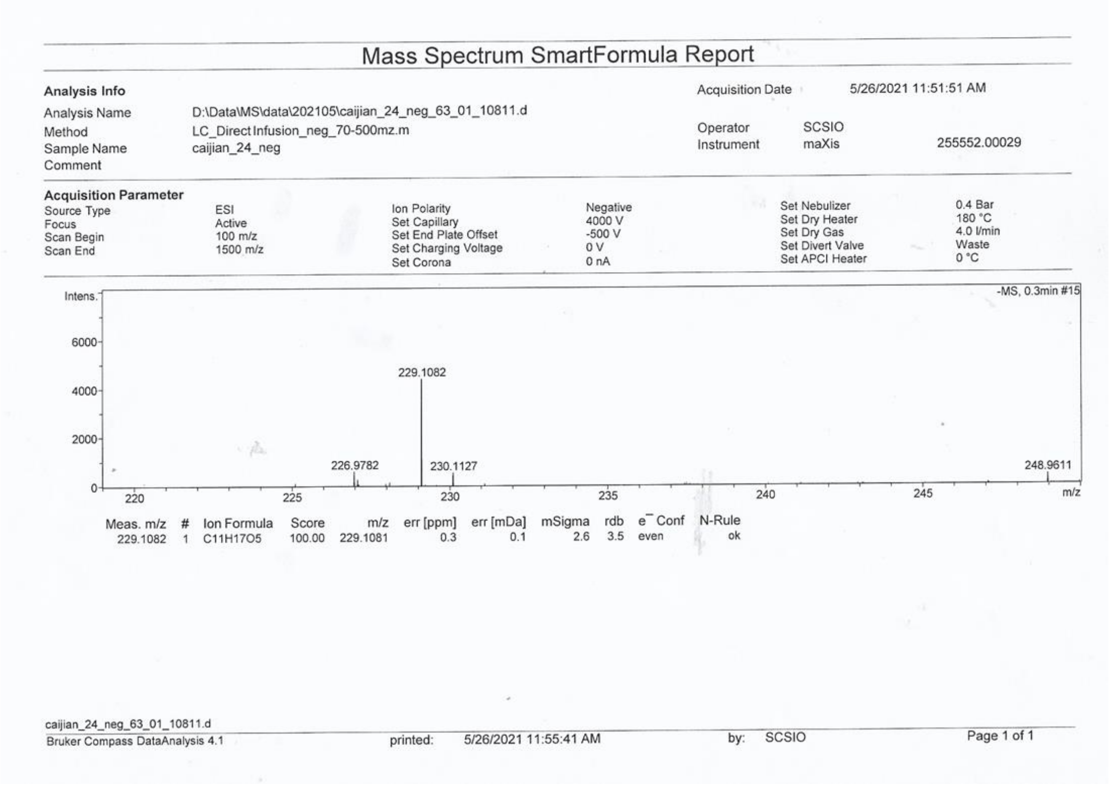

Fig. S23 HRESIMS spectrum of compound **8**.

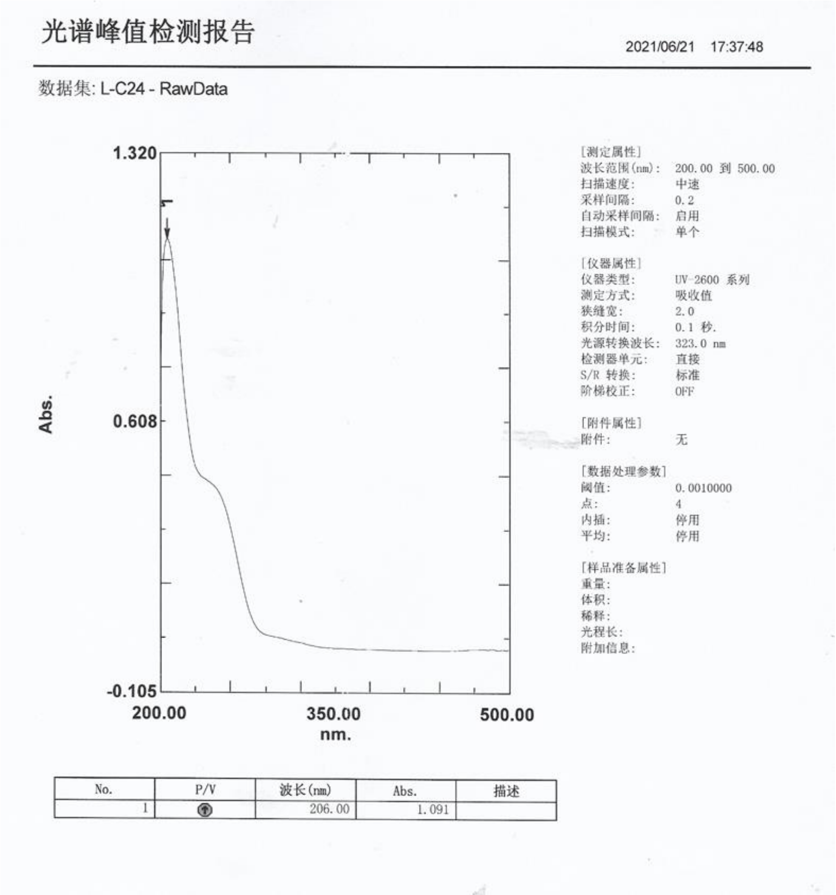

Fig. S24 The UV spectrum of **8**.

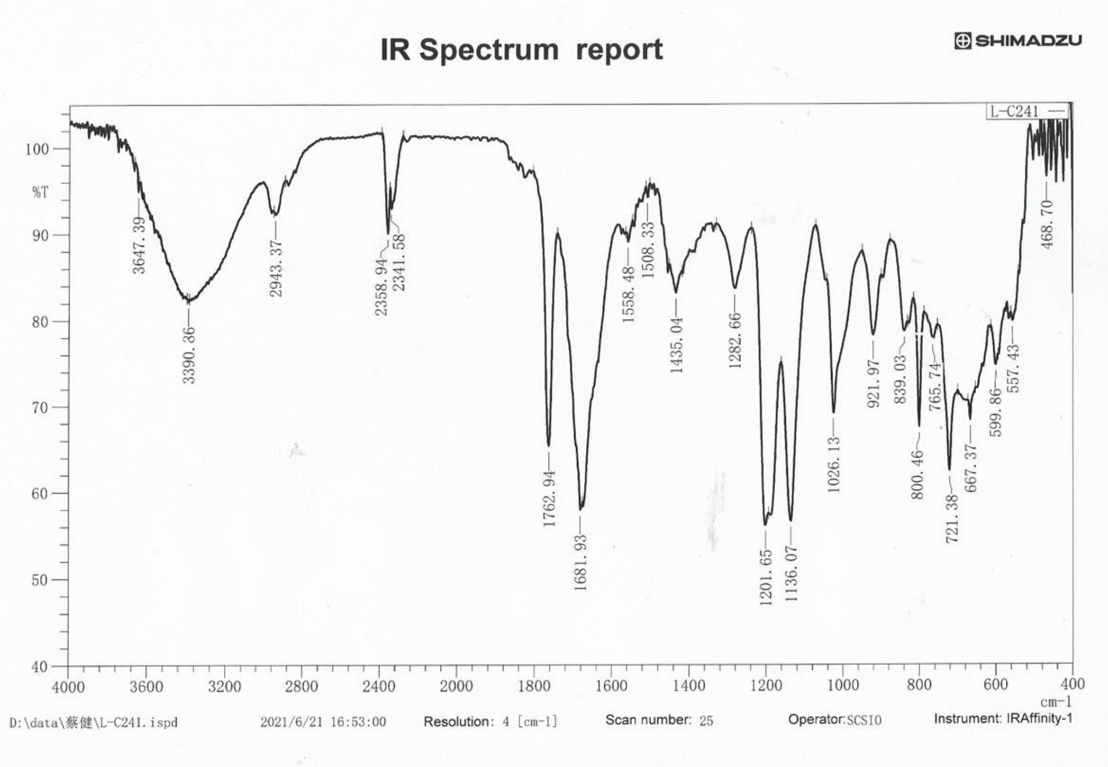

Fig. S25 The IR spectrum of **8**.

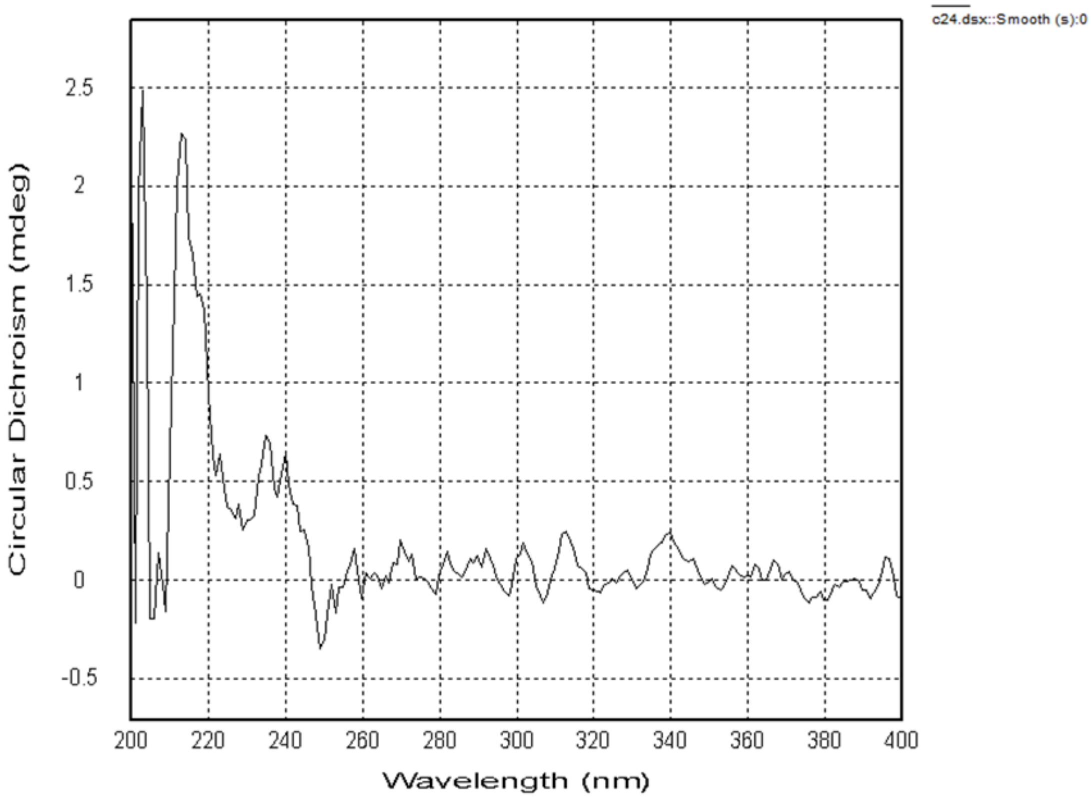

Fig. S26 The CD spectrum of **8** in MeOH.

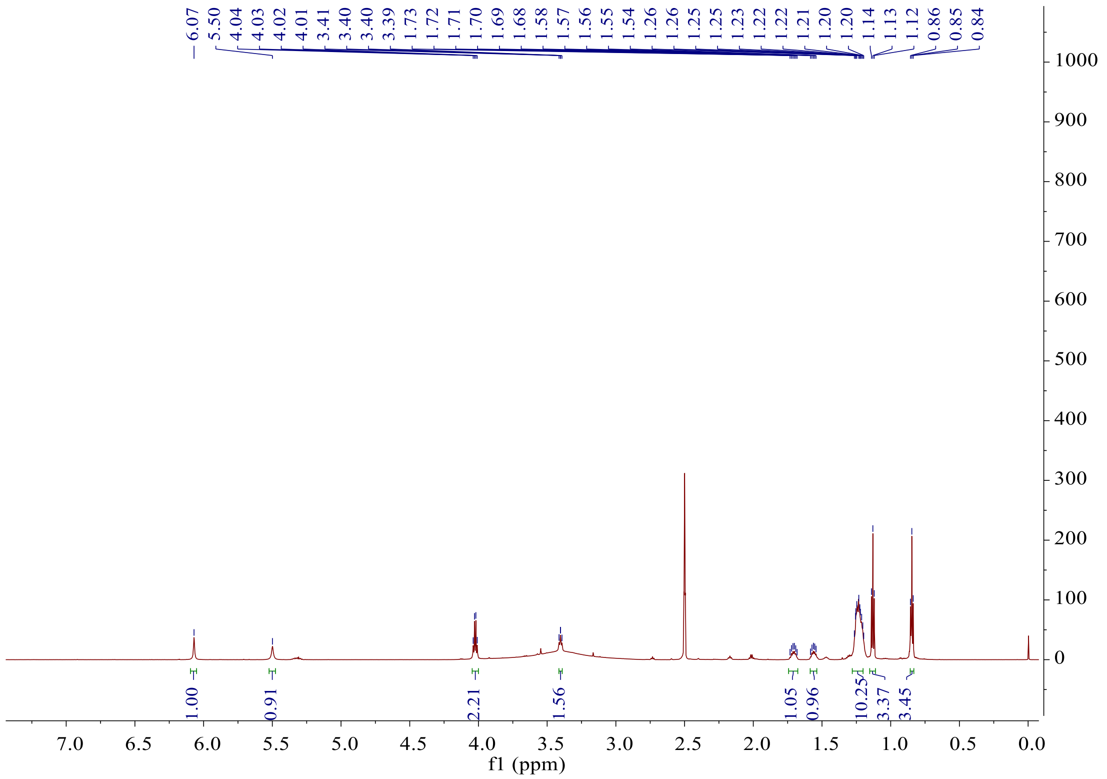

Fig. S27 ^{1}H NMR spectrum of **9** in DMSO-d_6

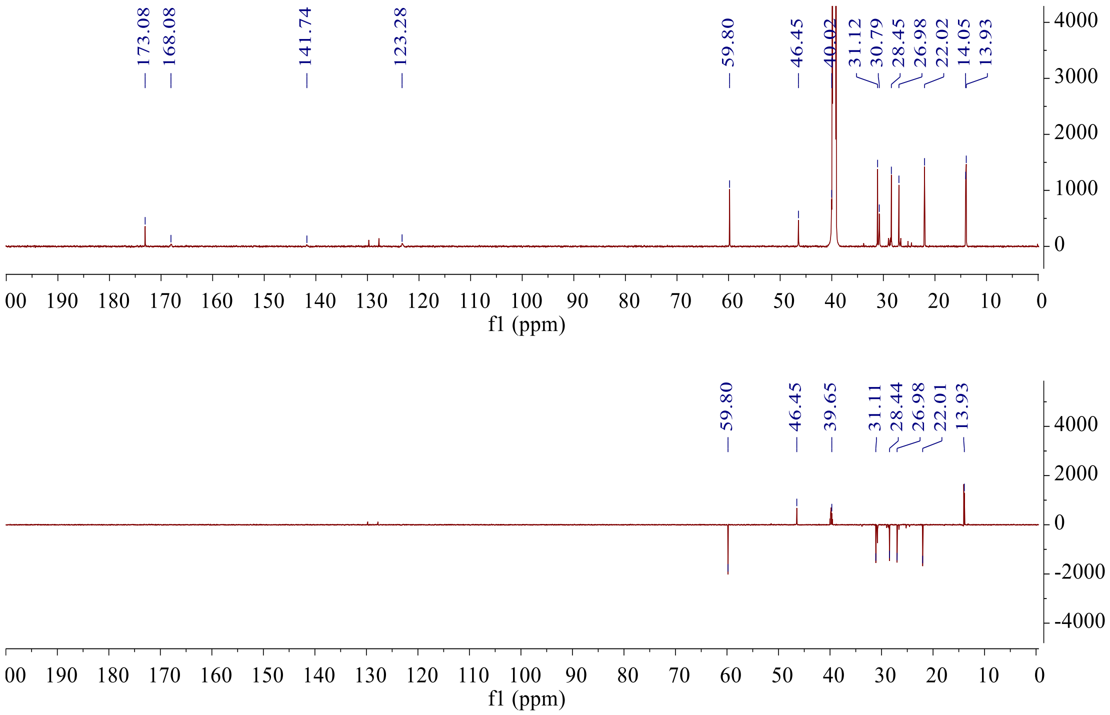

Fig. S28 ^{13}C and DEPT NMR spectra of **9** in DMSO-d_6.

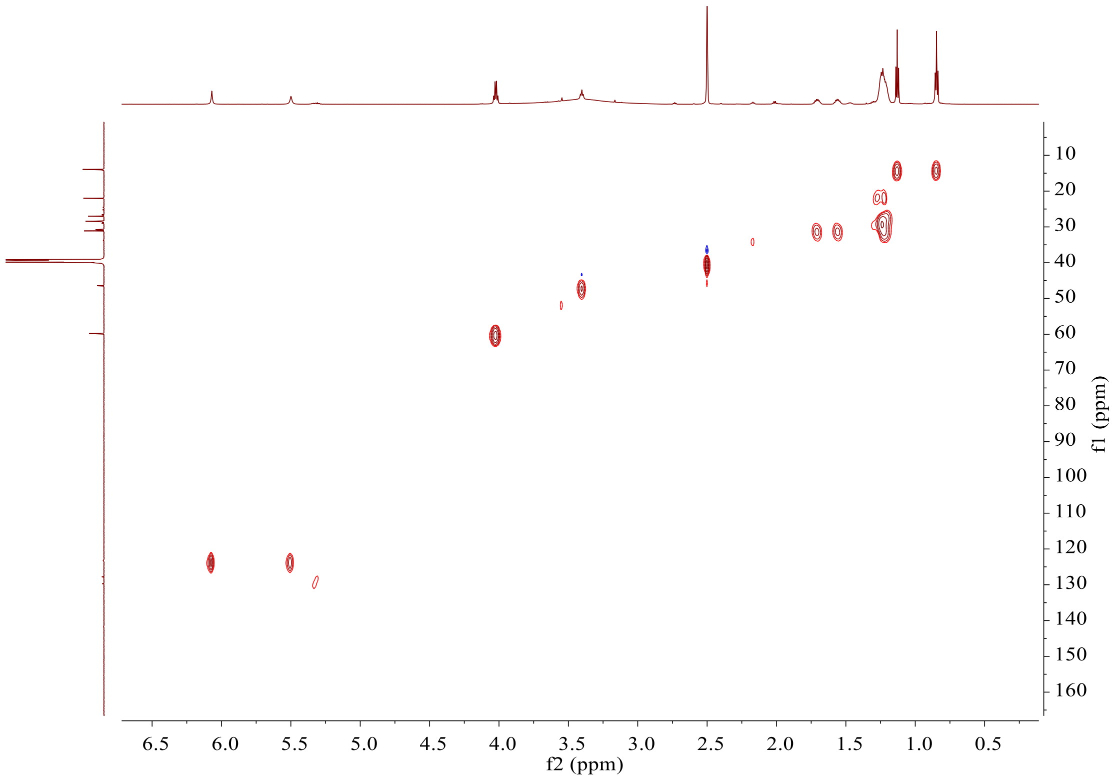

Fig. S29 HSQC spectrum of 9 in DMSO-d6.

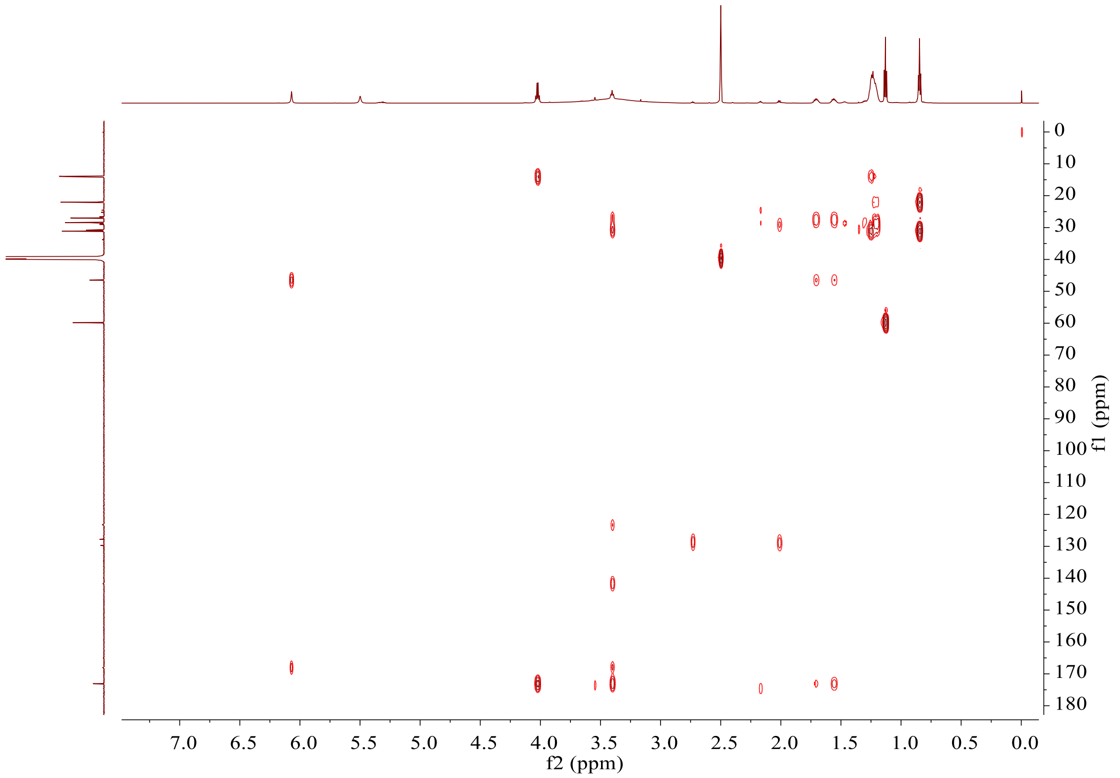

Fig. S30 HMBC spectrum of **9** in DMSO-d_6.

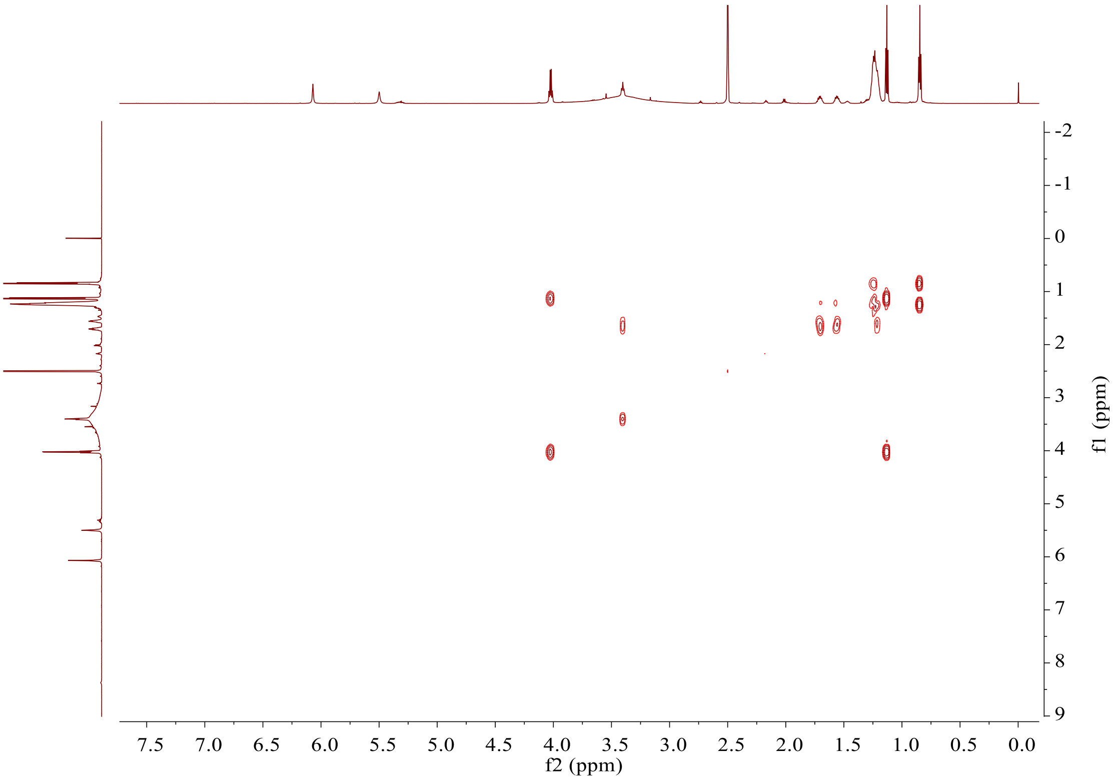

Fig. S31 COSY spectrum of **9** in DMSO-d_6.

Mass Spectrum SmartFormula Report

**Analysis Info**		Acquisition Date	2/24/2021 5:44:14 PM	
Analysis Name	D:\Data\MS\data\202102\caijian_L-C3_pos_1_01_10025.d			
Method	LC_Direct Infusion_pos_70-500mz.m	Operator	SCSIO	
Sample Name	caijian_L-C3_pos	Instrument	maXis	255552.00029
Comment				

Acquisition Parameter

Source Type	ESI	Ion Polarity	Positive	Set Nebulizer	0.4 Bar
Focus	Active	Set Capillary	4500 V	Set Dry Heater	180 °C
Scan Begin	70 m/z	Set End Plate Offset	-500 V	Set Dry Gas	4.0 l/min
Scan End	1500 m/z	Set Charging Voltage	0 V	Set Divert Valve	Waste
		Set Corona	0 nA	Set APCI Heater	0 °C

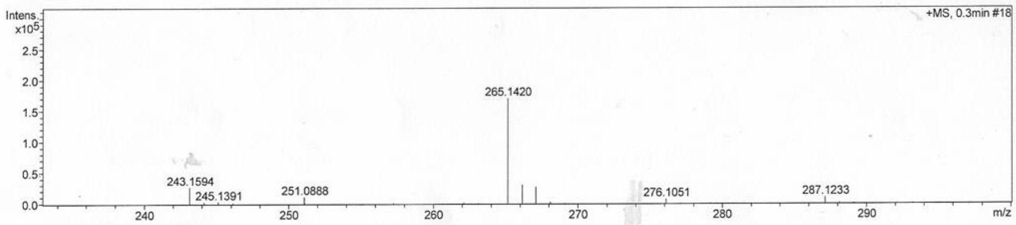

Meas. m/z	#	Ion Formula	Score	m/z	err [ppm]	err [mDa]	mSigma	rdb	e⁻ Conf	N-Rule
243.1594	1	C13H23O4	100.00	243.1591	1.5	0.4	16.8	2.5	even	ok
265.1420	1	C13H22NaO4	100.00	265.1410	3.5	0.9	23.5	2.5	even	ok
287.1233	1	C13H21Na2O4	100.00	287.1230	-1.0	-0.3	32.8	2.5	even	ok
507.2902	1	C26H44NaO8	100.00	507.2928	5.3	2.7	29.6	4.5	even	ok
529.2734	1	C26H43Na2O8	100.00	529.2748	2.6	1.4	32.9	4.5	even	ok

caijian_L-C3_pos_1_01_10025.d
Bruker Compass DataAnalysis 4.1 printed: 2/24/2021 10:11:21 PM by: SCSIO Page 1 of 1

Fig. S32 HRESIMS spectrum of compound **9**.

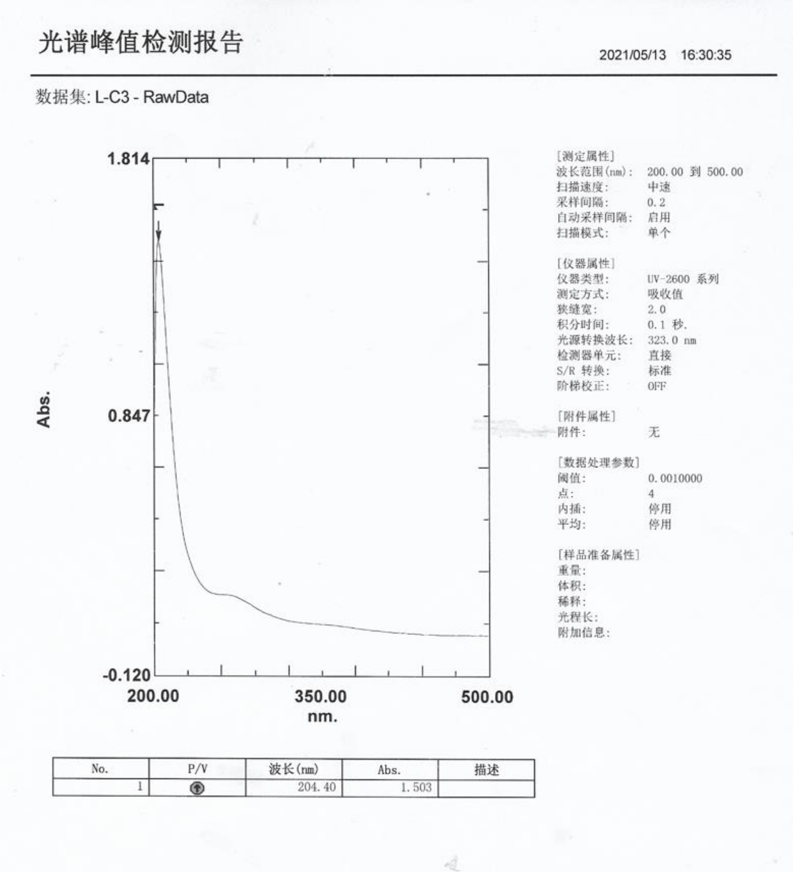

Fig. S33 The UV spectrum of **9**.

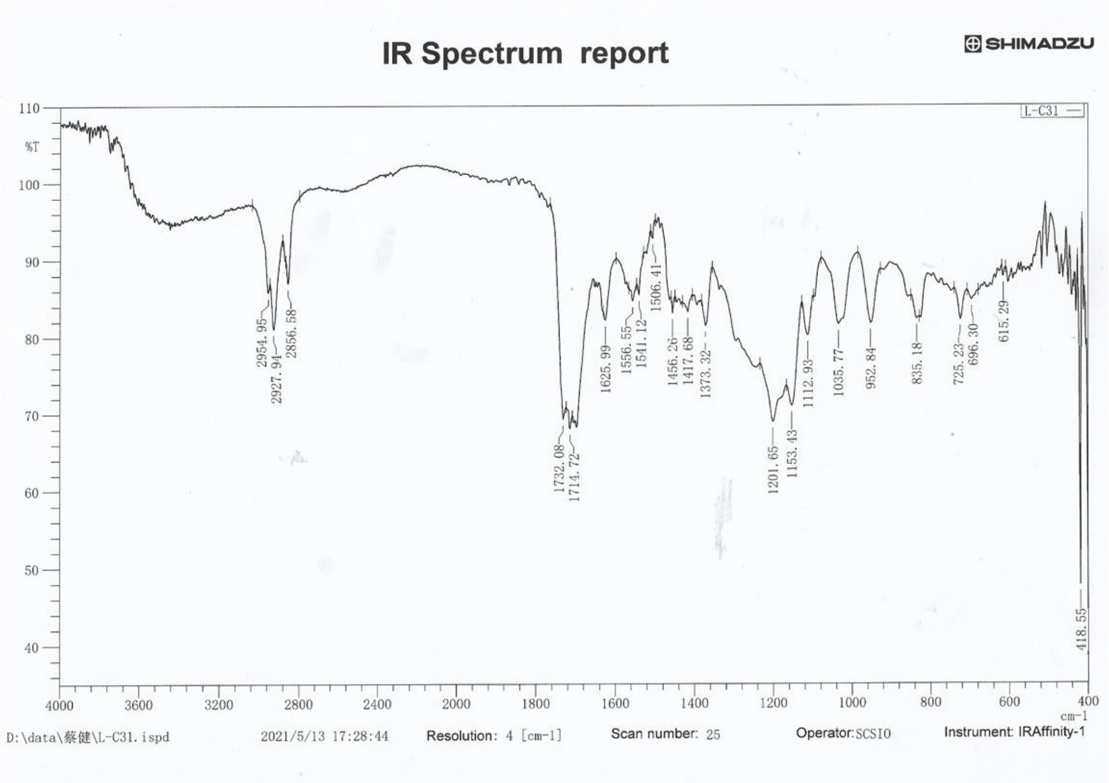

Fig. S34 The IR spectrum of **9**.

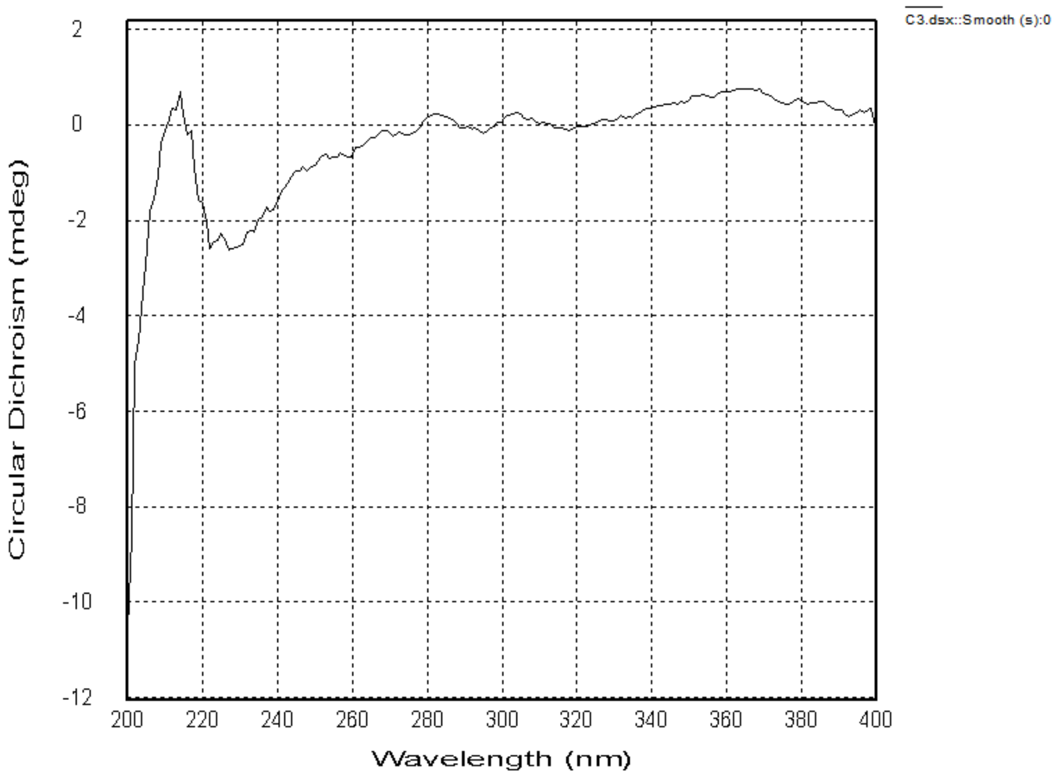

Fig. S35 The CD spectrum of **9** in MeOH.

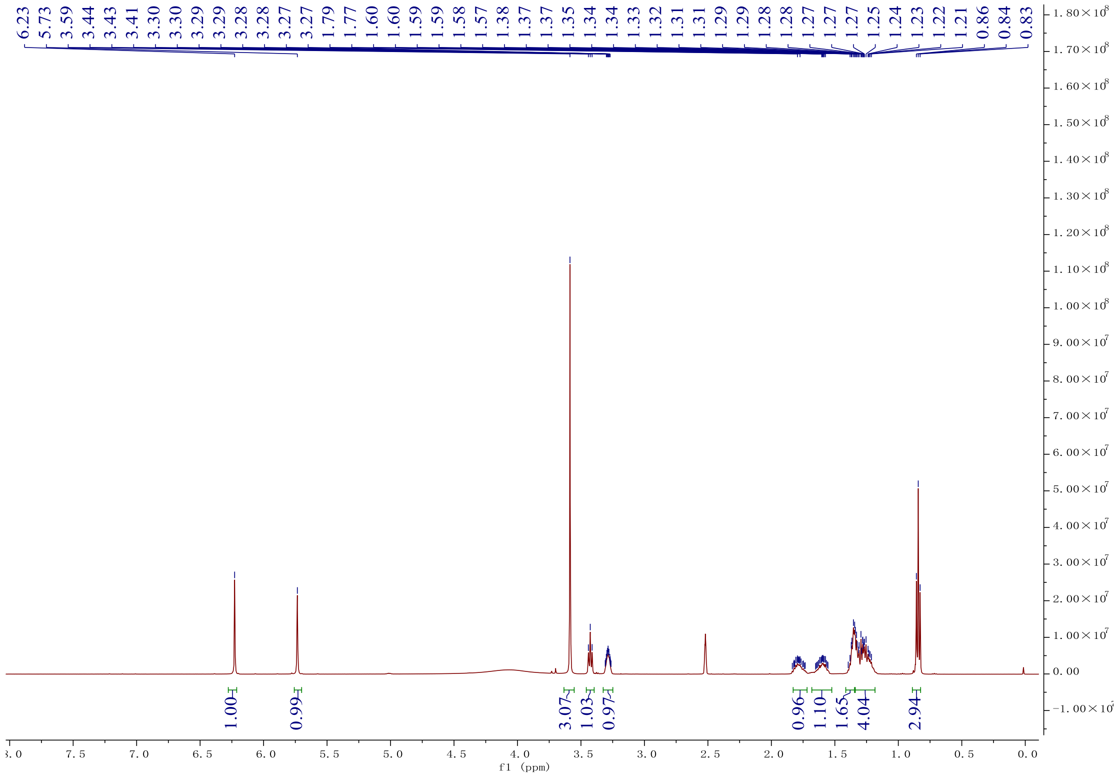

Fig. S36 ^{1}H NMR spectrum of **10** in DMSO-d_6

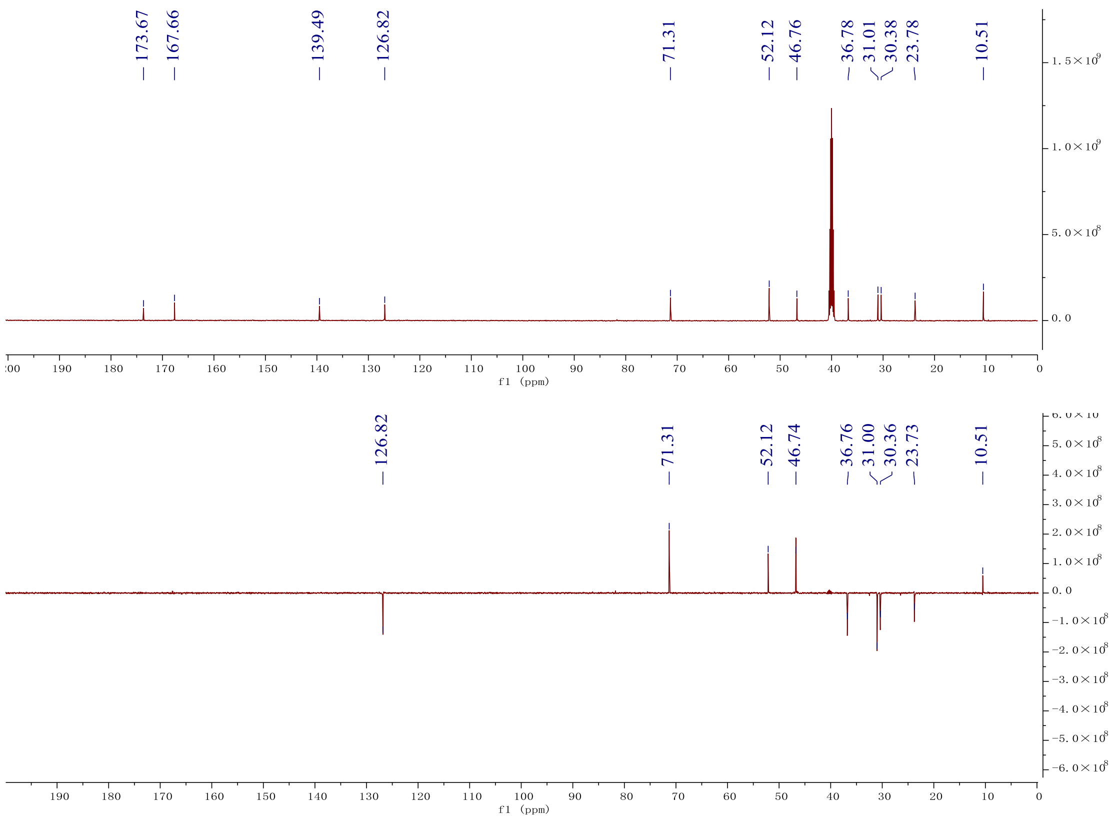

Fig. S37 ^{13}C and DEPT NMR spectra of **10** in DMSO-d_6

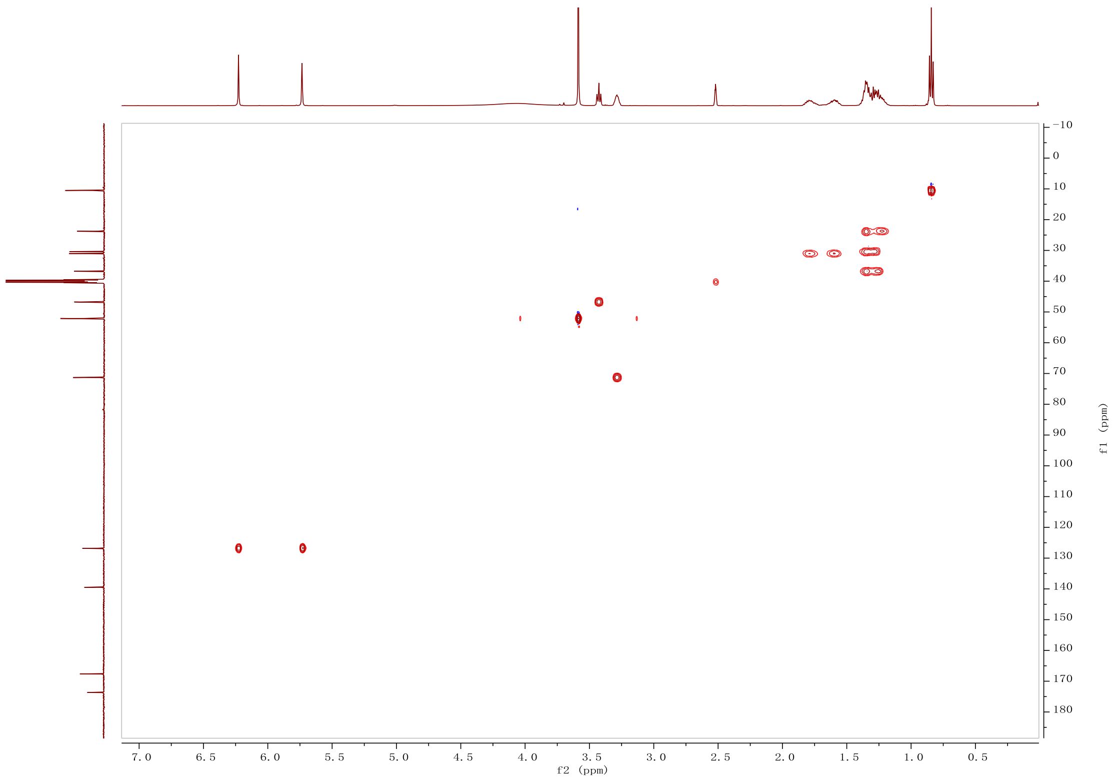

Fig. S38 HSQC spectrum of **10** in DMSO-d_6

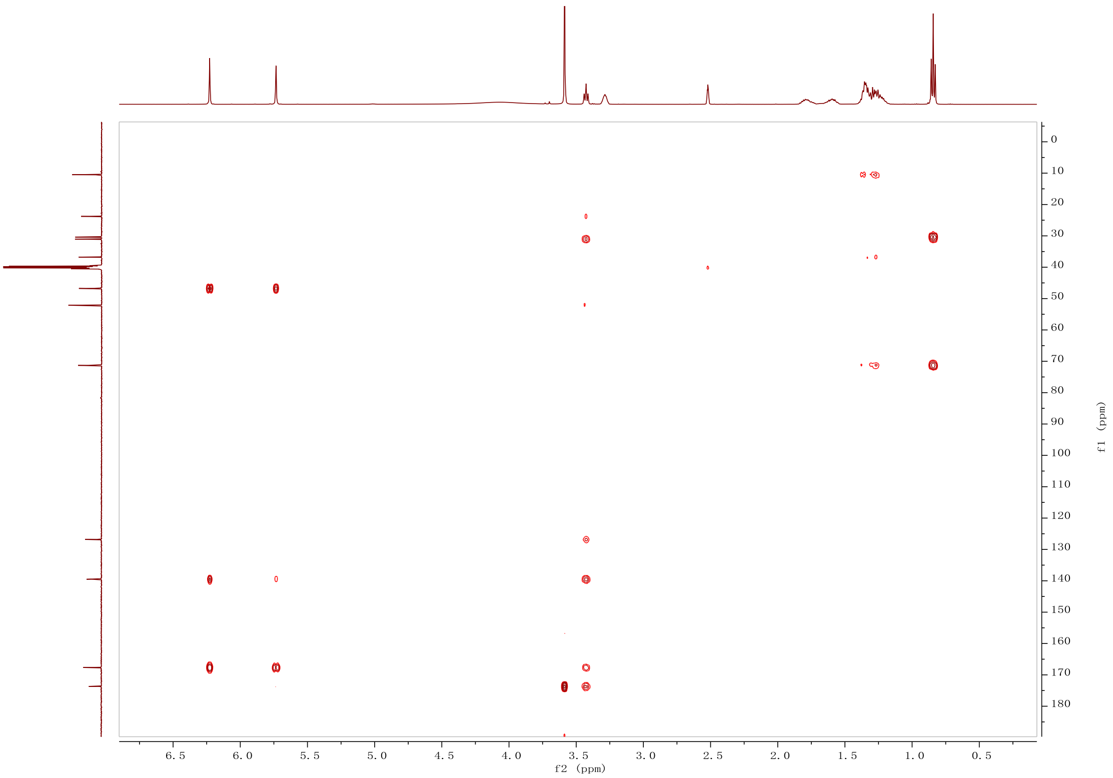

Fig. S39 HMBC spectrum of **10** in DMSO-d_6

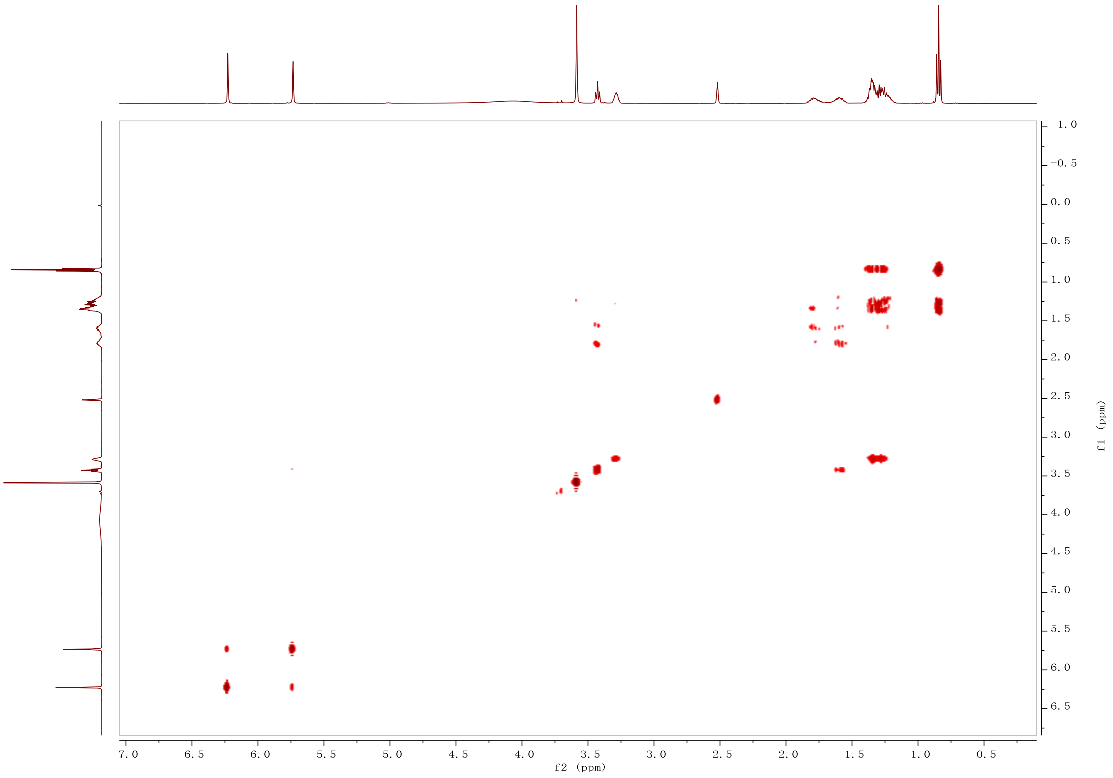

Fig. S40 COSY spectrum of **10** in DMSO-d_6

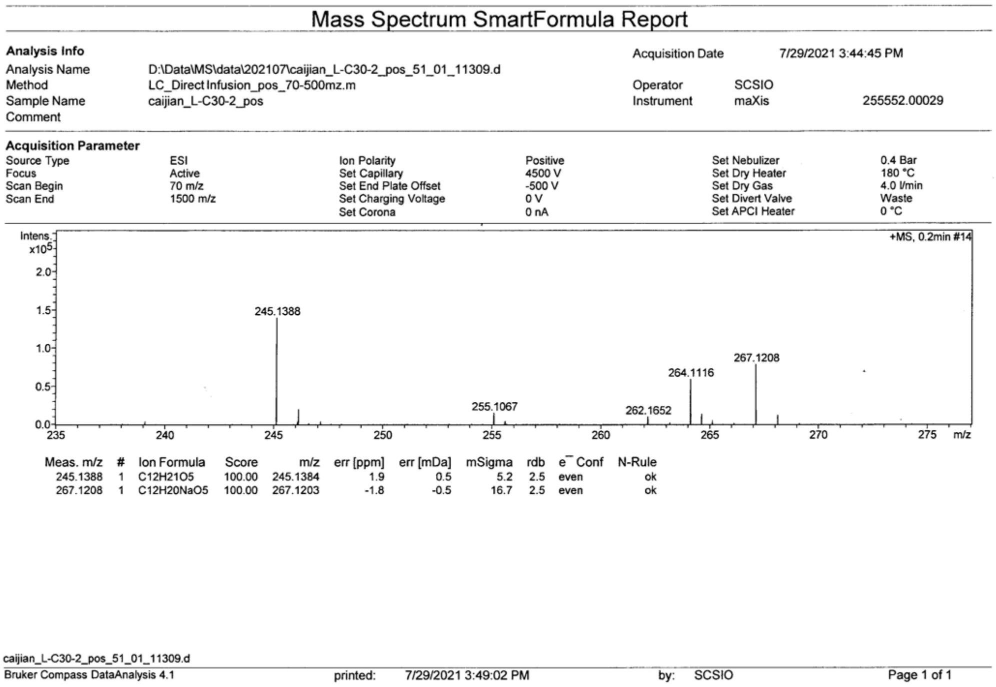

Fig. S41 HRESIMS spectrum of compound **10**.

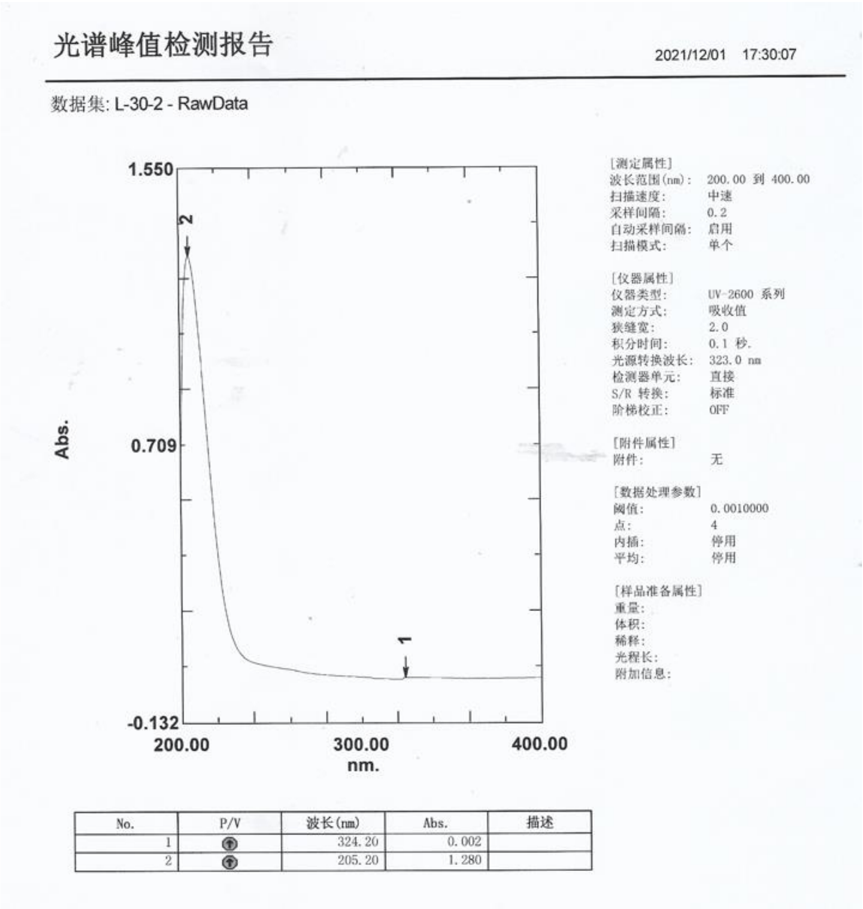

Fig. S42 The UV spectrum of **10**.

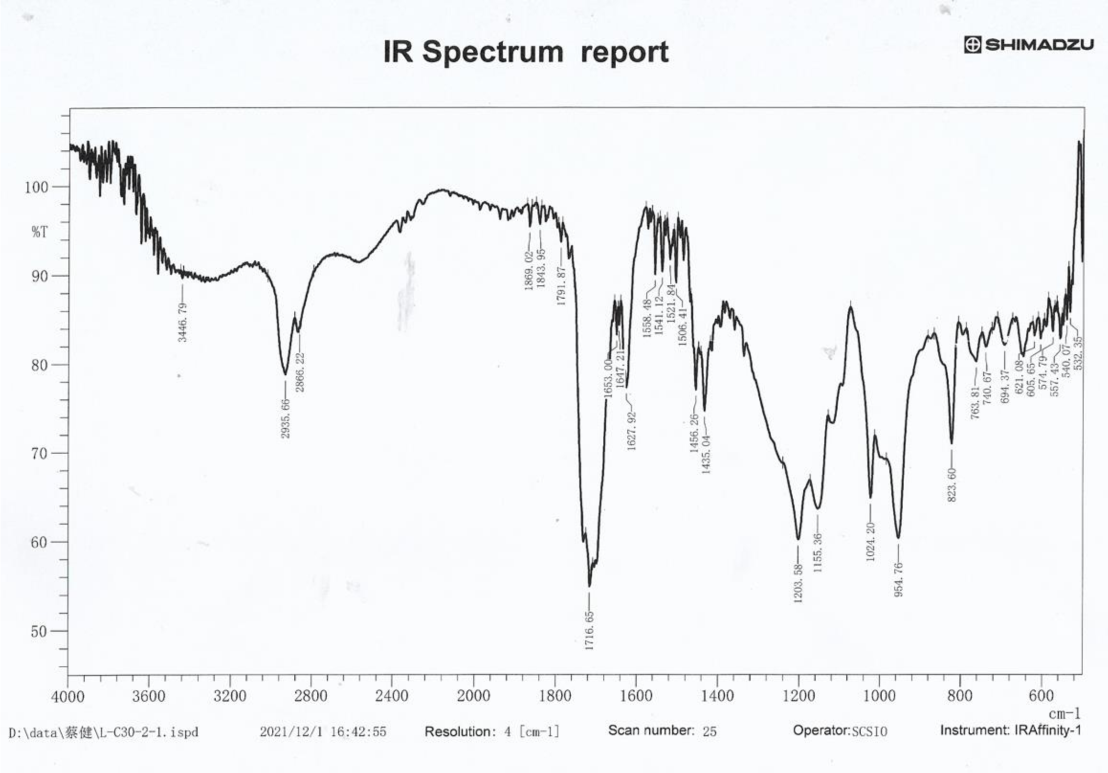

Fig. S43 The IR spectrum of **10**.

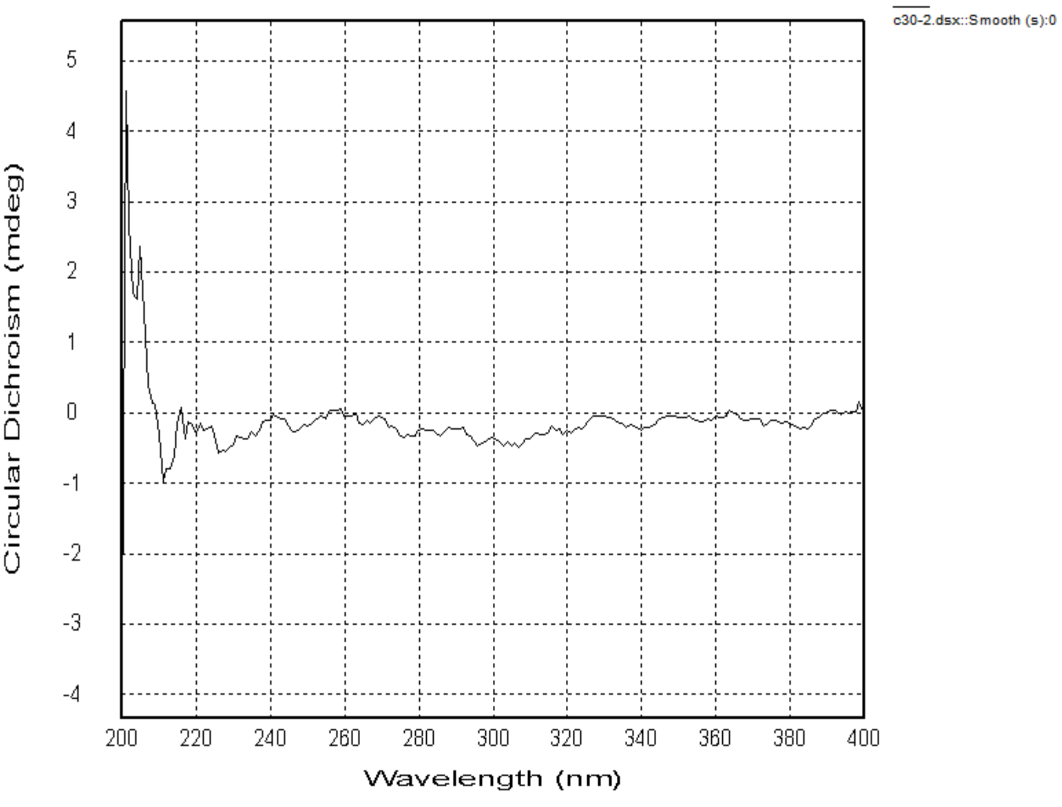

Fig. S44 The CD spectrum of **10** in MeOH.

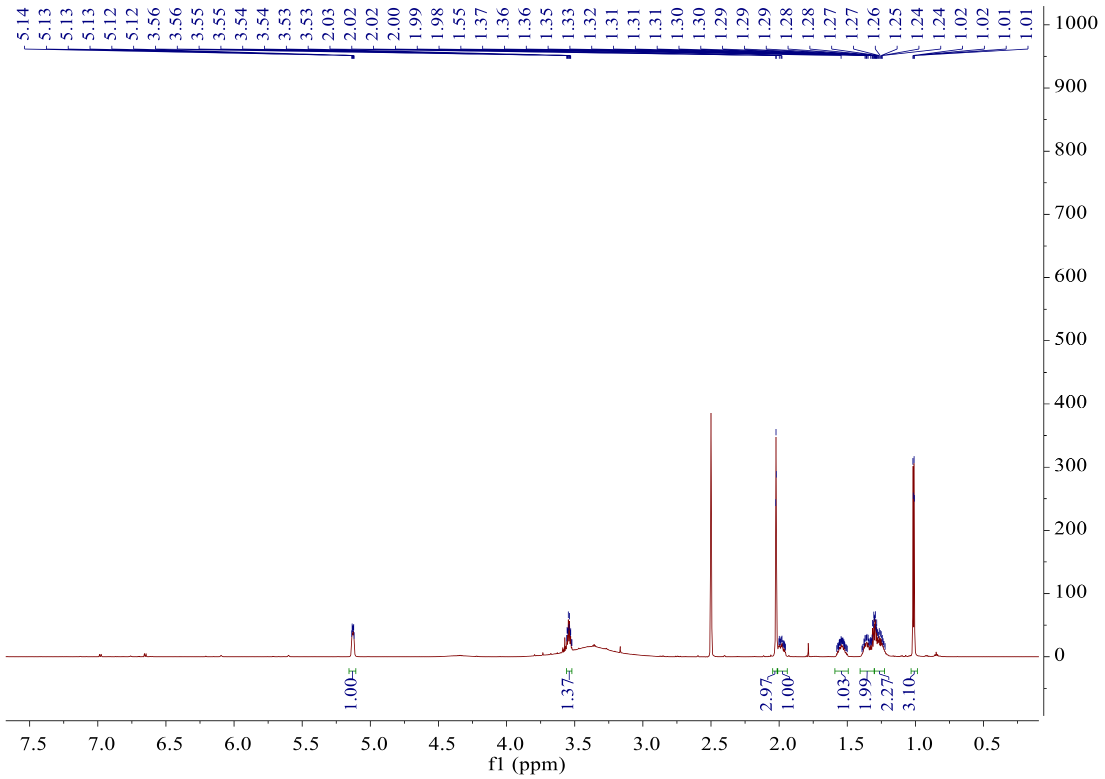

Fig. S45 ^{1}H NMR spectrum of **11** in DMSO-d_6.

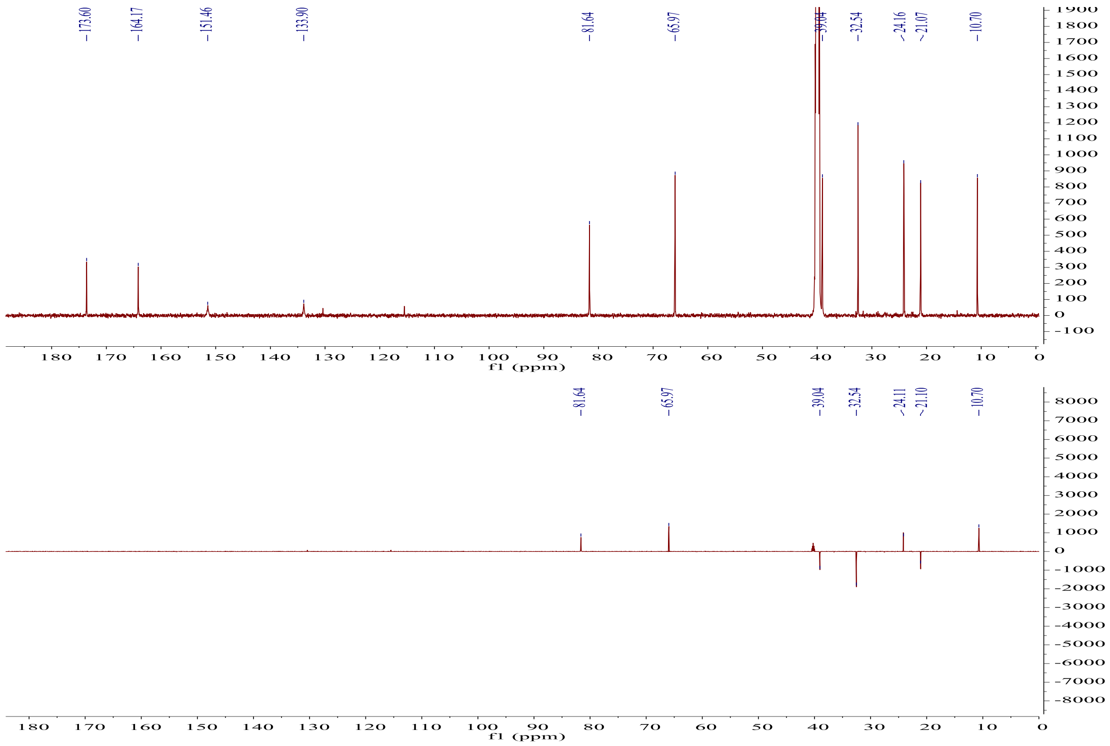

Fig. S46 ^{13}C and DEPT NMR spectra of **11** in DMSO-d_6.

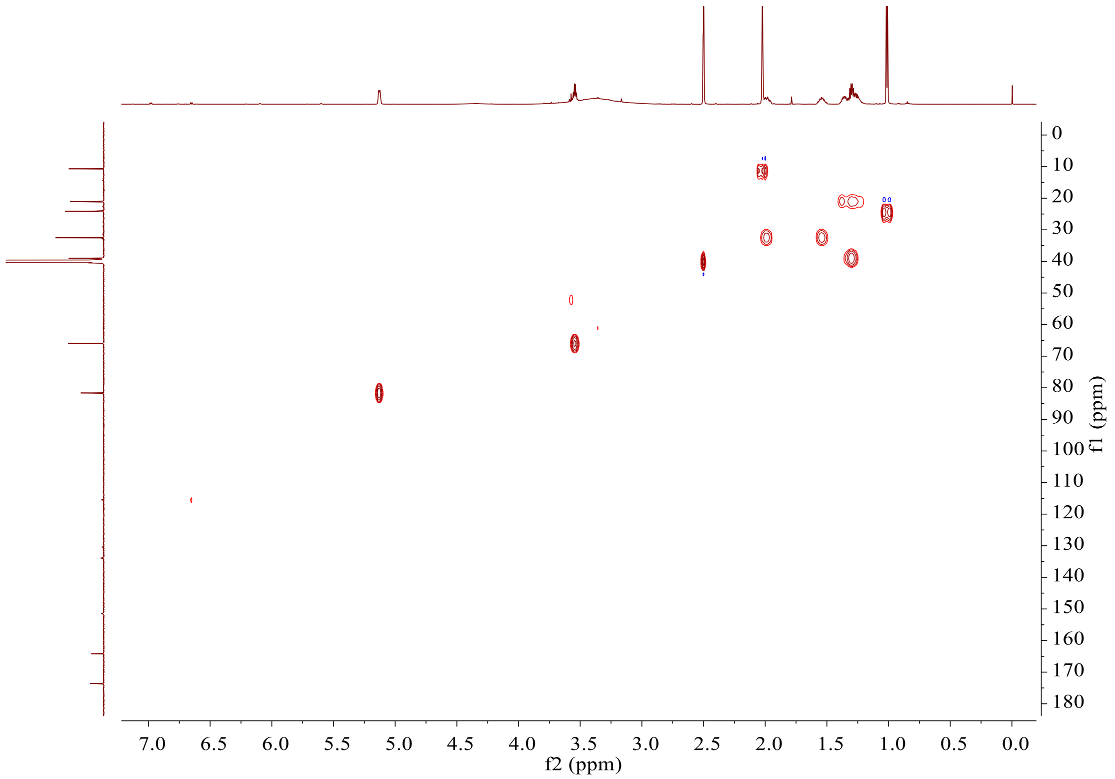

Fig. S47 HSQC spectrum of **11** in DMSO-d_6.

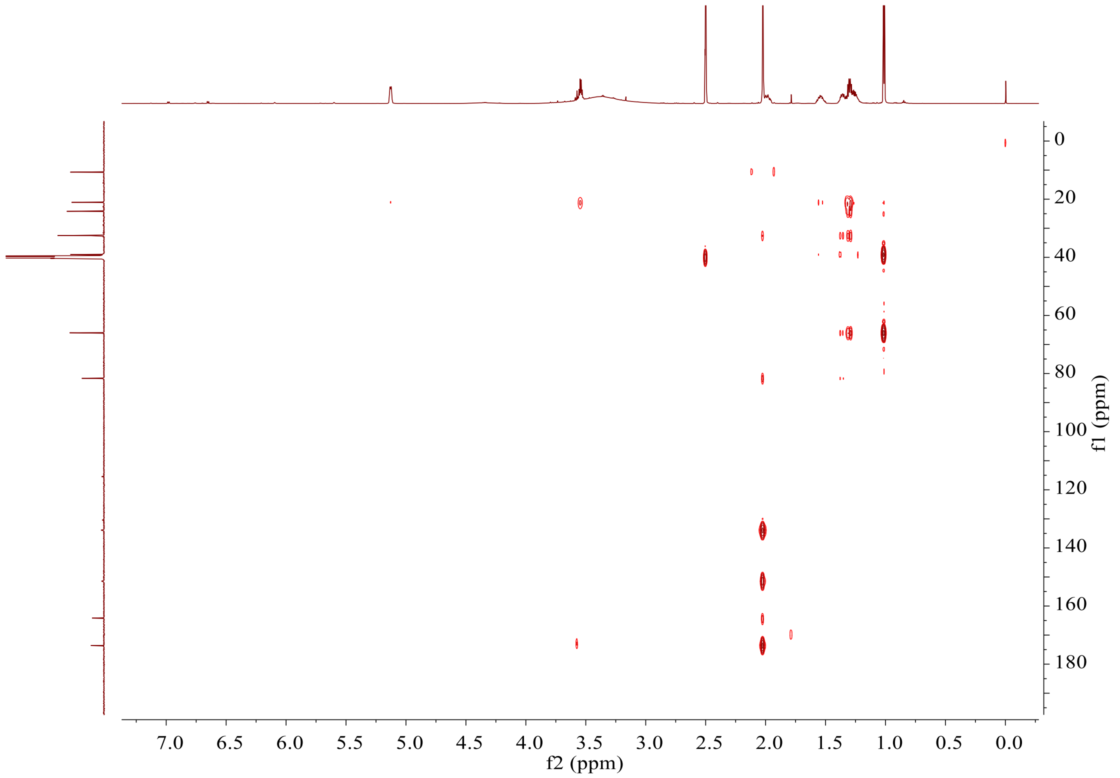

Fig. S48 HMBC spectrum of **11** in DMSO-d_6.

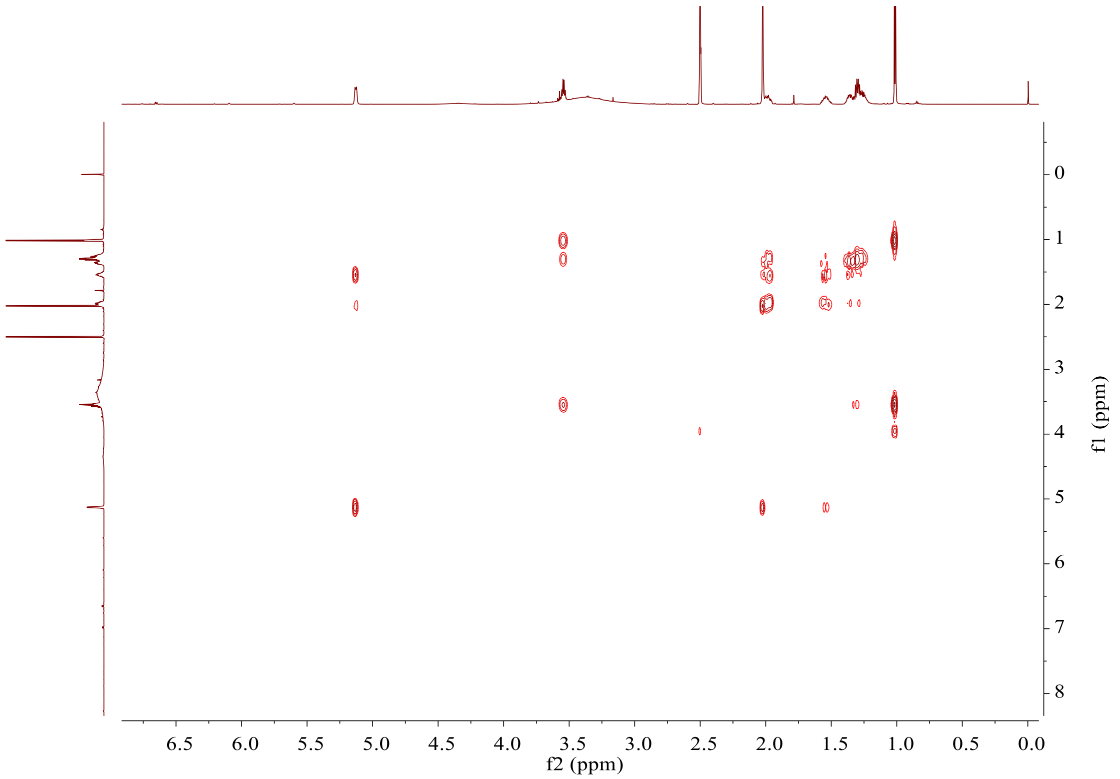

Fig. S49 COSY spectrum of **11** in DMSO-d_6.

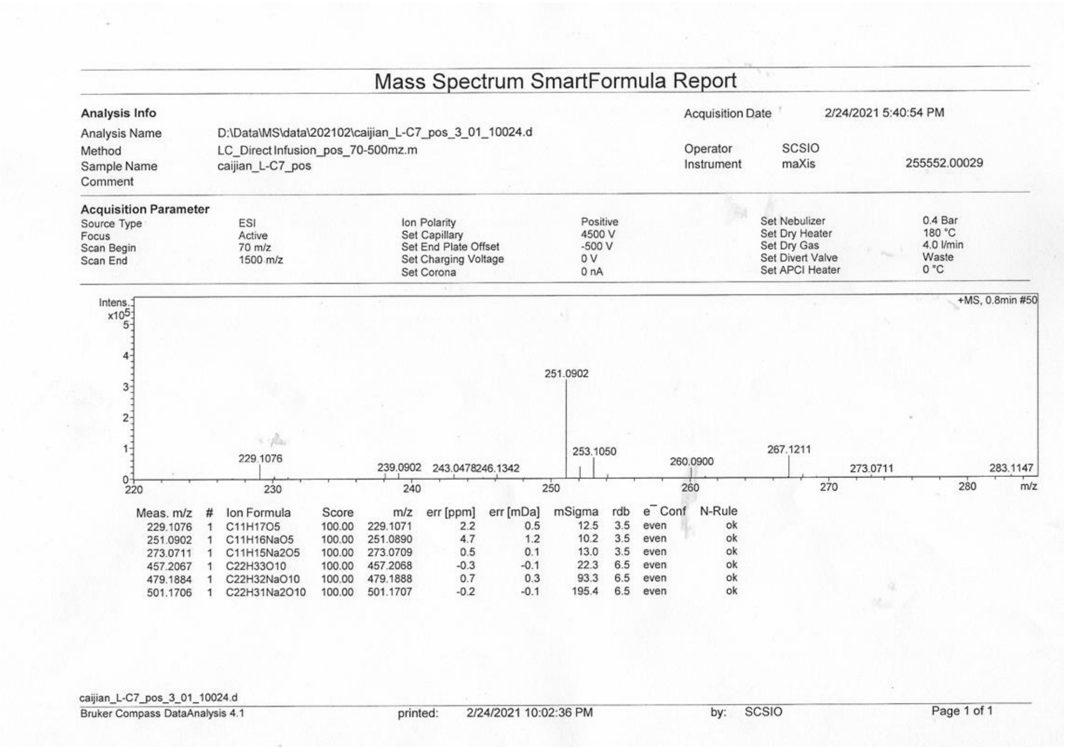

Mass Spectrum SmartFormula Report

Analysis Info

Analysis Name	D:\Data\MS\data\202102\caijian_L-C7_pos_3_01_10024.d	Acquisition Date	2/24/2021 5:40:54 PM
Method	LC_Direct Infusion_pos_70-500mz.m	Operator	SCSIO
Sample Name	caijian_L-C7_pos	Instrument	maXis 255552.00029
Comment			

Acquisition Parameter

Source Type	ESI	Ion Polarity	Positive	Set Nebulizer	0.4 Bar
Focus	Active	Set Capillary	4500 V	Set Dry Heater	180 °C
Scan Begin	70 m/z	Set End Plate Offset	-500 V	Set Dry Gas	4.0 l/min
Scan End	1500 m/z	Set Charging Voltage	0 V	Set Divert Valve	Waste
		Set Corona	0 nA	Set APCI Heater	0 °C

Meas. m/z	#	Ion Formula	Score	m/z	err [ppm]	err [mDa]	mSigma	rdb	e⁻ Conf	N-Rule
229.1076	1	C11H17O5	100.00	229.1071	2.2	0.5	12.5	3.5	even	ok
251.0902	1	C11H16NaO5	100.00	251.0890	4.7	1.2	10.2	3.5	even	ok
273.0711	1	C11H15Na2O5	100.00	273.0709	0.5	0.1	13.0	3.5	even	ok
457.2067	1	C22H33O10	100.00	457.2068	-0.3	-0.1	22.3	6.5	even	ok
479.1884	1	C22H32NaO10	100.00	479.1888	0.7	0.3	93.3	6.5	even	ok
501.1706	1	C22H31Na2O10	100.00	501.1707	-0.2	-0.1	195.4	6.5	even	ok

caijian_L-C7_pos_3_01_10024.d
Bruker Compass DataAnalysis 4.1 printed: 2/24/2021 10:02:36 PM by: SCSIO Page 1 of 1

Fig. S50 HRESIMS spectrum of compound **11**.

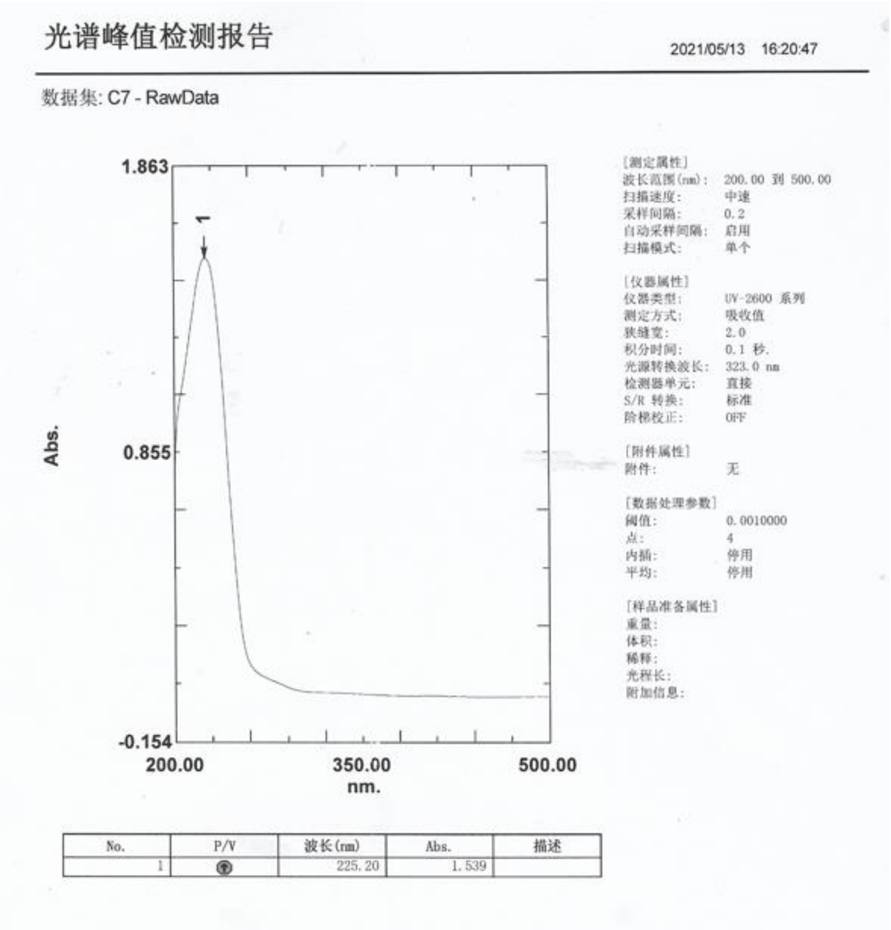

Fig. S51 The UV spectrum of **11**.

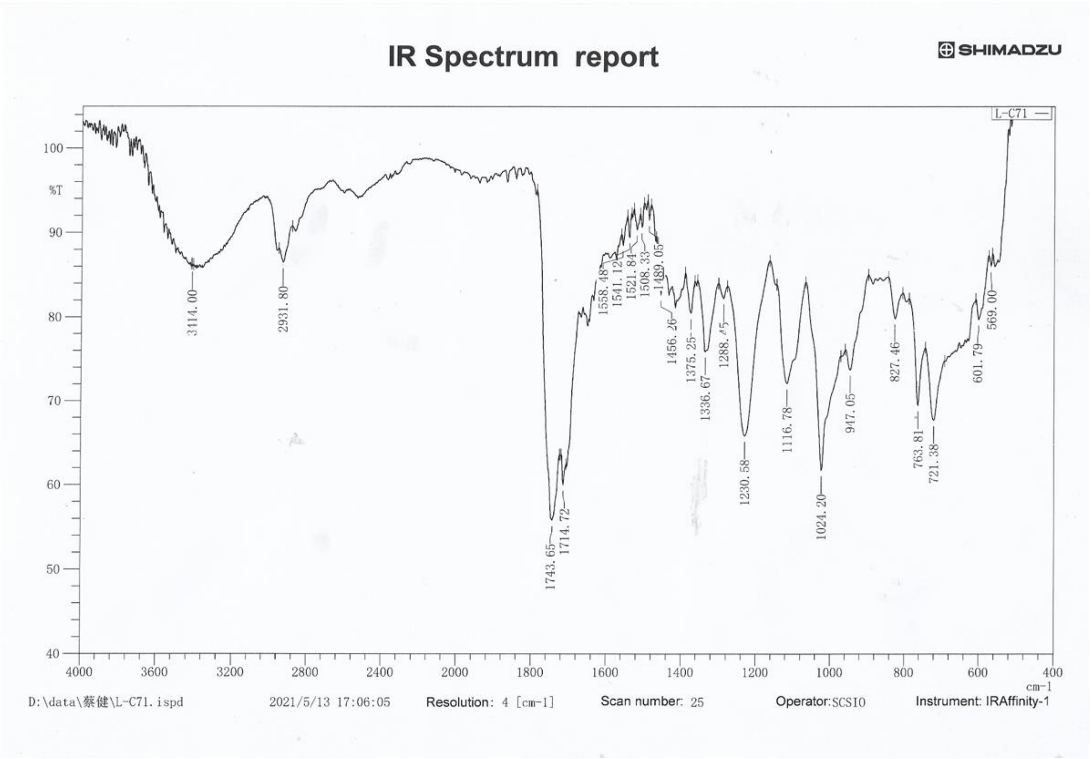

Fig. S52 The IR spectrum of **11**.

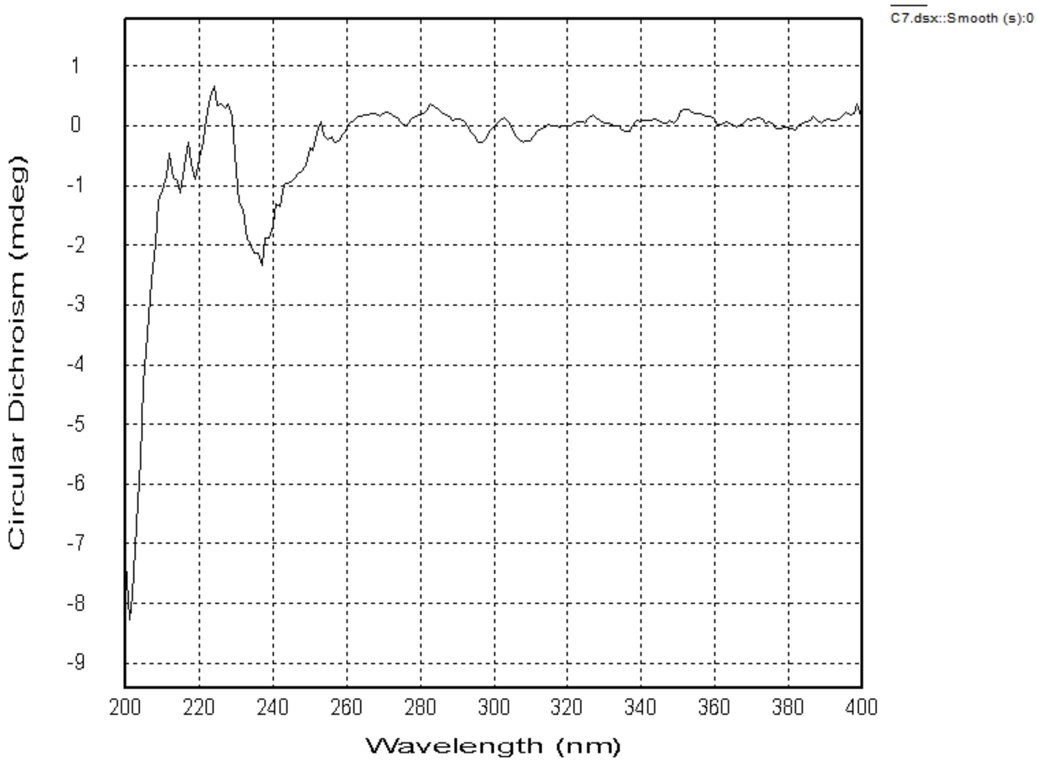

Fig. S53 The CD spectrum of **11** in MeOH.
