# Supplementary material for: Thiodiketopiperazines and Alkane Derivatives Produced by the Mangrove Sediment–Derived Fungus Penicillium ludwigii SCSIO 41408
Source: Front Microbiol. 2022 Mar 28;13:857041. doi: 10.3389/fmicb.2022.857041 (PMC8996154; doi:10.3389/fmicb.2022.857041)
Supplement: Supplementary file 2 [file Data_Sheet_2.PDF]

Dear Prof. Yang,

Thank you very much for your comments on our manuscript (ID: 857041). Based on your and reviewers' comments and requests, we have carefully made corresponding revisions, which we hope meet with your approval. The detailed corrections are listed below point by point.

Reviewer 1:

1. In Abstract, the authors indicated that compounds 1–5 exhibited "selective" antifungal or antibacterial activities, it's better to have a corresponding discussion in the "Results and Discussion" section. In addition, a variety of bioactivity screening was evaluated, it is suggested that the author discuss the purpose and significance of this study in detail in the Introduction section.

Respond: We are sorry to have missed some data that was not written in the text. In fact, the experiments tested five types of bacteria and four types of fungi, and the missing three bacteria and two fungi have been added to the article. Line138-141, "The antimicrobial activities against five bacteria (*Erysipelothrix rhusiopathiae* WH13013, *Streptococcus suis* SC19, *Escherichia coli* ATCC 25922, *Pseudomonas aeruginosa* ATCC 27853, and *Staphylococcus aureus* ATCC 25923) and four fungi (*Botrytis cinerea*, *Septoria nodorum* Berk., *Fusarium graminearum* Schw., and *Rhizoctonia solani* Kühn)"

In the "Results and Discussion", line 253-255, "However, all compounds showed no activities against the other three bacteria (*Escherichia coli* ATCC 25922, *Pseudomonas aeruginosa* ATCC 27853, and *Staphylococcus aureus* ATCC 25923) and two fungi (*Fusarium graminearum* Schw. and *Rhizoctonia solani* Kühn)."

In the "Introduction", line 50-54, "We performed screening for antibacterial and antifungal activities, cytotoxicity against anti-prostate cancer cells, and inhibitory activities of LPS-induced NF-κB activation. NF-κB exhibits an important role in RANKL-induced osteoclast differentiation (Hong et al., 2020; Tan et al., 2020; Zhou et al., 2020). Our further research found that Adametizine C (**1**) was suggested to be the promising lead compound for the treatment of osteoclast-related diseases."

2. Please reprocess the NMR spectra of the compounds. The number of H and C of some compounds after processing did not agree with the chemical formula. Such as compounds 1, 9, etc. Especially compound 1, lines 97 indicated HRESIMS m/z 563.0381 [M + H]+ (calcd for C21H24ClN2O8S3, 563.0378), while line 174, indicated "compound 1 had the molecular formula C21H22ClN2O8S3". Please check this and others.

Respond: Thanks for pointing out our mistake, we rechecked the full text and made corrections. Line 220, "C21H22ClN2O8S3" modified to"C21H23ClN2O8S3"; line 216, "C13H22O4" modified to "C13H23O4".

3. It is suggested to resupply Figure 4A, and can the authors explain the increasing number of colonies treated with 3 in 10 μM in Figure 4C?

Respond: Thanks for your comments and thanks for giving us the opportunity to explain this figure. The plate clone formation assay was independently repeated three times in this study, and the results are shown in the following figure plates 1-3. In each experiment, the number of colonies of control group was significantly higher than experimental groups. However, the number of colonies between experimental groups were extremely close. As shown in the following table, compound **3** showed strong activity at 1.25 μM, and the number of colonies ranged from 1 to 3. Therefore, the numbers of colonies were too small at 2.5 μM, 5 μM, and 10 μM, ranging from 0-1. Thus, we think that it was an experimental error within an acceptable range between treatment group of 5 μM and 10 μM in figure 4A and 4C. It should also be noted that we excluded the number of colonies of plate 3 when counting the number of colonies in the control group due to that it is much smaller (difference greater than 10%) than both the other two sets of values.

We provided raw data from 3 replicate experiments (**Fig S1**):

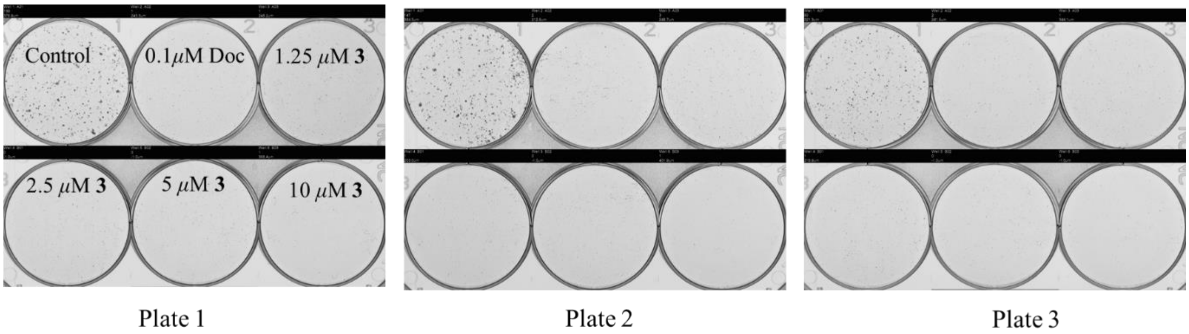

Number of colonies

	Plate 1	Plate 2	Plate 3
Control	139	147	80
Doc（1 μM）	1	2	2
**3**（1.25 μM）	1	3	1
**3**（2.5 μM）	0	1	1
**3**（5 μM）	0	0	0
**3**（10 μM）	1	1	0

Fig S1. Raw data from 3 replicate experiments:

4. Please check and correct the legends in Figure 1 and 2.

Respond: The legends in Figure 1 and 2 has been corrected in the paper.

5. Please redrawn the structures of compounds 1−6, unify the representation of methyl groups.

Respond: The structures of compounds 1−6 have been unified the representation of methyl groups in **fig. 1.**

6. Line 254, 255, “Figure 8 A−D” should be corrected to “Figure 4 A−D”.

Respond: Line 263,264, “Figure 8 A−D” has been corrected to “Figure 4 A−D”

7. There are so many mistakes in the manuscript. For examples, in Abstract, line 27, with “an” IC50 value of; line 31, evaluated for “their” effects; line 87, remove the repeated “four”; line 161,162, problems with spacing between words; line 163, recorded; line 166, 2.0×105; line 174, needles; line 247, controls; line 253, plate; In Supplementary Material, check the spelling, compounds 2-3, “withe powder” or “white powder”? These are enumeration, not exhaustive. It should be carefully polish, check and correct thoroughly.

Respond: Thanks for your careful checks. We are sorry for our carelessness. Base on your comments, we have made the corrections to make the unit harmonized within the whole manuscript. for examples,
Line 27, “an” IC50 value of; line 31, evaluated “their” effects; line 91, remove the repeated “four”;
line 167,168, corrected the problems with spacing between words; line 169, “record” corrected to “recorded”; line 172,“*” corrected to“×”; line 180, corrected to “needles”; line 256， corrected to“controls”; line 262, corrected to “plate”; In the “Supplementary Material”, compounds 2-3, “withe powder” corrected to “white powder”. Not listed exhaustively, some other bugs have been corrected in the article.

Reviewer 2:

1. What was the basis for the two bacteria chosen in the antibacterial experiment? Both bacteria are gram-positive, why not try gram-negative bacteria?

Respond: We are sorry to have missed some data that was not written in the text. In fact, the experiments tested five types of bacteria and four types of fungi, and the missing three bacteria (including gram-negative bacteria) and two fungi have been added to the article, Line138-141, “The antimicrobial activities against five bacteria (*Erysipelothrix rhusiopathiae* WH13013, *Streptococcus suis* SC19, *Escherichia coli* ATCC 25922, *Pseudomonas aeruginosa* ATCC 27853, and *Staphylococcus aureus* ATCC 25923) and four fungi (*Botrytis cinerea*, *Septoria nodorum* Berk., *Fusarium graminearum* Schw., and *Rhizoctonia solani* Kühn)”

2. In the antibacterial, toxicity, and other activity test results, it is recommended to add more detailed descriptions, and some relevant literature can be added for comparative analysis.

Respond: In the “Results and Discussion”, we added some descriptions and analysis. Line 253-255, “However, all compounds showed no activities against the other three bacteria (*Escherichia coli* ATCC 25922, *Pseudomonas aeruginosa* ATCC 27853, and *Staphylococcus aureus* ATCC 25923) and two fungi (*Fusarium graminearum* Schw. and *Rhizoctonia solani* Kühn).”. Line 274-280, “In addition, we also found that the thiodiketopiperazine derivatives exhibited exhibited various activities reported in the literatures. For example, pretrichodermide A was active against *Mycobacterium tuberculosis* (Seephonkai et al., 2006); Adametizine A (**2**) was found to be active against a variety of bacteria (Liu et al., 2015); Outovirin C was active against fungus *Botrytis cinerea* (Kajula et al., 2016). Gliovirin showed inhibitory effects on the expression of cytokines (TNF-α and IL-2) and pro-inflammatory enzymes (COX-2, INOS) in T-cells and monocytes/macrophages (Rether et al., 2007).”

3. It is suggested to add the title of the conclusion, and the conclusion part is too similar to the introduction part, it is suggested to revise.

Respond: We have added the title of the conclusion and revised the content. Line 281-290, “In summary, chemical investigation of the mangrove sediment-derived fungus *Penicillium ludwigii* SCSIO41408 led to the isolation of a new trithiodiketopiperazine, five new alkane derivatives (**7–11**), and five dithiodiketopiperazine derivatives (**2–6**). In a variety of bioactivity screening, **1**–**5** exhibited some selective antifungal or antibacterial activities. **1**–**3** showed cytotoxicity against prostate cancer cell line 22Rv1or PC-3 cells, moreover **3** could significantly reduce PC-3 cells colony formation and induce apoptosis in a dose-dependent manner. Several compounds also exhibited obvious inhibitory activities of LPS-induced NF-κB, and **1**, **5**, and **9** were suppressed RANKL induced osteoclast differentiation in BMMCs at 10 μM. Adametizine C (**1**), with the strongest inhibitory activity against RANKL-induced osteoclast differentiation, was suggested to be the promising lead compound for the treatment of osteoclast-related diseases.”

4. It is recommended to add some references, the number of references is not enough.

Respond: Some references have been added in the article
